# Rational approach to guest confinement inside MOF cavities for low-temperature catalysis

**DOI:** 10.1038/s41467-019-08972-x

**Published:** 2019-03-22

**Authors:** Tiesheng Wang, Lijun Gao, Jingwei Hou, Servann J. A. Herou, James T. Griffiths, Weiwei Li, Jinhu Dong, Song Gao, Maria-Magdalena Titirici, R. Vasant Kumar, Anthony K. Cheetham, Xinhe Bao, Qiang Fu, Stoyan K. Smoukov

**Affiliations:** 1 Department of Materials Science and Metallurgy, University of Cambridge, Cambridge, CB3 0FS UK; 2 EPSRC Centre for Doctoral Training in Sensor Technologies and Applications, University of Cambridge, Cambridge, CB3 0AS UK; 3 School of Chemistry, The University of Sydney, Sydney, NSW 2006 Australia; 4 State Key Laboratory of Catalysis, iChEM, Dalian Institute of Chemical Physics, Chinese Academy of Sciences, Dalian, 116023 People’s Republic of China; 5 UNESCO Centre for Membrane Science and Technology, School of Chemical Engineering, The University of New South Wales, Sydney, NSW 2052 Australia; 6 School of Engineering and Materials Science, Queen Mary University of London, London, E1 4NS UK; 7 Materials Research Institute, Queen Mary University of London, London, E1 4NS UK; 8 Department of Chemical Engineering, Imperial College London, London, SW7 2AZ UK; 9 Department of Materials Science and Engineering, National University of Singapore, Singapore, 117574 Singapore; 10 Department of Chemical and Pharmaceutical Engineering, Faculty of Chemistry and Pharmacy, Sofia University, Sofia, 1164 Bulgaria

## Abstract

Geometric or electronic confinement of guests inside nanoporous hosts promises to deliver unusual catalytic or opto-electronic functionality from existing materials but is challenging to obtain particularly using metastable hosts, such as metal–organic frameworks (MOFs). Reagents (e.g. precursor) may be too large for impregnation and synthesis conditions may also destroy the hosts. Here we use thermodynamic Pourbaix diagrams (favorable redox and pH conditions) to describe a general method for metal-compound guest synthesis by rationally selecting reaction agents and conditions. Specifically we demonstrate a MOF-confined RuO_2_ catalyst (RuO_2_@MOF-808-P) with exceptionally high catalytic CO oxidation below 150 °C as compared to the conventionally made SiO_2_-supported RuO_2_ (RuO_2_/SiO_2_). This can be caused by weaker interactions between CO/O and the MOF-encapsulated RuO_2_ surface thus avoiding adsorption-induced catalytic surface passivation. We further describe applications of the Pourbaix-enabled guest synthesis (PEGS) strategy with tutorial examples for the general synthesis of arbitrary guests (e.g. metals, oxides, hydroxides, sulfides).

## Abstract (web summary)

Loading guests inside the pre-existing pores of nanoporous hosts remains challenging. Here, the authors introduce a rational route for incorporation of guest compounds into an arbitrary nanoporous host, enabling the investigation of multiple host-guest systems with surprising functionalities.

## Introduction

Loading guests (e.g., molecules, clusters or particles) inside the pre-existing pores of nanoporous hosts (guest@nanoporous-host) is one of the key post-synthesis modification strategies for porous materials^1–17^. It can yield highly active and stable heterogeneous catalysts^2–4,9–17^ as well as robust photo/electro-luminescence materials^2,5,6,18^ with tunable band structures in quantum confinement. Forming guests within the pores has been extensively explored using inorganic nanoporous materials^1,2,10^ and supramolecular cages^5,11^ (i.e., host–guest chemistry and/or inclusion chemistry). Besides metal particles/clusters^4,13,14,16,19^, however, such synthesis is still challenging or impossible for other types of guest particles/clusters (e.g., oxides, hydroxides, sulfides, nitrides, phosphides) inside the host’s cavity/channel. Many hosts have very small aperture (a.k.a. window) opening sizes (typically <2 nm), and hence direct impregnation of guest compounds with much larger sizes is no longer feasible. Guests, therefore, have to be assembled locally within the cavity/channel (i.e., ship-in-a-bottle assembly^9,14,20^). The general ‘ship-in-a-bottle’ approach is to load smaller precursors (e.g., salts and organometallics) into pre-formed porous host materials via solution-based, gas-phase or mechanical-mixing impregnation, followed by either thermal/photochemical decomposition or redox reaction (with either strong redox reagents, e.g., hydrazine and NaBH_4_, or high-temperature treatment in reducing atmosphere, e.g., H_2_)^2,4^. These methods which are useful for bulk or nanostructure synthesis (i.e., unconfined systems) often fail to work properly in nanoporous hosts. The major dilemmas are that (i) many of the reactants are still too large to be impregnated and (ii) the conditions required to form a target guest may damage or destroy the host structure^4^. Nonetheless, the ship-in-a-bottle strategy has been recently recognized as a promising way to post-synthetically functionalize porous metal–organic frameworks (MOFs)^3,4,11–17^, which are host matrices assembled with metal centers and organic ligands with extremely diverse chemistries, topologies and pore architectures^21–26^. By immobilizing the guests inside MOFs, guest aggregation/fusion can be effectively prevented^4,19^. Meanwhile, MOF hosts have been found to influence the properties of the guests, e.g., modulation of electron–hole recombination rates for quantum dots^4^. Hence, there is a demand to carry out ship-in-a-bottle synthesis with sufficiently small reaction reagents under mild conditions, as many of these metastable MOFs suffer from poor chemical and thermal stability^2,4,22–25^. A rational route for incorporation of guest compounds into an arbitrary nanoporous host should enable the investigation of multiple host–guest systems with surprising functionalities.

**Fig. 1:**
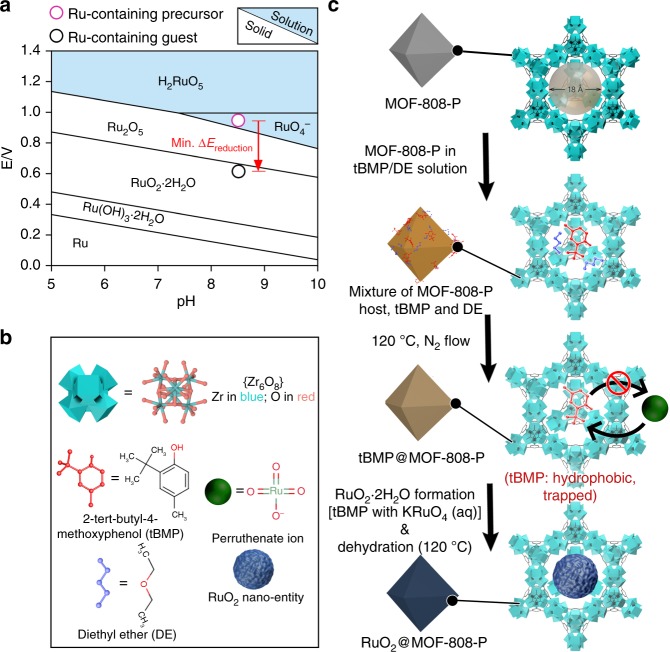
Pourbaix enabled guest synthesis (PEGS) strategy for RuO_2_ incorporation into MOF-808-P. **a** Pourbaix (redox potential-pH) diagram for Ru-H_2_O system (with a pH range of 5–10; concentration of Ru-based solution = 20 mM) constructed based on previously available data versus standard hydrogen electrode (SHE)^29^. Within the pH range it shows the range of potentials where a certain phase is thermodynamically stable, and the potential needed to transform one phase to another, i.e., the red arrow shows that to transform a soluble Ru-based precursor, perruthenate ion (RuO_4_^−^), to solid Ru-based guest (i.e., RuO_2_·2H_2_O) at a pH of ca. 8.5 (20 mM aqueous potassium perruthenate (KRuO_4_)), one needs minimum reduction potential (*ΔE*_reduction_) of 0.3–0.4 V (assuming an unaltered pH). A reductant, such as 2-tert-butyl-4-methylphenol (tBMP) with expected ca. 0.3 V to be oxidized, could be suitable. Diethyl ether (DE) is used as a solvent for tBMP. **b** Symbols for the scheme in (**c**), which illustrates RuO_2_ synthesis inside the cavity of pre-formed MOF-808-P using the hydrophobic reducing lipid tBMP. For clarity (i) the schematics of MOF-808-P is simplified as standard MOF-808^36^ and (ii) hydrogen atoms and carbon atoms for formates (HCOO^−^) are omitted in the metal–organic framework (MOF) cage

We realize that the synthetic conditions of guests can be pre-determinable based on pH/potential-dependent equilibrium solid/solution maps^27–31^ (well known by materials scientists as Pourbaix diagrams, e.g., Fig. 1a), which have been extensively investigated and used for depicting relevant thermodynamics during a corrosion process (normally solid → solution)^32^. Instead of studying solid → solution reactions, we use the Pourbaix diagrams to select precursor solutions and synthetic conditions (i.e., redox potential and/or pH) to solidify the desired guests (i.e., solution → solid processes) within the pores of the hosts^30^. We term this strategy Pourbaix enabled guest synthesis (PEGS) (Fig. 1a, Supplementary Section 1). Briefly, by checking the Pourbaix diagrams we can find the difference in the redox potential (*ΔE*) and/or pH between a soluble guest precursor and a desired guest. We can then shortlist the hosts and reagents (e.g., precursors) that meet the guest formation requirements and select the most appropriate candidates perhaps with other properties (i.e., desired boiling temperature and hydrophobicity) to manage the ship-in-a-bottle synthesis.

One concern for preparing the ship-in-a-bottle systems is the significant amount of guest depositing outside the hosts^4,33^, which creates a strong bias against the discovery of new functionalities in confinement^6^. Efforts to immobilize the precursor and to control the guest formation mostly inside the host include methods such as chemical grafting^33,34^ and electrostatic interactions^35^. These approaches, however, only work for a small portion of hosts with special chemistries (e.g., hosts with functionalizable parts or electrical charge). Enabled by the PEGS method, we may select the desired reagents with functionalities (i.e., temperature-controlled selective desorption and hydrophobic–hydrophilic interaction mentioned in Fig.1 and Supplementary Section 2.4) to control the loading position, and thus mitigate the outer surface deposition issue. Therefore, hosts no longer need to exhibit special chemistries to synthesize the right guests inside them.

The ability to synthesize a large variety of catalytic and optoelectronic materials with an even greater variety of available and synthesizable MOF materials is a combinatorial treasure trove of potential discoveries^2,4,6,16^. We demonstrate the synthesis of RuO_2_ confined within a MOF and then characterize the resulting products. The MOF used is MOF-808-P^36^ [Zr_6_O_5_(OH)_3_(BTC)_2_(HCOO)_5_(H_2_O)_2_, BTC = 1,3,5-benzenetricarboxylate], which is based on {Zr_6_O_8_} clusters (Fig. 1b) with the **spn** topology and has large cavity and aperture diameters (ca. 18 Å and ca. 14 Å, respectively). The MOF is stable in aqueous solution over a wide pH range of 3–10^37^. The synthesis of MOF-808-P is modified from MOF-808 and requires shorter time^36,38^. The good synthetic control of the loaded guests allows us to demonstrate that molecules can behave very differently on the guest surfaces. For example, molecules (such as CO and O_2_) adsorbed on the confined RuO_2_ at low temperatures (e.g., ≤150 °C) can exhibit a drastically different behavior compared with that on porous silica-supported RuO_2_. Most surprisingly, such guest inclusion inside the MOF host via PEGS turns inactive oxide surfaces into highly active catalysts. RuO_2_, which is usually easily deactivated at low temperatures by strong CO adsorption^39–41^, stays highly active in MOF confinement (>97% conversion after 12 h of continuous reaction) for CO oxidation. We believe that by using the PEGS method, many candidates, e.g., oxide, hydroxide and sulfide materials, can be expected to show other unique and surprising behaviors for catalysis and optoelectronics in confinement. In the following parts we describe in more detail the steps in applying the PEGS approach.

## Results

### Rational synthesis of RuO_2_ inside MOF-808-P

In revisiting the Pourbaix (redox potential-pH) diagrams of aqueous (element-H_2_O) systems^27–29^ (e.g., Ru-H_2_O system given in Fig. 1a), we realized that the reverse use of Pourbaix diagrams could guide the formation of insoluble compounds as long as the difference in the redox potentials between the reactants and the pH were chosen to make insoluble cluster formation thermodynamically favorable. For example, oxyanions (A_*x*_O_*y*_^*z*−^) in A-H_2_O (A) systems could form oxides and hydroxides^27–29^, where A is the desired element in the guest. Therefore, the PEGS strategy that we propose (detailed in Supplementary Section 1 and Supplementary Figure 1) can be more flexible and versatile than known methods^2,4^ and suitable for forming a range of insoluble guest compounds under relatively mild conditions inside pre-formed nanoporous hosts, e.g., MOFs and zeolites. As a demonstration, we have synthesized RuO_2_ inside a water-stable Zr-based MOF, MOF-808-P^36^, i.e., RuO_2_@MOF-808-P. We used potassium perruthenate (KRuO_4_) as the RuO_2_ precursor and 2-tert-butyl-4-methylphenol (tBMP, Fig. 1b) lipid as the reducing agent (Fig. 1; details in Supplementary Section 2.1).

According to the PEGS method tutorial detailed in Supplementary Section 1, from the Ru-H_2_O Pourbaix diagram (Fig. 1a) we have seen that at a pH of ca. 8.5 (20 mM aqueous KRuO_4_), the minimum *ΔE*_reduction_ required to form RuO_2_·2H_2_O (the preform of RuO_2_) from the RuO_4_^−^ (aq) domain is ca. 0.3–0.4 V. Therefore, a small reducing reagent which matches this *ΔE*_reduction_ is required. Additionally, to perform the guest loading with the aforementioned position control, we need a reducing reagent that is hydrophobic and has temperature-controlled selective desorption capability (Supplementary Section 2.4). We have chosen the small lipid tBMP (Supplementary Figure 2), which meets the above-mentioned properties and is chemically similar to the well-known antioxidant lipid, butylated hydroxytoluene requiring ca. 0.3 V to be partially oxidized^42,43^. We expect that if it also provides ~0.3 V of oxidation potential, it may be sufficient to reduce RuO_4_^−^ to RuO_2_·2H_2_O within a controlled pH range of 5–10.

We have verified that the MOF-808-P by itself remains white in color (i.e., no color change) in the KRuO_4_ solution, indicating no reaction in the MOF upon placement into the KRuO_4_ (aq) solution in the absence of tBMP. For the reaction, tBMP is first introduced into the MOF using diethyl ether (DE) as the solvent. With the aid of temperature-controlled selective desorption of tBMP and DE (Supplementary Section 2.4, Supplementary Figure 3)^8,44^, tBMP outside the MOF and all the DE was desorbed, while tBMP inside the MOF mostly remained to obtain tBMP@MOF-808-P. After immersing tBMP@MOF-808-P into the KRuO_4_ (aq) solution, the hydrophobic nature of tBMP kept it entrapped and solid products from the reduction of KRuO_4_ were obtained inside the MOF, minimizing the material formation outside the MOF (Fig. 1c). The initial product—hydrated RuO_2_ in the MOF—was further dehydrated to RuO_2_ (i.e., as-synthesized RuO_2_@MOF-808-P) at ca. 140 °C in N_2_^45^. Furthermore, tunable loading amounts of the RuO_2_ guest were achieved by adjusting the mass ratio between tBMP and the MOF (thermogravimetric analysis in Supplementary Figure 4 and N_2_ adsorption measurements in Supplementary Figure 5).

### RuO_2_@MOF-808-P characterizations and loading control

We have confirmed the preservation of the MOF host structure throughout the synthesis of RuO_2_@MOF-808-P by its mostly unaltered powder X-ray diffraction (PXRD) patterns (Supplementary Figure 6). Pore occupation by the guest was revealed by the reduction in pore volume as shown in the pore distributions (Supplementary Figure 5d), which were derived from the N_2_ adsorption measurements. The incorporation of Ru-based guests in the MOF was confirmed with a combination of (i) energy-dispersive X-ray spectroscopy (EDS) element mappings obtained from both scanning electron microscopy (SEM) (i.e., SEM-EDS, Supplementary Figure 7) and scanning transmission electron microscopy (STEM) (i.e., STEM-EDS, Supplementary Figure 8), and (ii) X-ray photoelectron spectroscopy (XPS) (Supplementary Figure 9). The nature of the Ru-based guest was partly revealed from the XPS Ru 3p_3/2_ peak position (Supplementary Figure 10) at ca. 463.2 eV, which matches the standard Ru^4+^ peak^46^. X-ray absorption fine structure measurements (Supplementary Figure 11), using Ru foil and anhydrous RuO_2_ as references, identified the dominant Ru-O vector at ca. 1.78 Å^47^. Furthermore, a dark-field STEM (DF-STEM) image (Supplementary Figure 12) shows particles (ca. 15 Å in diameter) with electron diffraction fringes. The small particle size is consistent with the PXRD results, as no X-ray diffraction peak could be found for very small guest^16^. The space between two adjacent lines in the fringes is 2–2.5 Å, which matches the inter-planar spacing [*d*_(011)/(101)_ or *d*_(200)/(020)_] expected for tetragonal RuO_2_ (space group: P4_2_/mnm). Note that further reduction in adsorbed volume of N_2_ can be explained by partial pore collapse and/or amorphization^24,48,49^. This is supported by the disappearance of PXRD peaks (i.e., less ordered) above 40° for as-prepared RuO_2_@MOF-808-P as compared with dried MOF-808-P (Supplementary Figure 13). No significant potassium (K) residual could be found by inductively coupled plasma-optical emission spectrometry (ICP-OES) in the RuO_2_@MOF-808-P. This is also consistent with the SEM-EDS spectrum (Supplementary Figure 7, no peak at 3.314 keV for Kα) and XPS spectra (Supplementary Figure 9, no peak around 294.0 eV for K 2p).

**Fig. 2:**
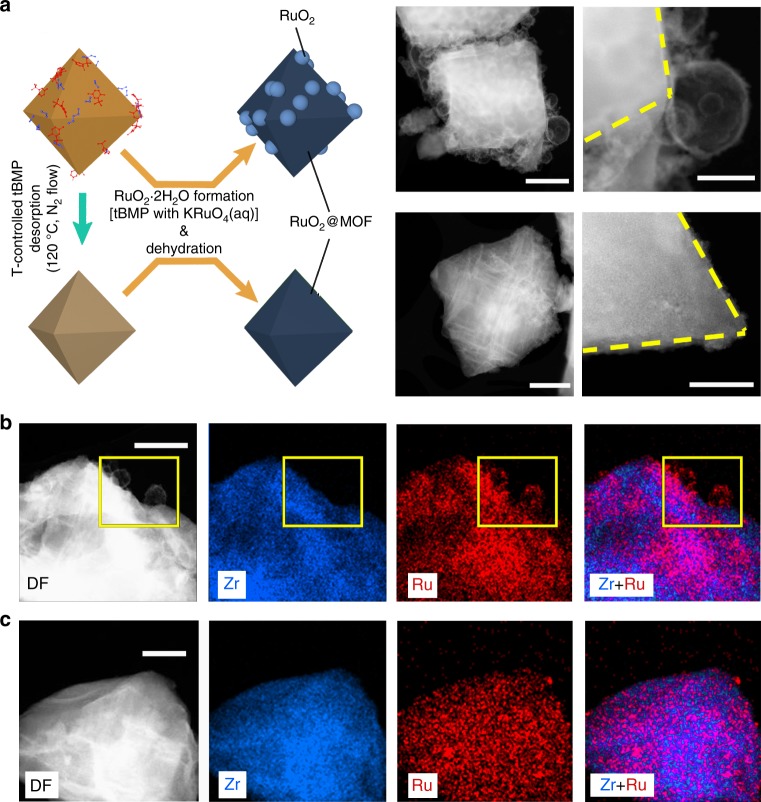
Controllable RuO_2_ guest formation inside (or both inside and outside) MOF-808-P. **a** RuO_2_ can be formed both inside and outside the metal–organic framework (MOF), or only inside the MOF (i.e., RuO_2_@MOF-808-P) via temperature (*T*)-controlled selective desorption of the 2-tert-butyl-4−-methylphenol (tBMP) molecules outside the MOF. Dark-field scanning transmission electron microscopy (DF-STEM) images to the right show spherical shell structures on the outer surface of the MOF crystals (top, for RuO_2_ formed inside and outside the MOF, scale bars: 500 nm and 200 nm for left and right) vs. clean MOF crystal edges (bottom, for RuO_2_ loaded mostly inside the MOF, scale bars: 500 nm and 50 nm for left and right). The controlled deposition was further verified by STEM-energy-dispersive X-ray spectroscopy (EDS) Zr and Ru mappings for **b** RuO_2_ formed inside and outside the MOF, scale bar: 200 nm, and **c** RuO_2_ loaded mostly inside the MOF, scale bar: 100 nm. The yellow frames in (**b**) highlight the Ru-based spherical shell structures. Raw images are provided as a Source Data file

To demonstrate the loading position control, we performed the redox reactions by adding KRuO_4_ (aq) solution to tBMP/DE/MOF-808-P mixture with and without the temperature-controlled selective desorption (Fig. 2a). By deliberately avoiding the temperature-controlled selective desorption, we obtained a significant material deposition on the outer surface of the MOF (Fig. 2a, top) in the dehydrated product. Since the tBMP/DE mixture on the outer surface forms droplets in contact with the KRuO_4_ (aq) solution to minimize the surface energy due to hydrophobic–hydrophilic repulsion, tBMP (outside the MOF) can only react with KRuO_4_ at the droplet-water interface forming a solid shell of hydrated RuO_2_. This is consistent with the spherical shell nanostructures deposited outside the MOF. The chemical composition of the spherical shell structures was verified by STEM-EDS (Fig. 2b). While both Zr and Ru signals are detected from the Zr-based MOF region after RuO_2_ loading, only Ru signal could be collected for the spherical shell nanostructures (highlighted in the yellow frame in Fig. 2b). In contrast, the dehydrated product (i.e., RuO_2_@MOF-808-P) from the reaction between KRuO_4_ (aq) solution and tBMP@MOF-808-P (with the temperature-controlled selective desorption) showed quite a clean MOF surface (Fig. 2a, bottom). Furthermore, the Ru signal mapping overlaps well with that for Zr and the MOF DF-STEM image (Fig. 2c). The significant outer surface deposition is therefore proved to be effectively inhibited by applying both temperature-controlled selective desorption and hydrophobic–hydrophilic repulsion.

### Weakened CO and O interactions

In heterogeneous catalysis both catalyst surface structure and molecule surface adsorption have a significant influence on the catalytic performance^39,50^. We selected CO oxidation, which is relatively simple and well documented for a wide range of metal-based catalysts, as a prototypical reaction to understand the significance of molecule interactions with RuO_2_^39,51–55^. Meanwhile, CO oxidation (i.e., elimination) is practically important for lowering automotive exhaust emissions, producing CO-free hydrogen for fuel cells and ammonia synthesis, and cleaning air, particularly at low temperatures and in humid air^39,52–54^. At low temperatures, the RuO_2_ is often regarded as a poor catalyst for CO oxidation because of surface passivation^39,40^. Below 150 ^o^C, the dominant mechanism for this reaction is the Langmuir–Hinshelwood process^39,40,56^, in which the adsorbed CO combines with dissociated O_2_ species (i.e., O atoms) to produce CO_2_. Strong adsorption of CO and O species on RuO_2_, however, usually results in the formation of densely packed CO and O domains, where the limited surface desorption and diffusion of both species cause the low catalytic activity^39–41^. The PEGS synthesis of RuO_2_@MOF-808-P allows weaker CO and O interactions with RuO_2_ surface as compared to the commonly used porous silica-supported RuO_2_ catalyst (RuO_2_/SiO_2_)^3,17,50,57^, which will be discussed below. We prepared the RuO_2_/SiO_2_ with a conventional impregnation method^58^ and a commercially available amorphous SiO_2_ with mesoporosity (Supplementary Figures 14-16). Both RuO_2_/SiO_2_ and RuO_2_@MOF-808-P samples contained ca. 10 wt% Ru.

**Fig. 3:**
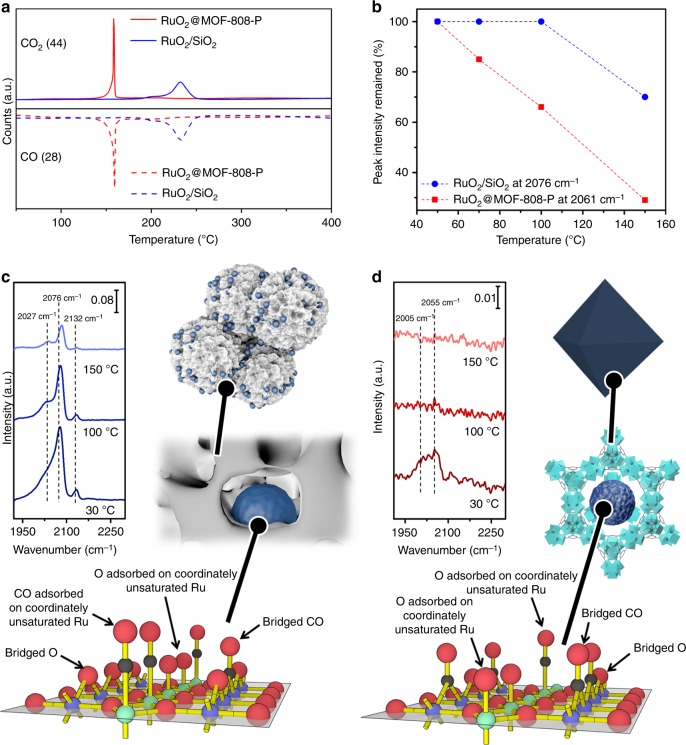
CO and O interactions with RuO_2_ for RuO_2_/SiO_2_ and RuO_2_@MOF-808-P. **a** CO-temperature-programmed reduction (CO-TPR) in flowing CO and **b** temperature-dependent diffuse reflectance infrared Fourier transform spectroscopy (DRIFTS) peak intensity reduction (due to CO desorption) for samples with only surface-adsorbed CO in flowing Ar. DRIFTS results for **c** RuO_2_/SiO_2_ and **d** RuO_2_@MOF-808-P with both surface-adsorbed CO and O in flowing Ar at various temperatures. The RuO_2_ (110) surface was taken as an example to assist our interpretation of the DRIFTS results in Table 1 (O in red, C in black, and green and blue for alternating rows of Ru with different {RuO_6_} octahedral orientation). Source data are provided as a Source Data file

Ru-O interactions within the RuO_2_ nanostructures were tested by CO-temperature-programmed reduction (CO-TPR), which was performed with pre-oxidized samples equilibrated in flowing CO, and then gradually heated to find the minimum temperature where the lattice Ru could be reduced (Fig. 3a). The reduction peak for RuO_2_@MOF-808-P is much sharper and at a much lower temperature (~160 °C) than that from RuO_2_/SiO_2_ (~240 °C). The result was further confirmed by in situ X-ray absorption near edge structure (XANES) spectra, which showed that RuO_2_@MOF-808-P was reduced more significantly than RuO_2_/SiO_2_ by 5% CO at 30 ^o^C (Supplementary Figure 17). The high reducibility of RuO_2_ (i.e., weaker Ru-O bonding) within the MOF is likely the result of an electronic confinement effect, which causes bonding orbital distortion^16^. Accordingly, we deduce that the interaction of O with the RuO_2_ surface in RuO_2_@MOF-808-P was significantly weakened.

**Table 1:** Diffuse reflectance infrared Fourier transform spectroscopy (DRIFTS) absorption bands for RuO_2_/SiO_2_ and RuO_2_@MOF-808-P and their indications^40,56^

Sample	DRIFTS band (cm^−1^)	CO ads. type	Indication
RuO_2_/SiO_2_	2132	On-top	
	2076	On-top	Presence of densely packed CO domains resisting CO oxidation at low temperatures
	2027	Bridging	
RuO_2_@ MOF-808-P	2055	On-top	Loosely packed state of CO
	2005	Bridging	With even fewer adsorbed O neighbors nearby

The weaker interaction of CO with the MOF-confined RuO_2_ surface was revealed by temperature-dependent diffuse reflectance infrared Fourier transform spectroscopy (DRIFTS) investigations^40,56^ (Fig. 3b–d). For temperature-dependent CO desorption characterization (Fig. 3b), samples were pre-treated in 5 vol% CO with 95 vol% He gas at room temperature and then heated up to 150 °C in flowing Ar. The on-top CO molecules (CO absorbed on coordinately unsaturated Ru) at the RuO_2_@MOF-808-P surface were lost from the surface above room temperature, and at 150 °C the main peak at 2061 cm^−1^ almost disappeared (Fig. 3b). In contrast, for RuO_2_/SiO_2_ no CO desorption could be observed below 100 ^o^C and 70% of the corresponding peak intensity (2076 cm^−1^, Fig. 3b) remains at 150 ^o^C.

Under reaction conditions close to room temperature (ca. 30 °C), DRIFTS bands also reveal the packing state of the adsorbed species, with densely packed CO adsorption domains observed on RuO_2_/SiO_2_ but not on RuO_2_@MOF-808-P (Fig. 3c, d). In this experiment, DRIFTS spectra of both samples were collected by adsorbing CO (in 1 vol% CO, 20 vol% O_2_, and 79 vol% He) at room temperature and then heating up in Ar. The DRIFTS bands are summarized in Table 1 with data interpretation supported by previous studies^40,56^. The control experiment on pure MOF material shows no CO adsorption (no similar peak feature found in the MOF-808-P spectra, Supplementary Figure 18). The shift of on-top CO stretching frequency (2076 cm^−1^ for RuO_2_/SiO_2_ versus 2055 cm^−1^ for RuO_2_@MOF-808-P) is attributed to the disappearance of the densely packed CO domains in RuO_2_@MOF-808-P^40,56^. Meanwhile, the weakened interaction of O with RuO_2_ surface, which is suggested by CO-TPR, is also supported by the change of bridging CO frequency (2027 cm^−1^ for RuO_2_/SiO_2_ versus 2005 cm^−1^ for RuO_2_@MOF-808-P) showing fewer O surrounding CO on the surface of the MOF-confined RuO_2_^40,56^.

Overall by confining the RuO_2_ inside the MOF cavity (i) the interactions between O/CO and the catalyst (i.e., RuO_2_) surface are weakened; and (ii) the formation of densely packed CO domains are inhibited. As a consequence, the adsorbed CO is more easily oxidized. This is further reflected by the temperature-dependent DRIFTS results (Fig. 3c, d): surface CO is completely eliminated at 100 ^o^C on the RuO_2_@MOF-808-P catalysts, whereas the majority of CO molecules are still present on RuO_2_/SiO_2_ at 100 °C. The ability to modulate the surface adsorption of CO and O species on RuO_2_ contained in the MOF cavity have motivated us to compare the activities of CO oxidation catalyzed by RuO_2_@MOF-808-P and RuO_2_/SiO_2_, respectively^50,51,57,59,60^.

### RuO_2_@MOF-808-P as a low-temperature CO oxidation catalyst

**Fig. 4:**
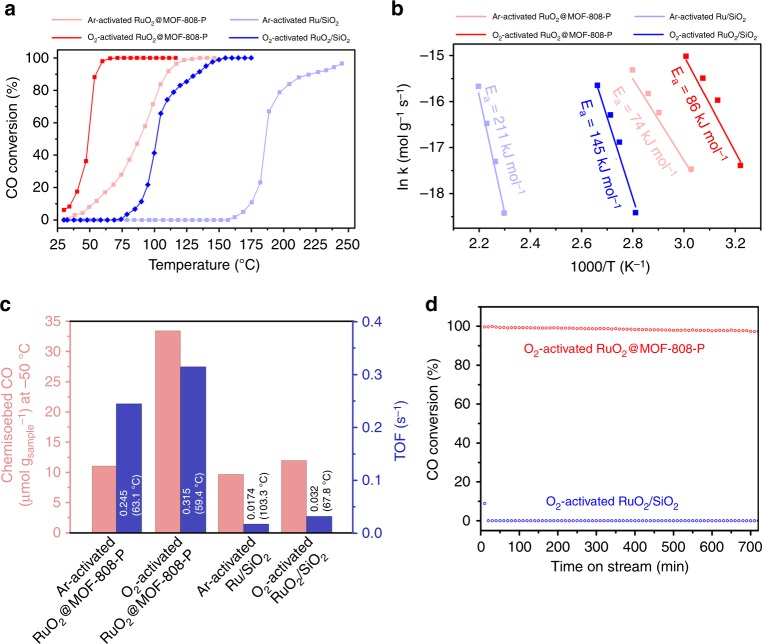
CO oxidation performance over RuO_2_/SiO_2_ and RuO_2_@MOF-808-P catalysts. **a** CO conversion profiles at weight hourly space velocity (WHSV) of 2000 L g_Ru_^−1^ h^−1^ with 15 mg catalysts. **b** Arrhenius plots and calculated apparent activation energies (*E*_a_). **c** Chemisorbed CO at −50 °C (to prevent CO_2_ formation during the measurements) and calculated turnover frequency (TOF, conversion per unit site per unit time). **d** Stability test using O_2_-activated RuO_2_/SiO_2_ and RuO_2_@MOF-808-P catalysts (2000 L g_Ru_^−1^ h^−1^, 15 mg catalysts) at 100 °C. Experimental details are given in Supplementary Section 4.2. Source data are provided as a Source Data file

Under all reaction conditions shown in Fig. 4, the RuO_2_@MOF-808-P catalysts demonstrate superior performance compared with the RuO_2_/SiO_2_ catalysts (ca. 5% vs. no CO conversion at 30 °C; 100% at 65 °C vs. 100% at 150 °C). Meanwhile, both catalysts achieve better CO conversions at low temperature after activation in O_2_ compared with activation in Ar (Fig. 4a), suggesting that oxygen-rich Ru oxide is the active surface structure for low-temperature CO oxidation^61^. From the CO conversion data we calculate the apparent activation energies from the MOF-confined and SiO_2_-supported RuO_2_ to be *E*_a_ = 86 kJ mol^−1^ and *E*_a_ = 145 kJ mol^−1^, respectively, with the MOF-confined catalyst activation energy at the low end of the measured RuO_2_ activation energies (Fig. 4b)^39^. The remarkably higher turnover frequency (TOF) for RuO_2_@MOF-808-P (Fig. 4c) than that for RuO_2_/SiO_2_ and those shown in Supplementary Table 1^62^ is also likely the result of the presence of loosely packed CO molecules. As controls, we have verified that MOF-808-P and tBMP@MOF-808-P are inactive for CO oxidation (Supplementary Figure 19). We can also exclude any significant contribution from the precursor (i.e., KRuO_4_) to the superior catalytic performance of RuO_2_@MOF-808-P by showing that the CO oxidation performance for RuO_2_/SiO_2_ with RuCl_3_ is better than that for RuO_2_/SiO_2_ with KRuO_4_ (Supplementary Figure 20).

The above results indicate that RuO_2_@MOF-808-P is a unique low-temperature CO oxidation catalyst. At 100 °C and 2000 L g_Ru_^−1^ h^−1^ CO flow rate, it still sustained >97% conversion capability after 12 h, whereas under the same conditions RuO_2_/SiO_2_ deactivated completely within 20 min (Fig. 4d). This is consistent with our CO-TPR and DRIFTS results (Fig. 3). We suggest that, for the RuO_2_/SiO_2_ catalysts upon being exposed to the continuously fed reaction gas at low temperatures, the densely packed surface CO and O domains form and prevent the CO–O reaction (Fig. 4c), leading to rapid deactivation at 100 °C (Fig. 4d).

By forming RuO_2_@MOF-808-P using the PEGS strategy, we allow adsorbed CO to react with adsorbed O at low temperature (Fig. 3d) due to the weakened CO and O interactions with the RuO_2_ surface. These modulated interactions can be attributed to the confined microenvironment provided by the MOF^50,51,59^ and/or the unique surface chemistry of RuO_2_ introduced by the PEGS method. Additionally, around 30 °C, we have also observed drastically different CO conversion performances (Supplementary Figures 21 and 22); whereas the RuO_2_/SiO_2_ catalyst is completely deactivated after 12 min, the MOF-confined one still has >40% conversion after 2 h and can be easily re-generated. This further promises normal ambient-condition-based CO removal, in which pure thermal stability is no longer a major concern but potential interactions of the catalysts with water should be considered. In this context, by treating RuO_2_@MOF-808-P with water vapor at 100 °C, we proved that (i) the MOF structure is mostly preserved (Supplementary Figure 23) and (ii) the RuO_2_@MOF-808-P retains its high activity (Supplementary Figure 24), which has been a challenge for recent MOF-based catalyst development^63^.

## Discussion

In summary, we use a preparation of RuO_2_@MOF-808-P as a tutorial to introduce the PEGS strategy, which enables the formation of guests confined in metastable hosts by rational selection of the precursors and conditions for their synthesis. The successful synthesis of RuO_2_@MOF-808-P results in modulated CO/O adsorption behavior and a remarkable improvement in the CO oxidation performance on the RuO_2_ surface at low temperatures. The PEGS method can be extended to other guests and nanoporous hosts with reasonable stability under desired synthesis conditions (Supplementary Figure 25)^24,64^. In theory, the PEGS approach is applicable to metals, oxides, hydroxides and sulfides^65^ as long as their relevant Pourbaix diagrams indicate the feasibility of their formation. So far, we have attempted oxides (i.e., RuO_2_ and MnO_*x*_) with different MOFs (MOF-808-P and DUT-67^66^) and a zeolite Y^20^ (Supplementary Figure 25), and Pd metal particles with MOF-808-P (Supplementary Figures 26–28). Furthermore, benefiting from the recent development of the materials genome approach and the continuous expansion of available databases of Pourbaix diagrams or related phase diagrams (e.g., Materials Project^67–70^), it may even be possible to design guests with more complicated chemistries (e.g., nitrides, phosphides and multi-element compounds). Additionally, considering parameters determining the reactivity in other solvents, diagrams similar to Pourbaix diagrams may be constructed for water-free synthesis. The functions of such guests are not limited solely to catalysis, but could be used to produce a wide variety of optoelectronic materials^2,18^. We believe that this rational synthesis approach to guest functionality in MOF hosts will become a general tool for the systematic synthesis of homologous series of guests confined in porous hosts, as well as a route for combinatorial discovery of materials towards novel practical significance.

## Methods

### Sample preparation

Detailed experimental methods can be found in the Supplementary Information. The considerations to plan a guest synthesis are mentioned in the Supplementary Information 1 and 2.1. To prepare the RuO_2_@MOF-808-P, briefly, MOF-808-P was produced first using a method based on a previously reported synthesis (Supplementary Section 2.3)^36^. The dried MOF-808-P was loaded with tBMP-in-DE solution (50 mg tBMP with 1 ml DE, detailed in Supplementary Section 2.4). The tBMP-to-MOF-808-P mass ratio in the mixture was adjusted to control the final loading of RuO_2_ (Supplementary Figure 5a). The as-prepared tBMP/DE@MOF-808-P powder was then heated at 120 ± 5 °C under N_2_ flow for ca. 1 h (i.e., temperature-controlled selective desorption) to remove the tBMP outside the MOF and DE (Supplementary Section 2.4, Supplementary Figure 3). The treated material was immersed in an excess amount of KRuO_4_ aqueous solution (20 mM) for ca. 4 h to form hydrous RuO_2_@MOF-808-P. It was finally collected by filtration and dehydrated at ca. 140 °C to give as-synthesized RuO_2_@MOF-808-P (Supplementary Section 2.5). Methods for RuO_2_/SiO_2_ preparation and characterizations are given in Supplementary Section 3.1.

### Material characterization

The methods for RuO_2_@MOF-808-P characterizations are given in Supplementary Section 2.6.

### Surface adsorption and CO oxidation investigations

The methods for surface adsorption and CO oxidation investigations are given in Supplementary Section 4.1.

## Supplementary information

Supplementary Information

Peer Review File

Description of Additional Supplementary Files

Supplementary Data 1

Supplementary Data 2

## Data Availability

The authors declare that all data supporting the findings of this study are included in the paper and its supplementary information files, and are available on request from the corresponding authors. The raw images and/or source data underlying Figs. 2–4 and Supplementary Figures. 3–25, 27 and 28 are provided as a Source Data fileset, which is also available in figshare (10.6084/m9.figshare.7588250).
