# Supplementary material for: Rational approach to guest confinement inside MOF cavities for low-temperature catalysis
Source: Nat Commun. 2019 Mar 22;10:1340. doi: 10.1038/s41467-019-08972-x (PMC6430784; doi:10.1038/s41467-019-08972-x)
Supplement: Supplementary file 1 — Supplementary Information [file 41467_2019_8972_MOESM1_ESM.pdf]

Rational Approach to Guest Confinement inside MOF Cavities for Low-Temperature Catalysis

Wang et al.

Supplementary Information for "Rational Approach to Guest Confinement inside MOF Cavities for Low-Temperature Catalysis"

Tiesheng Wang†[1,2,3], Lijun Gao†[4], Jingwei Hou[1,5], Servann J. A. Herou[6,7,8], James T. Griffiths[1], Weiwei Li[1], Jinhu Dong[4], Song Gao[5], Maria-Magdalena Titirici[6,7,8], R. Vasant Kumar[1], Anthony K. Cheetham[1,9], Xinhe Bao[4], Qiang Fu[4]* and Stoyan K. Smoukov[1,6,7,10]*

†These authors contributed equally to this work.

[1] Department of Materials Science and Metallurgy, University of Cambridge, Cambridge CB3 0FS, United Kingdom

[2] EPSRC Centre for Doctoral Training in Sensor Technologies and Applications, University of Cambridge, Cambridge CB3 0AS, United Kingdom

[3] School of Chemistry, The University of Sydney, New South Wales 2006, Australia

[4] State Key Laboratory of Catalysis, iChEM, Dalian Institute of Chemical Physics, Chinese Academy of Sciences, Dalian 116023, People's Republic of China

[5] UNESCO Centre for Membrane Science and Technology, School of Chemical Engineering, The University of New South Wales, New South Wales 2052, Australia

[6] School of Engineering and Materials Science, Queen Mary University of London, London E1 4NS, United Kingdom

[7] Materials Research Institute, Queen Mary University of London, London E1 4NS, United Kingdom

[8] Department of Chemical Engineering, Imperial College London, London SW7 2AZ, United Kingdom

[9] Department of Materials Science and Engineering, National University of Singapore, 117574, Singapore

[10] Department of Chemical and Pharmaceutical Engineering, Faculty of Chemistry and Pharmacy, Sofia University, Sofia 1164, Bulgaria

Corresponding authors:

*Stoyan K. Smoukov, Email: s.smoukov@qmul.ac.uk

*Qiang Fu, Email: qfu@dicp.ac.cn

Contents

1 Guest@Nanoporous-Host System Design Principle: Pourbaix Enabled Guest Synthesis (PEGS) S3

2 Experimental and Characterization for RuO_2@MOF-808-P S7

2.1 Rational Design of RuO_2@MOF-808-P S7

2.2 Materials S8

2.3 MOF-808-P Preparation S8

2.4 tBMP Impregnation and Temperature-Controlled Selective Desorption S9

2.5 RuO_2 Formation Inside the MOF-808-P S11

2.6 Characterization Methods for RuO_2@MOF-808-P S13

2.7 RuO_2@MOF-808-P Characterizations S15

3 RuO_2/SiO_2 Characterizations S19

3.1 Experimental for RuO_2/SiO_2 ..... S19

Sample preparation S19

Materials Characterization Methods S19

3.2 Characterization Results S20

4 Experimental and Supporting Results for Surface Adsorption and CO Oxidation S21

4.1 General Characterization Methods in This Section S21

4.2 CO Oxidation Tests S23

4.3 Supporting Results S24

5 Preliminary Results for Other Guest@Nanoporous-Host Systems Achieved Using Pourbaix Enabled Guest Synthesis (PEGS) S29

References S32

1 Guest@Nanoporous-Host System Design Principle: Pourbaix Enabled Guest Synthesis (PEGS)

Encapsulation of guest moities in the nano-cavities (a.k.a. ship-in-a-bottle) provided by microporous materials (e.g. metal-organic framework (MOF) and zeolite) is an effective approach in catalysis to restrict the growth of nano-entities and prevent them from coalescence.[1–7] Taking Ru-based guest@nanoporous-host as an example, since early 1990s, numerous combinations have been achieved, such as

- Ru@Zeolite-Y[8]
- Ru@MIL-101[9]
- perruthenate@MCM-41 (a mesoporous silica)[10]
- RuO_2@ faujasite zeolite[11]
- Ru-organic complex@MOF[12–14].

Meanwhile, there are several metal@MOF systems used for CO oxidation tests, such as

- Au@ZIF-8[15]
- Pt@UiO-66[16]
- Co_3O_4@ ZIF-8[17]
- Pd@[Ce(BTC)(H_2O)]·DMF[18] (where BTC is benzene-1,3,5-tricarboxylate and DMF is dimethylformamide).

The most promising method to prepare a ship-in-bottle system is to load metal salts and organometallic precursors into pre-formed open-porous framework via solution-based, gas-phase or mechanical-mixing impregnation followed by either thermal/irradiation decomposition or redox reaction with strong reducing reagents like hydrazine, $NaBH_4$ or H_2.[1–7] Common issue for preparing the ship-in-bottle systems, however, is the poor control in growing the nano-entities within the microporous hosts. In most cases along the post-assembly incorporation route, guest moieties are significantly deposited on the outer surface of the porous material.

By revisiting Pourbaix diagrams (redox potential-pH) of various systems [e.g. aqueous (element-H_2O) and element-H_2S][19,20], we realize that insoluble guests can be made from their soluble precursors [e.g. oxyanions ($A_xO_y^{z-}$) in element-H_2O system and thiometallate anions ($A_xS_y^{z-}$) in element-H_2S system] as along as factors like redox

potential and pH are adjusted to match the reaction requirements. We term this approach as *Pourbaix Enabled Guest Synthesis* (PEGS).

Therefore, the synthesis strategy to form the guests can be far more flexible and versatile than the above-mentioned conventional methods. Meanwhile, a range of guest compounds can be made in various nanoporous hosts. In order to achieve the designed guest@nanoporous-host four key criteria need to be considered:

(i) Precursors in mobile states (as gas, liquid or solution)

(ii) Precursor is compatible with nanoporous host & nanoporous host is stable throughout the synthesis

(iii) An external stimulus to guest formation: in the case of redox reaction, matched redox potential between the oxidants (e.g. $A_xO_y^{z-}$) and the reducing agents is required.

(iv) Less mobile (i.e. insoluble) guest products.

A summarized flow chart (Supplementary Figure 1 right) is provided to guide design of guest@nanoporous-host systems. Note that the experimentally working concentrations of the precursors can be different from those predicted in Pourbaix diagrams, as the Pourbaix diagrams only consider the thermodynamically favourable states but the actual reaction will also take account of the actual precursor compounds used and the kinetics.

The Pourbaix diagram for Ru-H_2O system is reconstructed based on the previous efforts reported.[19–21] Here, we assume the aqueous concentration of insoluble Ru-containing compound is negligible (effectively 0 M). Since our study is focusing on aqueous systems with pH between 5 and 10, we only consider this pH range to simplify the question. Between pH 5 and pH 10, apart from metallic Ru, several other forms of Ru-containing compounds are thermodynamically favorable to be formed, namely: H_2RuO_5 (solution), RuO_4^- (solution), Ru_2O_5 (insoluble solid), $RuO_2{\cdot}2H_2O$ (insoluble solid) and $Ru(OH)_3{\cdot}H_2O$ (insoluble solid).[21–23]

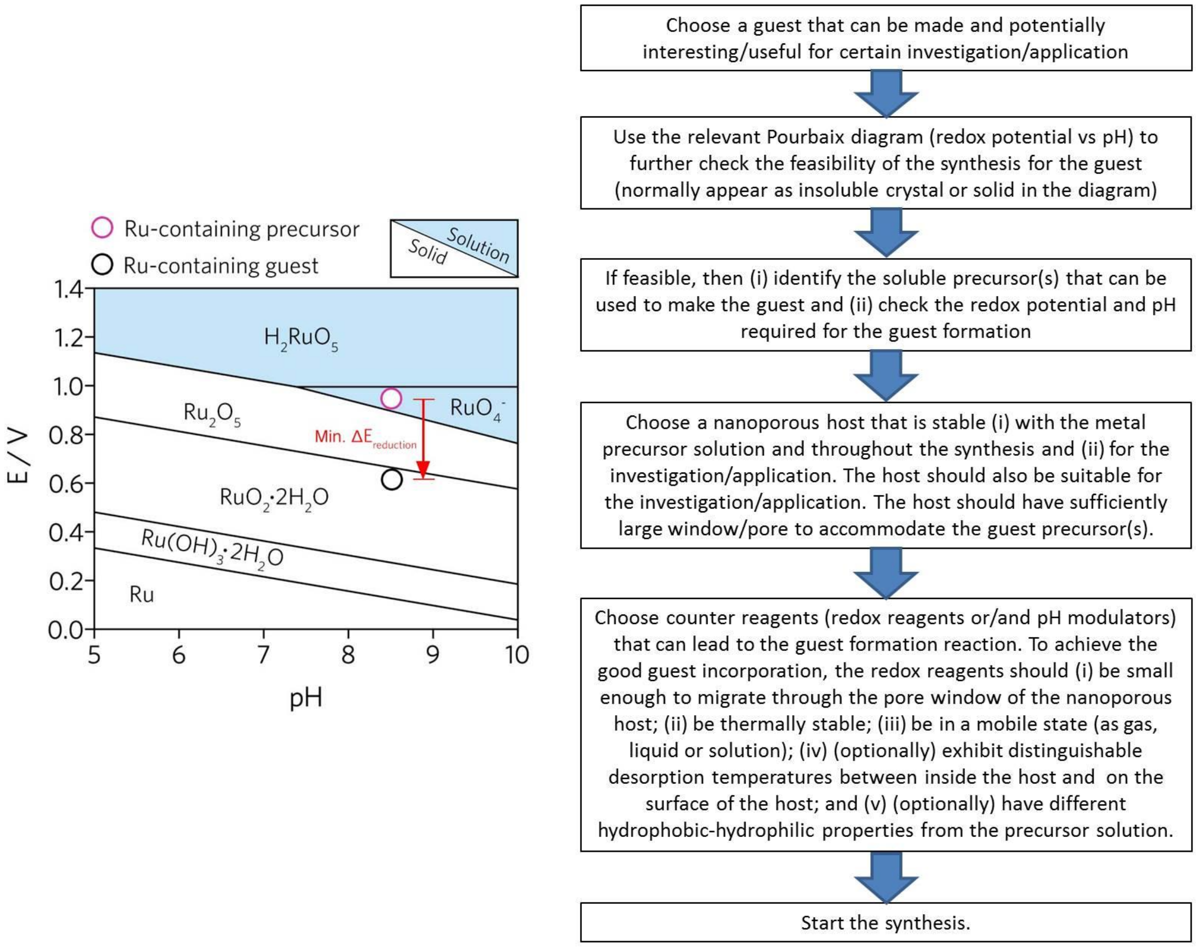

Supplementary Figure 1. A flow chart (right) about steps to go through to form guest inside nanoporous host using PEGS strategy and an example of the Pourbaix diagram for Ru-H_2O system indicating the formation of hydrous RuO_2 from RuO_4^-.[19–21] The concentration of Ru-based solution, C_{Ru}^0, is 20 mM.

Based on the results detailed by Povar and Spinu[21], after corrections and modifications, we can construct the Pourbaix diagram for Ru-H_2O system (with a pH range of 5-10, C_{Ru}^0 = 20 mM) to fit our study. There are 6 different half-cell reduction reactions (with their standard electrode potentials, E^0) that can be involved in our pH range:

$Ru(OH)_3{\cdot}H_2O + 3\ H^+ + 3\ e^- = Ru + 4\ H_2O,\ \ E_1^0 = 0.631$ V   Supplementary Equation 1

$RuO_2{\cdot}2H_2O + H^+ + e^- = Ru(OH)_3{\cdot}H_2O,\ \ E_2^0 = 0.777$ V   Supplementary Equation 2

$Ru_2O_5 + 3\ H_2O + 2\ H^+ + 2\ e^- = 2\ RuO_2{\cdot}2H_2O,\ \ E_3^0 = 1.168$ V   Supplementary Equation 3

$2\ RuO_4^- + 6\ H^+ + 4\ e^- = Ru_2O_5 + 3\ H_2O,\ \ E_4^0 = 1.701$ V   Supplementary Equation 4

$2\ H_2RuO_5 + 6\ H^+ + 6\ e^- = Ru_2O_5 + 5\ H_2O,\ \ E_5^0 = 1.466$ V   Supplementary Equation 5

$H_2RuO_5 + e^- = RuO_4^- + H_2O$, $E_6^0 = 0.996$ V Supplementary Equation 6

According to Nernst Equation for an electrochemical half-cell reduction reaction[24], the electrical potentials, E, can be effectively written as:

$$E = E^0 - \frac{RT}{zF}\ln(\frac{\text{concentration of ions on the right mutiplied together}}{\text{concentration of ions on the left mutiplied together}})$$ Supplementary Equation 7

where, R is the gas constant (ca. 8.314 J·K^{-1}·mol^{-1}), T is the temperature in K, z is the number of electrons transferred in the half-cell reaction and F is the Faraday constant (ca. 96485 C·mol^{-1}).

Furthermore,

$\ln[H^+] \approx 2.303\log[H^+] = -2.303\text{pH}$ Supplementary Equation 8

Therefore, the relationship between E and pH can be established for the Supplementary Equations 1-6 for the given $C_{Ru}{}^0$ ($C_{Ru}{}^0$ = 20 mM in our case) can be established.

$$E_1 = E_1{}^0 + \frac{RT}{F}\ln[\text{H}^+] = E_1{}^0 - 2.303\frac{RT}{F}\text{pH}$$ Supplementary Equation 9

$$E_2 = E_2{}^0 + \frac{RT}{F}\ln[\text{H}^+] = E_2{}^0 - 2.303\frac{RT}{F}\text{pH}$$ Supplementary Equation 10

$$E_3 = E_3{}^0 + \frac{RT}{F}\ln[\text{H}^+] = E_3{}^0 - 2.303\frac{RT}{F}\text{pH}$$ Supplementary Equation 11

$$E_4 = E_4{}^0 + \frac{3RT}{2F}\ln[\text{H}^+] + \frac{RT}{2F}\ln(C_{\text{Ru}}^0) = E_3{}^0 - 3.4545\frac{RT}{F}\text{pH} + \frac{RT}{2F}\ln(C_{\text{Ru}}^0)$$

Supplementary Equation 12

$$E_5 = E_5{}^0 + \frac{RT}{F}\ln[\text{H}^+] + \frac{RT}{3F}\ln(C_{\text{Ru}}^0) = E_5{}^0 - 2.303\frac{RT}{F}\text{pH} + \frac{RT}{3F}\ln(C_{\text{Ru}}^0)$$

Supplementary Equation 13

$$E_6 = E_6{}^0$$ Supplementary Equation 14

A simplified Pourbaix diagram for Ru-H_2O system (with the pH range of 5-10, $C_{Ru}{}^0$ = 20 mM), is then constructed with above-mentioned E vs pH equations. Meanwhile, there is a triple junction in the diagram for a disproportionation reaction:

$6\ RuO_4^- + H_2O + 6\ H^+ = Ru_2O_5 + 4\ H_2RuO_5$ Supplementary Equation 15

2 Experimental and Characterization for RuO_2@MOF-808-P

2.1 Rational Design of RuO_2@MOF-808-P

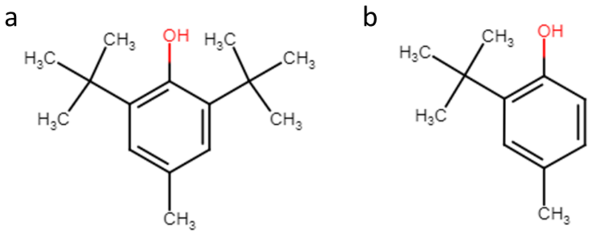

Supplementary Figure 2. (a) butylated hydroxytoluene (BHT) and (b) 2-tert-butyl-4-methylphenol (tBMP).

As a demonstration, we synthesized RuO_2 inside a MOF. According to the Ru-based Pourbaix diagram (Supplementary Figure 1 left)[20], we used a perruthenate salt, potassium perruthenate ($KRuO_4$), as the precursor, which can form hydrous RuO_2 via reduction reaction (Supplementary Figure 1 left). We selected a water-stable MOF, MOF-808-P[25], as the host, which allows us to use aqueous $KRuO_4$ solution for precursor impregnation. Since the minimum $\Delta E_{reduction}$ required to form $RuO_2 \cdot 2H_2O$ (the preform of RuO_2) from the RuO_4^- (aq) domain is ca. 0.3-0.4 V, we need a reducing agent which can match this potential to trigger the reaction. For the reducing agent, we chose 2-tert-butyl-4-methylphenol (tBMP), an analogy to a well-known antioxidant lipid (i.e. hydrophobic) in food, butylated hydroxytoluene (BHT)[26,27], which requires ca. 0.3 V to be partially oxidized[26,27]. tBMP is also hydrophobic (i.e. immiscible with water). Unlike the conventional route to achieve metal@MOF which always introduce precursor first[1,2,6], we loaded a diethyl ether (DE) solution of tBMP first so that we can use a temperature-controlled selective desorption method (detailed in SI section 2.4)[28,29] to remove all of DE and tBMP on the outer surface of the MOF (Supplementary Figure 3, further below). Since tBMP is hydrophobic, the tBMP inside stays due to the hydrophobic-hydrophilic confinement created by subsequent $KRuO_4$ (aq) solution impregnation and react with $KRuO_4$. As a consequence, hydrous RuO_2 clusters/particles produced are entrapped inside the MOF (Fig. 1c). The product was washed with water and ethanol. It was then dehydrated at ca. 140 °C under nitrogen to achieve the as-synthesized RuO_2@MOF-808-P. Meanwhile, the relative loading amount of RuO_2 can be tuned by controlling the mass ratio of $KRuO_4$ to MOF-808-P and confirmed by nitrogen adsorption measurements and inductively coupled plasma optical emission spectrometry (ICP-OES) (Supplementary Figure 5, further below). More details about the experimental procedures are provided below.

2.2 Materials

The following chemicals/items were used as received. 1,3,5-benzenetricarboxylic acid (H_3BTC, ACROS Organics™, 98%), zirconyl chloride octahydrate ($ZrOCl_2 \cdot 8H_2O$, ACROS Organics™, 98+%), DMF (Fisher Scientific, 99.7+%, HPLC), formic acid (HCOOH, Fisher Scientific, 98+%), ethanol absolute (C_2H_5OH, Fisher Scientific, 99.5+%, HPLC), Milli-Q water (17 MΩ), tBMP (ACROS Organics™, 99%), diethyl ether (DE, ACROS Organics™, 99+%, ACS reagent, anhydrous), $KRuO_4$ (Alfa Aesar, 97%), $KMnO_4$ (ACROS Organics™, 99+%) and Whatman® polyamide membrane filters (pore size ~0.2 μm), anhydrous $ZrCl_4$ (Sigma-Aldrich, 99.99%), 2,5-thiophenedicarboxylic acid (H_2tdc, Alfa Aesar, 97%), 1-methyl-2-pyrrolidinone (NMP, Alfa Aesar, 99+%), and zeolite Y (Alfa Aesar, Si:Al = 80:1).

2.3 MOF-808-P Preparation

MOF-808-P was synthesized based on the MOF reported by Yaghi et al.[30] The detailed protocol can be find in ref. 25. Briefly, 0.467 g H_3BTC and 2.16 g $ZrOCl_2 \cdot 8H_2O$ were dissolved in a DMF/HCOOH solvent (100 ml DMF with 100 ml HCOOH) first. The solution was kept at 130 °C for 48 h. The formed MOF particles were collected, washed with DMF for three times and kept in Milli-Q water for 3 days. The washed MOF particles (slurry-like) were recollected by filtration and dried gently at 50 °C to remove majority of water. It was further dried at 150 °C under dry nitrogen flow for ca. 3 h. The MOF structure remains after drying shown in Powder x-ray diffraction (PXRD) (Supplementary Figure 6).

2.4 tBMP Impregnation and Temperature-Controlled Selective Desorption

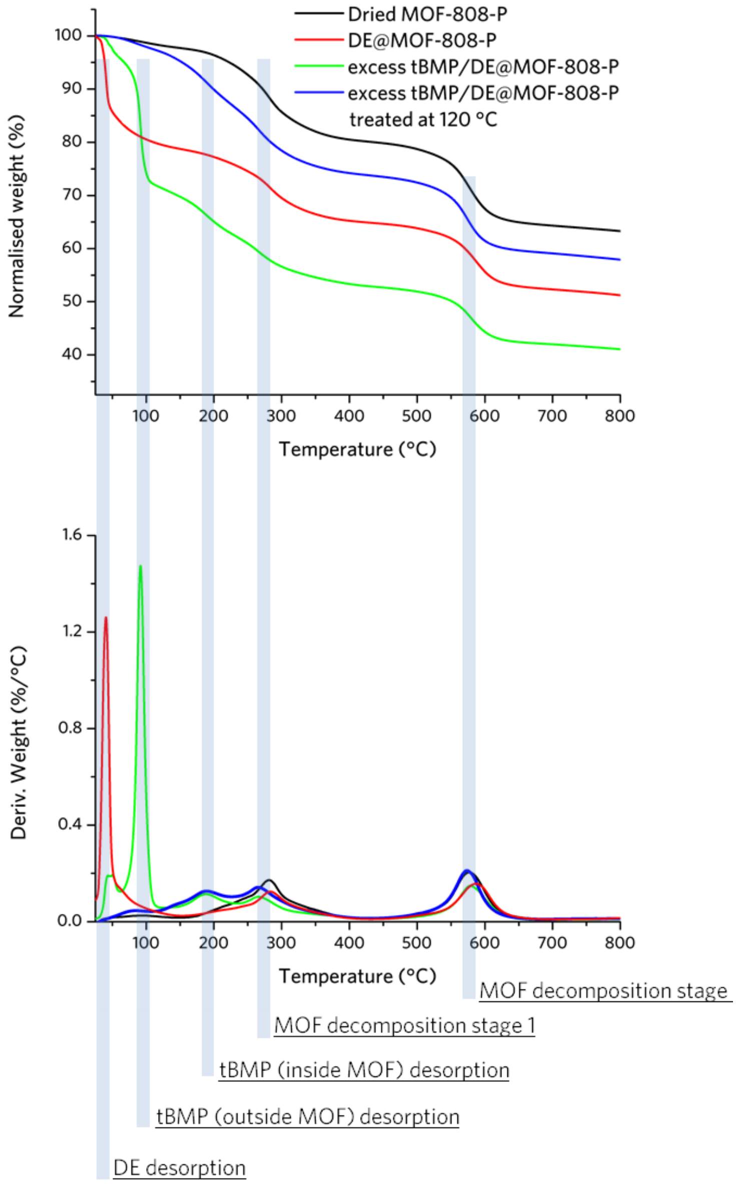

Supplementary Figure 3. HR-TGA on dried MOF, DE@MOF, tBMP/DE@MOF (tBMP in excess) and tBMP/DE@MOF (tBMP in excess) after being treated at ca. 120 °C: normalized weight versus temperature (top) & derivative of weight loss (weight loss upon incremental increase in temperature) versus temperature (bottom). The stepwise drops in the top figure and the peaks in the bottom figure can be assigned to a number of desorption/decomposition events. tBMP outside the MOF has lower desorption temperature than tBMP inside the MOF due to the stronger interaction when the molecule is trapped inside the nanoporous host. This is consistent with similar system for preparing polymer@MOF systems previously observed.[28,29] Hence, DE (the volatile solvent for tBMP) and tBMP (outside the MOF) can be mostly removed when treated the as prepared tBMP/DE@MOF-808-P at ca. 120 °C. In this way, only tBMP inside the MOF host can remain after treatment, i.e. tBMP@MOF-808-P. Source data are provided as a Source Data file.

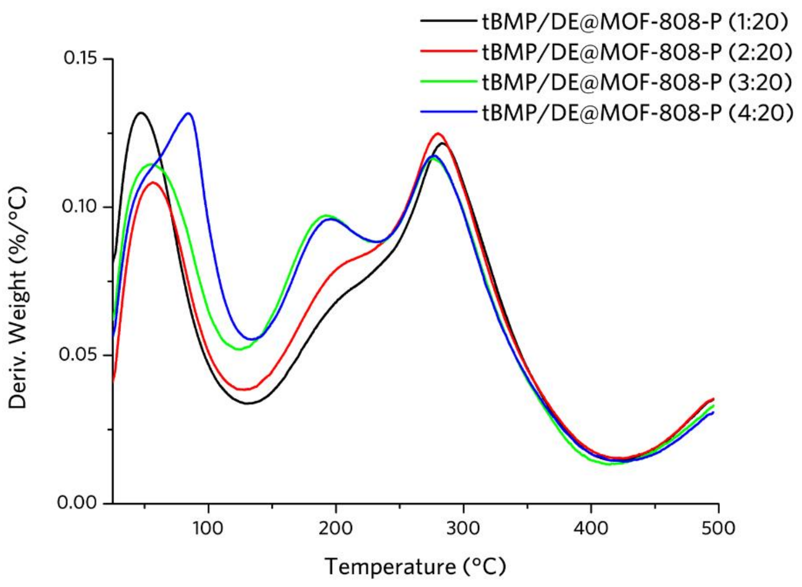

Supplementary Figure 4. HR-TGA results (derivative of weight loss versus temperature) for tBMP/DE@MOF-808-P with different tBMP:MOF-808-P mass ratios. When the tBMP:MOF-808-P mass ratio reaches 4:20 [i.e. tBMP/DE@MOF-808-P(4:20)], the maximum tBMP loading amount inside the MOF has been reached. This is revealed by a distinguishable peak at ca. 100 °C (desorption of tBMP outside the MOF). Source data are provided as a Source Data file.

To impregnate tBMP in MOF-808-P, the MOF was immersed in a tBMP solution (DE as the solvent). The amount of tBMP relative to MOF used is controlled to have a tunable guest loading (Supplementary Figures 4 and 5, further below). As an example, to achieve the sample for CO oxidation (ca. 10 wt% Ru), 50 mg tBMP (in 1000 µl DE) was mixed with 500 mg MOF-808-P. The mixing time is ca. 30 mins. The as-prepared tBMP/DE@MOF-808-P is referred as tBMP/DE@MOF-808-P (2:20), where 2:20 is the tBMP:MOF-808-P mass ratio during tBMP impregnation. The as-prepared tBMP/DE@MOF-808-P was then heated under N_2 flow at 120 °C for ca. 1 h to remove the DE and tBMP outside the MOF. This is the temperature-controlled selective desorption process developed based on the experience by controlled polymer formations inside the MOFs,[28,29] the process is further explained by high-resolution thermogravimetric analysis (HR-TGA) in Supplementary Figure 3. After the treatment, the sample becomes tBMP@MOF-808-P. Samples with different guest loading amounts are prepared to demonstrate the loading tenability (Supplementary Figure 5, further below). Their corresponding HR-TGA results are given in Supplementary Figure 4.

2.5 RuO_2 Formation Inside the MOF-808-P

The as-prepared tBMP@MOF-808-P was collected and reweighed. An excess amount of $KRuO_4$ aqueous solution (20 mM) was then added to tBMP@MOF-808-P. Hydrous RuO_2 forms inside the MOF by mixing tBMP@MOF-808-P with the $KRuO_4$ solution. Since tBMP is immiscible with the aqueous solution, tBMP will be trapped in the MOF during the reaction. Meanwhile, the partially filled MOF host uptakes the $KRuO_4$ solution and accommodates the tBMP-$KRuO_4$ redox reaction within in it.

During the reaction, $KRuO_4$ reduces to RuO_2 while tBMP is oxidized to its oxidizing derivatives similar to the oxidation of BHT.[26,27] The liquid chromatography–mass spectrometry (LC-MS) analysis confirms the presence of ketone derivatives. We kept the reaction for ca. 4 h. The pH of the system was kept within the range of 5-10. In the case of tBMP:MOF-808-P=2:20, the pH value was measured to be ca. 8.5 and ca. 6 before and after the reaction. The as-synthesized hydrous RuO_2@MOF-808-P was collected by filtration (the filtrate remains yellow indicating some $KRuO_4$ left after the reaction) and washed with excess amount of ethanol followed by water. It was then dried at ca. 140 °C[31] for ca. 2 h to become as-synthesized RuO_2@MOF-808-P. After the synthesis, the white MOF-808-P turns to almost black RuO_2@MOF-808-P. Meanwhile, we verified that the MOF-808-P by itself is not reacting with $KRuO_4$, as the MOF-808-P remains white color and no color change in the $KRuO_4$ solution upon mixing. We confirmed that the MOF-808-P is stable throughout the sample preparation based, as there is no significant change in PXRD patterns (Supplementary Figure 6, further below). The as-synthesized RuO_2@MOF-808-P is stable in air and can be stored in ambient condition.

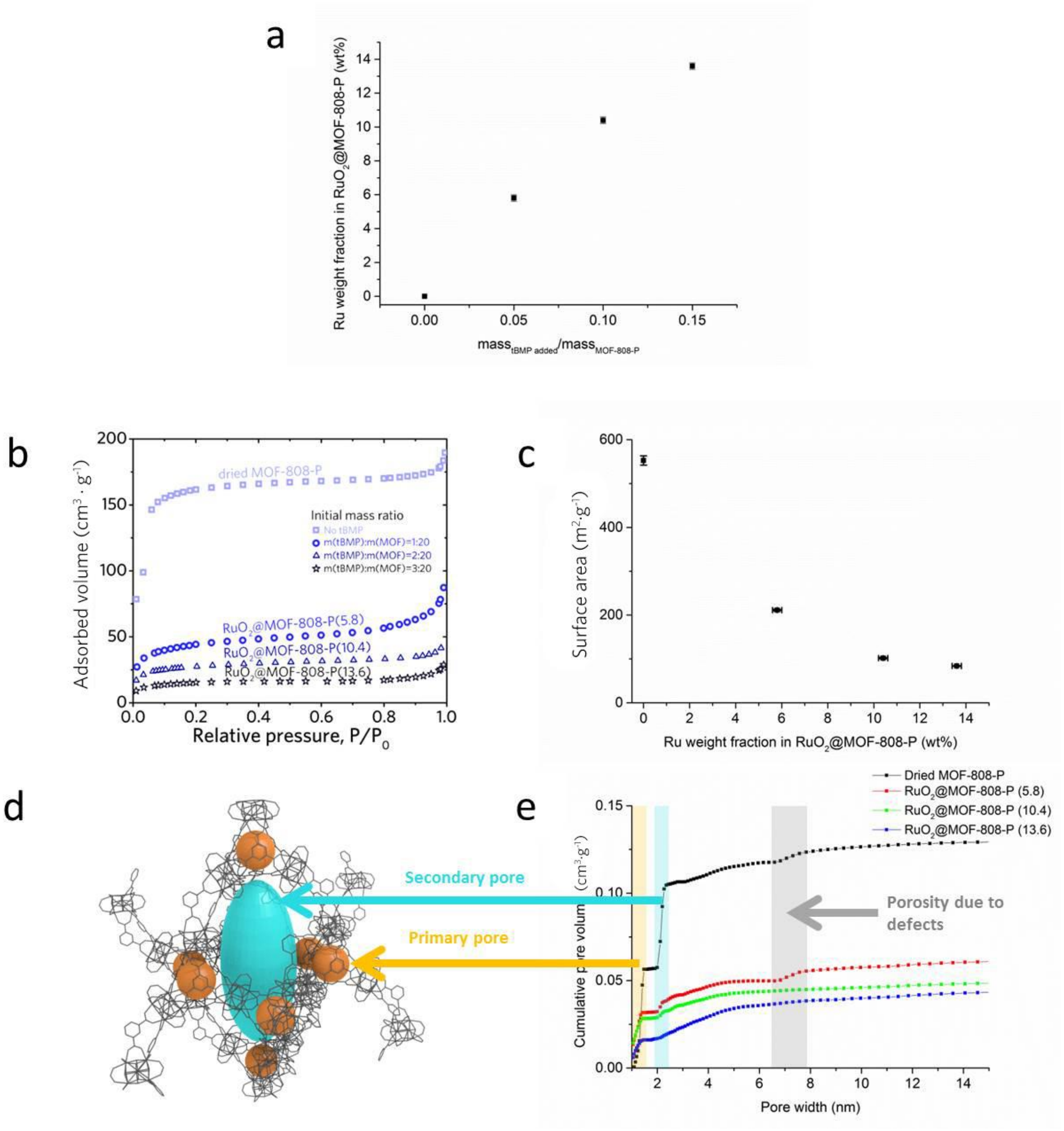

Supplementary Figure 5. Tunable guest loading amount is confirmed by ICP-OES and N_2 adsorption measurements: (a) By varying tBMP amount loading in MOF-808-P [n($KRuO_4$):n(tBMP) kept approx. 2:1], we can achieve different RuO_2 guest loading as revealed from the Ru-element weight fraction measured by ICP-OES. (b) N_2 adsorption isotherms show the amount of guest (i.e. RuO_2) loaded in the MOF is related to the amount of tBMP in the MOF. (c) A N_2-accessible surface area decrease for as-synthesized RuO_2@MOF-808-P (from N_2 adsorption measurements) is observed when more guest (i.e. RuO_2) is incorporated (from ICP-OES). As a consequence, the measured surface area decreases and the measurement pore volume also decreases which is shown in (d) and (e). More specifically, the RuO_2 forms mostly inside the secondary pores due to their larger size. This explains the decrease of the available volume of the secondary pores to the adsorbing gas and therefore a drastic drop of the total pore volume of the material. This observation is also supported by

HR-TEM images of the RuO_2 particles (Supplementary Figure 12). We also noticed that the primary cavity volume also decreases by half due to the partial RuO_2 occupation. The error bars in (a) and (c) represent the standard errors for Ru weight fraction in RuO_2@MOF-808-P from ICP-OES or surface area from N_2 adsorption measurements. The parentheses in samples' labels in (b) and (e) represent the Ru-element weight fraction measured by ICP-OES. Source data are provided as a Source Data file.

2.6 Characterization Methods for RuO_2@MOF-808-P

HR-TGA: TGA was performed with a TA Instruments Q500 thermogravimetric analyzer. Samples were heated from room temperature up to 900 ºC at a rate adjusted based on the mass loss per unit change in temperature (i.e. high-resolution mode) in Ar.

Nitrogen adsorption measurements: The samples in Supplementary Figure 5 were analysed by N_2 adsorption at 77 K using Autosorb and Nova Quantachrome equipment. The samples were degassed at 120 ^{o}C overnight under vacuum. The pore structure and the surface area were calculated by the software Novawin (Quantachrome) using different estimations of the surface (Brunauer Emmett Teller and density function theory). The pore size distribution (PSD) was calculated from the isotherm adsorption line using a quenched-solid model QSDFT assuming slit and cylindrical pores geometries.[27]

PXRD: Powder XRD patterns in Supplementary Figures 6, 13 and 23 were collected on a Bruker D8 ADVANCE with a Ni 0.012 filter between the X-ray source and the sample (2θ from 3.5° to 80° and a step size of 0.04°). Samples were uniformly distributed on a silicon disc supported by a round holder. The holder and the disc were rotated (30 rpm) during the measurement. The illumination area is fixed so that the exposure area forms a circle (16 mm in diameter) with the rotation.

Scanning electron microscopy (SEM) and its associated energy-dispersive x-ray spectroscopy (SEM-EDS): Secondary electron SEM (SE-SEM) images and mappings with energy-dispersive spectroscopy (SEM-EDS) were acquired using a FEI Nova NanoSEM™ with a secondary electron detector and EDS detector (electron acceleration voltage: 15 kV).

Dark-field scanning transmission electron microscopy (DF-STEM), its associated energy-dispersive x-ray spectroscopy (STEM-EDS): DF-STEM images and STEM-EDS mappings in Fig. 2a and Supplementary Figures 8 and 12 were acquired on an FEI Osiris operating at 200 keV fitted with bright field (BF) and annular dark field (ADF) detectors. Energy dispersive spectra were simultaneously recorded on four Bruker silicon drift detectors.

DF-STEM images and STEM-EDS mappings in Figs. 2b and 2c and Supplementary Figure 28 were obtained on a JEOL F200 microscope operated at an accelerating voltage of 200 kV. Energy dispersive spectra were simultaneously recorded on a JEOL EDS detector. STEM samples were prepared by drop-casting 100 μl of sample suspension (ground sample powder dispersed in ethanol) on carbon grids.

X-ray photoelectron spectroscopy (XPS): X-ray photoelectron spectroscopy was undertaken using a monochromatic Al Kα1 x-ray source (*hv* =1486.6 eV) using a SPECS PHOIBOS 150 electron energy analyzer with a total energy resolution of 500 meV. To remove charging effects during the measurements, a low-energy electron flood gun with proper energy was applied. All spectra were aligned to the C 1s at 284.8 eV. For analysis of the Ru $2p_{3/2}$ spectrum, a linear background was subtracted.

Ex situ x-ray absorption spectroscopy (XAS): X-ray absorption spectra measurements were conducted at the BL14W1 beamline of the Shanghai Synchrotron Radiation Facility (SSRF). The spectra at Ru K-edge were recorded in transmission mode. The sample was coated on carbon tape or in plastic sample bag for characterization.

ICP-OES: The metal loadings of Ru in all samples were measured by inductively coupled plasma optical emission spectroscopy (7300DV, Perkin Elmer). The catalysts (5-10 mg) were digested by microwave dissolution in aqua regia and HF solution.

LC-MS: Accurate mass measurements of the BMP oxidization products were performed by coupling an Accela liquid chromatography (LC) system with a Waters 50 mm BEH C18 column interfaced to a Q-Exactive Plus mass spectrometer. For each test, 100 µL of sample was analysed using a 20 min gradient of water (A) versus acetonitrile (B) both with 0.1% formic acid. The mobile phase flow rate was 400 $\mu m \cdot min^{-1}$. After 1 min isocratic conditions at 90 % A, the gradient was operated from 90 % to 5 % A for 10 min., kept at 5 % for another 2 min and then back to the initial conditions in 2 min, which was then kept for another 5 min for the column regeneration. Ionisation was performed in positive and negative polarities for both electrospray and atmospheric pressure ionisation. The nebulized gas flow was 70 $L \cdot h^{-1}$ and drying gas flow was 450 $L \cdot h^{-1}$ at a temperature of 350 °C. Xcalibur v 2.0 software FROM Thermo Scientific was applied for data acquisition and analysis.

2.7 RuO_2@MOF-808-P Characterizations

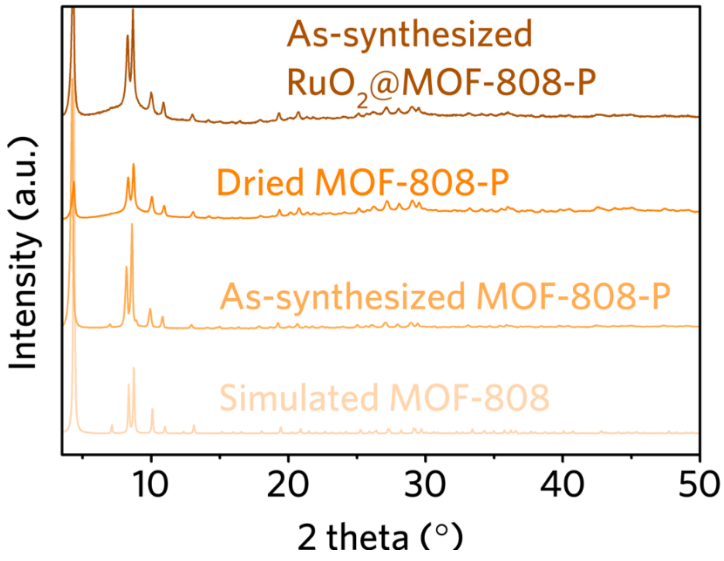

Supplementary Figure 6. PXRD patterns for simulated MOF-808 based on Ref. [25], as-synthesized MOF-808-P, dried MOF-808-P and as-synthesized RuO_2@MOF-808-P. The MOF's structure is mostly preserved after RuO_2 incorporation. No peak for RuO_2 crystal is shown indicating that the RuO_2 particle is very small (< 3 nm) if RuO_2 were there.[15] The PXRD intensity is rescaled for better visualization. Source data are provided as a Source Data file.

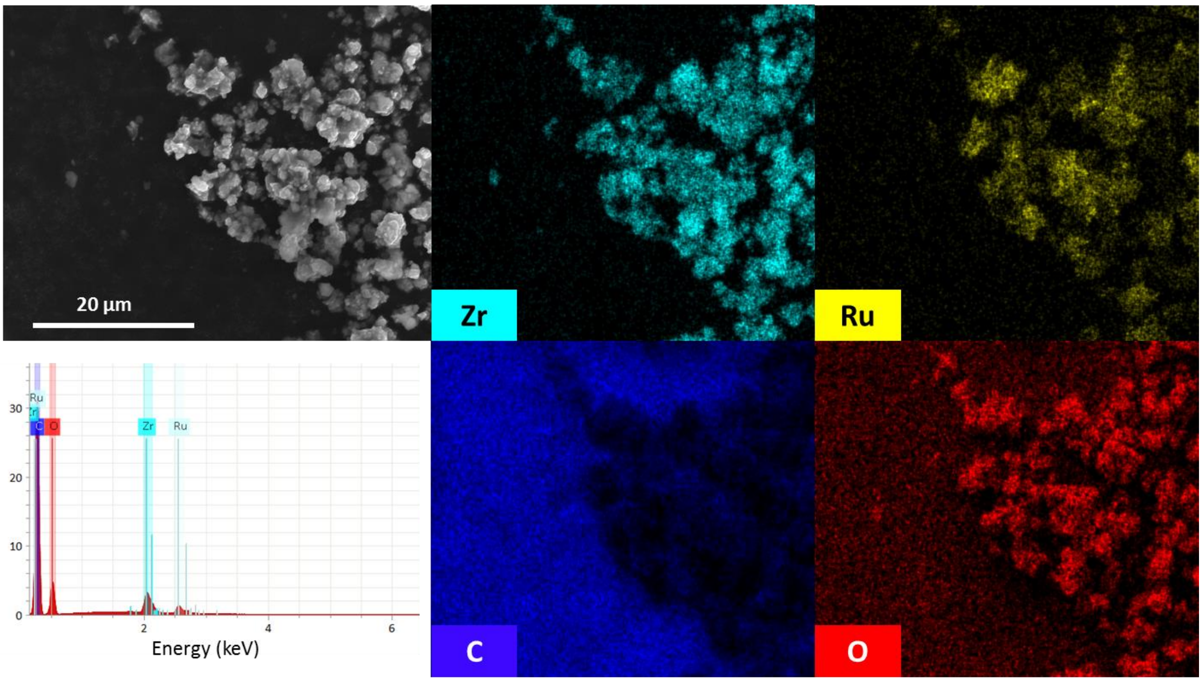

Supplementary Figure 7. SEM-SE image (top left) and SEM-EDS overall spectrum and mappings (Zr, Ru, C and O) for RuO_2@MOF-808-P. The loading of Ru-based guest in a Zr-based MOF host is verified. Strong C signal in background can be found, as the sample powders are immobilized on the

carbon tape. No Kα peak is found at 3.314 keV in the spectrum. Raw images are provided as a Source Data file.

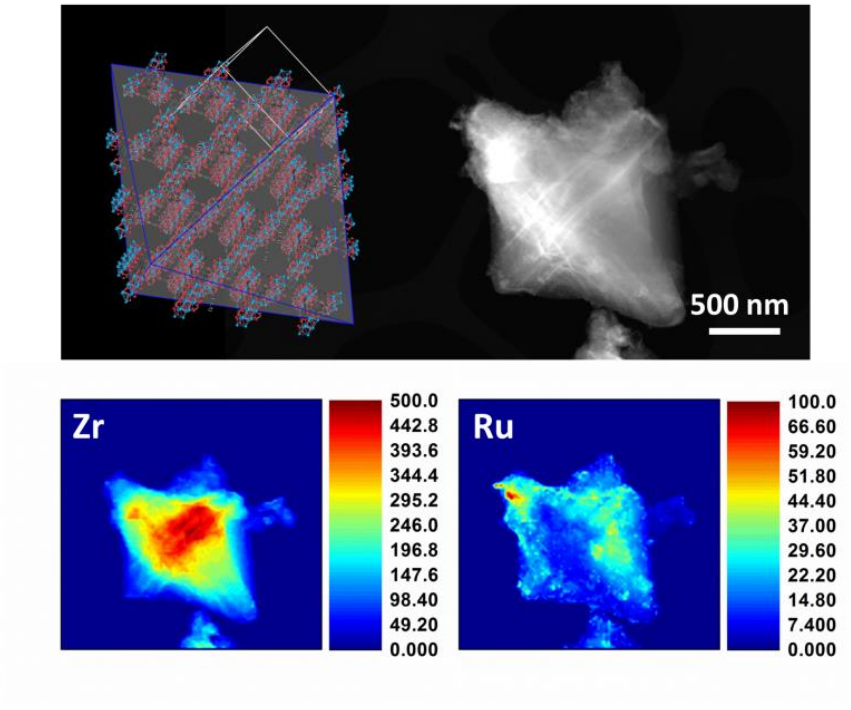

Supplementary Figure 8. Simulated structure and octahedron morphology of MOF-808 based on Ref. 30 by Mercury (https://www.ccdc.cam.ac.uk/solutions/csd-system/components/mercury/) (top left). DF-STEM image (top right) and its associated STEM-EDS mappings (Zr and Ru) for a RuO_2@MOF-808-P particle. Since Zr and Ru have similar atomic number, they interact similarly with the electrons. Therefore, the contrasts from the Zr-based host and Ru-based guest in the DF-STEM image are similar. Relative EDS signal intensity is provided to show the element 2D distribution. Source data and raw images are provided as a Source Data file.

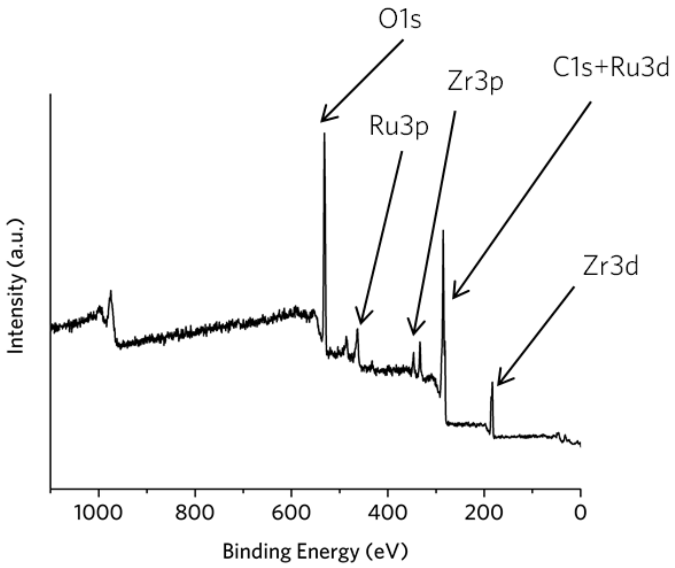

Supplementary Figure 9. XPS survey spectrum for as-synthesized RuO_2@MOF-808-P. The presence of Ru (from the RuO_2 guest), Zr (from the MOF-808-P host), O (from both the guest and the host) and C (mostly from the host) is further verified. No K 2p peak is found between 290 eV and 300 eV. Source data are provided as a Source Data file.

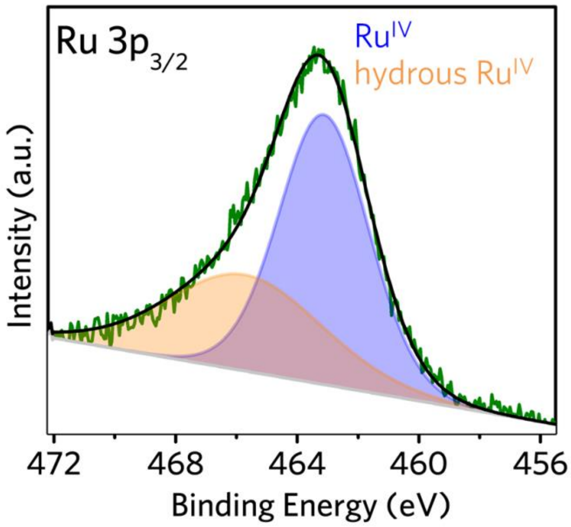

Supplementary Figure 10. XPS Ru $3p_{3/2}$ spectrum for as-synthesized RuO_2@MOF-808-P. The Ru in as-synthesized RuO_2@MOF-808-P is dominated by Ru^{4+} at ca. 463.2 eV.[32,33] We also observe a shoulder at higher binding energy which is likely to be the formation of hydrous RuO_2 guest (in presence of –OH)[32,34] during sample storage or after air exposure. Source data are provided as a Source Data file.

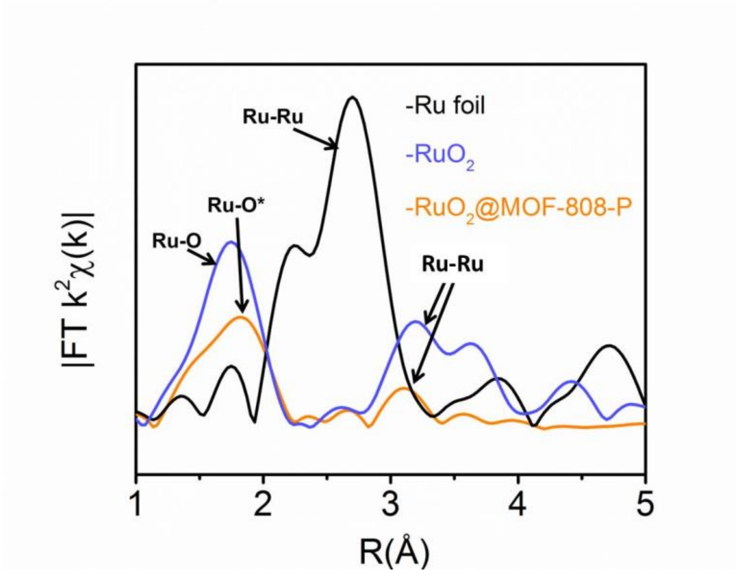

Supplementary Figure 11. Fourier transformed (FT) k^2-weighted $\chi(k)$-function of the x-ray absorption fine structure (XAFS) results for Ru K-edge (ca. 22.1 keV) for Ru foil, anhydrous RuO_2 and as-synthesized RuO_2@MOF-808-P obtained by ex situ XAS. The apparent Ru-O pair can be

identified in RuO_2@MOF-808-P but not Ru-Ru pair as in metallic Ru (i.e. Ru foil). A marginal peak shift can be observed in RuO_2@MOF-808-P compared with reference RuO_2. This may be caused by the presence of C (from the organic ligand of the MOF) in proximity to Ru.[35] Source data are provided as a Source Data file.

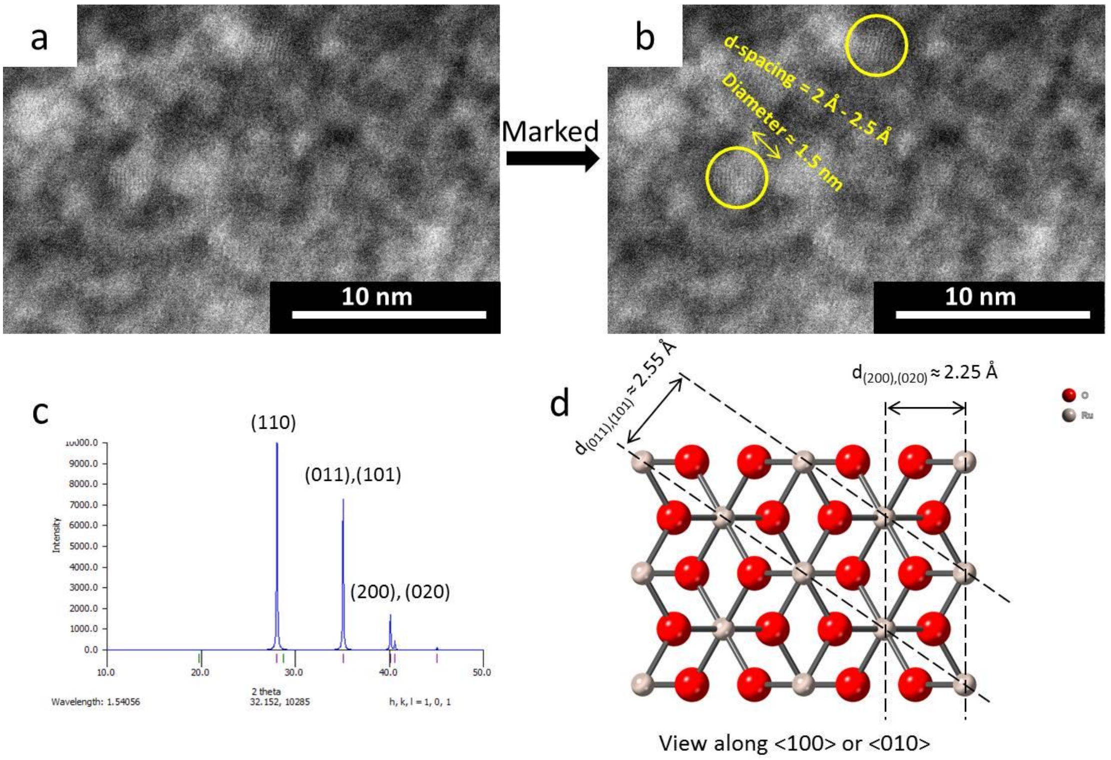

Supplementary Figure 12. (a) DF-STEM image and (b) the same image highlighting the particles (ca. 1.5 nm in diameter) with diffraction fringes of 2-2.5 Å (right). (c) Simulated RuO_2 (space group $P4_2/mnm$) XRD pattern. (d) Two potential diffraction planes in RuO_2 with d-spacing of 2-2.5 Å. The contrasts from the Zr-based host and Ru-based guest are similar, as Zr and Ru interact similarly with the electrons. The presence of small particles is consistent with PXRD patterns in Supplementary Figure 6, as very small particles cannot be revealed by PXRD.[15] Meanwhile, inter-spacing of the diffraction fringes (2-2.5 Å) matches well with $d_{(011),(101)}$ and/or $d_{(200),(020)}$ planes in tetragonal RuO_2 (space group $P4_2/mnm$). Raw images are provided as a Source Data file.

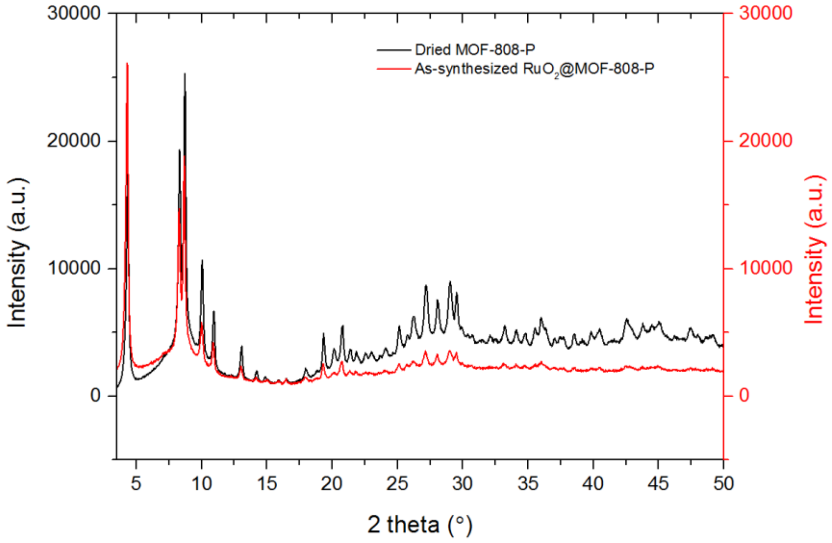

Supplementary Figure 13. More detailed PXRD patterns for dried MOF-808-P and as-synthesized RuO_2@MOF-808-P. The MOF structure is more defective after RuO_2 inclusion (i.e. post-synthetic modification), as PXRD peaks (long-range ordering features) can be hardly observed above 40° (2 theta) for as-prepared RuO_2@MOF-808-P (in red) as compared to dried MOF (in black). Note that the variation of intensity in peak below 5° (2 theta) can be largely influenced by the presence of guest compounds.[36] Source data are provided as a Source Data file.

3 RuO_2/SiO_2 Characterizations

3.1 Experimental for RuO_2/SiO_2

Sample preparation

RuO_2 particles supported on commercial silica (Qingdao Ocean Chemical Company) were prepared by an impregnation method using $RuCl_3$ (Tianjin Kemiou Chemical Reagent, China) as the precursor and $KRuO_4$ (Alfa Aesar Chemical Co., Ltd., China). The nominal loading of Ru in catalysts were maintained at 10 wt%. The fresh catalysts were dried in an oven at 63 ^oC overnight, and then reduced by H_2 (70 ml·min^{-1}) at 250 ^oC for 2 h [donated as Ru/SiO_2 and Ru/SiO_2 ($KRuO_4$)]. Before catalytic activity test, the catalysts were oxidized by O_2 (30 ml·min^{-1}) at 250 ^oC for 1 h (donated as RuO_2/SiO_2). After H_2 reduction, the Ru/SiO_2 ($KRuO_4$) samples were washed by Milli-Q water thoroughly at room temperature to remove K.

Materials Characterization Methods

HRTEM: The TEM images in Supplementary Figure 14 for RuO_2/SiO_2 were acquired on the JEM-2100 microscope operated at an accelerating voltage of 200 kV. TEM samples were

prepared by drop-casting 100 μl of sample suspension (ground sample powder dispersed in ethanol) on copper grids.

Nitrogen adsorption-desorption measurement: The SiO_2 was analysed by N_2 adsorption/desorption at 77 K using Quantachrome Autosorb iQ2 equipment (Supplementary Figure 16). The samples were degassed at 300 ^{o}C for 6 h under vacuum. The surface area was calculated by the Brunauer-Emmett-Teller equation.

PXRD: PXRD patterns in Supplementary Figure 15 for RuO_2/SiO_2 were collected on an Empyrean diffractometer using a Cu Ka (λ = 1.5406 Å) radiation source at 40 kV and 40 mA and scanning rate of $12^{o}\cdot min^{-1}$.

3.2 Characterization Results

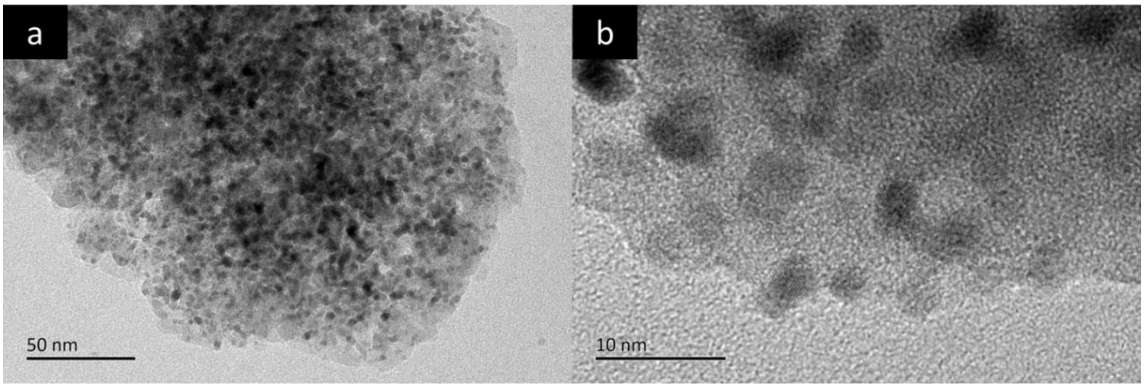

Supplementary Figure 14. HRTEM images about the Ru/SiO_2. The Ru nanoparticles distributed uniformly on the SiO_2 supports with an average diameter between ca. 3 and ca. 5 nm. Raw images are provided as a Source Data file.

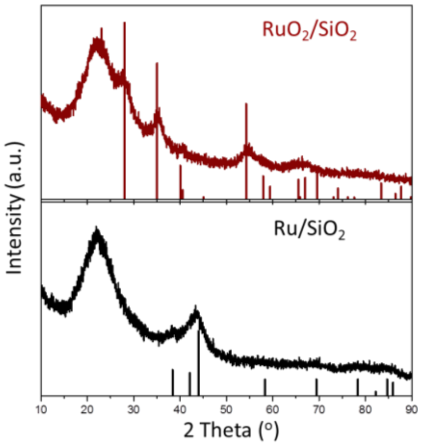

Supplementary Figure 15. PXRD patterns for Ru/SiO_2 (bottom, with simulated Ru peaks provided) and its oxidized form (RuO_2/SiO_2) (top, with simulated RuO_2 peaks provided). The results illustrate

that the metallic Ru was oxidized to RuO_2 after O_2 oxidation at 250 °C. The peak loaded at 23° was attributed to amorphous SiO_2. Source data are provided as a Source Data file.

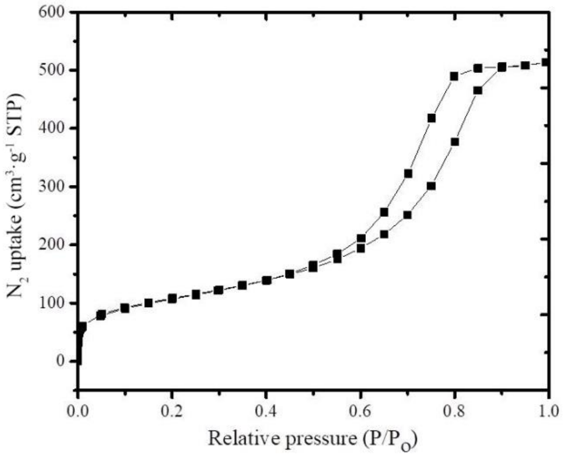

Supplementary Figure 16. Nitrogen physisorption isotherm of the SiO_2 support, which has typical Type IV isotherm shape[37] showing the SiO_2 with mesoporosity with 356.1 $m^2 \cdot g^{-1}$ surface area. Further analysis shows the pore diameter is 5-10 nm. Source data are provided as a Source Data file.

4 Experimental and Supporting Results for Surface Adsorption and CO Oxidation

4.1 General Characterization Methods in This Section

In situ XAS: In-situ XAS measurements were carried out at the BL14W1 beamline of SSRF. The spectra were recorded in transmission mode. Self-supporting pellets were prepared from RuO_2@MOF-808-P and Ru/SiO_2 samples. The pellets were loaded in a quartz cell. A heating element was wrapped around the cell to alter the sample temperature. The temperature was measured by a K-type thermocouple which was in contact with the cell. Prior to the XAS measurements the samples were activated by 20 vol% O_2 with 80 vol% He at 150 °C for 10 min (RuO_2@MOF-808-P) or 250 °C for 1 h (RuO_2/SiO_2) and cooling down to 30 °C in Ar. The spectra were collected for the O_2-activated samples first. The comparison spectra were collected after the O_2-activated samples being treated with flow of 5 vol% CO with 95 vol% He at 30 °C for 30 min.

In situ diffuse reflectance infrared Fourier transform spectroscopy (in-situ DRIFTS): *In situ* DRIFTS spectra were recorded on a BRUKER TENSOR 27 spectrometer equipped with

a diffuse reflectance accessory (the Praying Mantis) and a reaction chamber (operation temperature from -150 °C to 600 °C). The powder sample was loaded into a sample cup. The sample temperature was controlled by a heater and measured by two thermocouples. One of them was placed in the sample cup; the other one was immobilized on the sampling stage. The flow rate passing through the reaction chamber was controlled by the mass flow controllers. The DRIFT spectra were recorded using a spectral resolution of 4 cm^{-1} and accumulating 32 scans. Before the DRIFTS acquisition, the samples were pre-treated in 20 vol% O_2 with 80 vol% Ar at 150 °C for 10 min (RuO_2@MOF-808-P) or 250 °C for 1 h (RuO_2/SiO_2) and cooled down to room temperature in Ar.

For temperature-dependent CO desorption characterization, 5 vol% CO with 95 vol% He was used. The sample was exposed to 5% CO at room temperature first and then decreased to -50 °C by liquid nitrogen and kept for 2 h (RuO_2@MOF-808-P) or 1 h (RuO_2/SiO_2) until no change was observed in the real-time spectra (i.e. CO adsorption in equilibrium). Then the gas flow was switched to Ar gas at room temperature and increased the sample temperature to the targeted one. After each targeted temperature was reached for 10 min, the corresponding DRIFTS spectra were collected.

Under reaction conditions, the O_2-activated samples were exposed to the reaction gas (1 vol% CO, 20 vol% O_2, and 79 vol% He) at room temperature for 30 min first. It was heated to the target temperatures (i.e. 30 °C, 100 °C, and 150 °C) in Ar and held for 10 min, and then the DRIFTS spectra were collected.

CO Temperature-programmed reduction (CO-TPR): The CO-TPR was carried out with a micromeritics chemisorption analyzer (Auto Chem 2910) equipped with a mass spectrometer (MS, Omnistar). The sample (30 mg) was pretreated in 20 vol% O_2 with 80 vol% Ar at 150 ^{o}C for 10 min (RuO_2@MOF-808-P) or 250 ^{o}C for 1 h (RuO_2/SiO_2) and then switched to He gas. After cooling down to 45 ^{o}C in He, the treated sample was exposed to 5 vol% CO with 95 vol% He and held for 30 min. The sample was heated from 45 to 800 ^{o}C with a ramping rate of 10 ^{o}C. The products were analyzed by an on-line mass spectrometry.

CO Pulse Chemisorption: The CO pulse chemisorption was done using a micromeritics chemisorption analyzer (Auto Chem 2920). The sample (30 mg) was pretreated in 20 vol% O_2 with 80 vol% Ar at 150 ^{o}C for 10 min (RuO_2@MOF-808-P) or 250 ^{o}C for 1 h (RuO_2/SiO_2) and then switched to He gas. After cooling down to -50 ^{o}C in He, the treated samples were

exposed to CO pulses consisting of 5 vol% CO balanced with He. All gas follow rate was set to 30 ml·min^{-1}. The CO concentration was measured using a thermal conductivity detector.

4.2 CO Oxidation Tests

The catalysts were loaded into a fixed-bed micro-reactor. Before catalytic activity, the RuO_2@MOF-808-P and Ru/SiO_2 catalysts were exposed to O_2 (O_2-activated) or Ar (Ar-activated) gas with a flow rate of 30 ml·min^{-1} and treated at 150 ^oC for 10 min (to form activated RuO_2@MOF-808-P) and 250 ^oC for 1 h (to form activated RuO_2/SiO_2), respectively. After cooling down to room temperature in Ar gas (30 ml·min^{-1}), the gas stream was switched to a reaction gas (1 vol% CO, 20 vol% O_2, 1vol% N_2, and balanced with He) with a specific weight hourly space velocity (WHSV). The WHSV in Fig. 4a and d is 2000 L·g_{Ru}^{-1}·h^{-1}. For Fig. 4a, the catalytic performance was investigated by temperature-programmed heating with rate of 1 ^oC·min^{-1}. For Fig. 4d and Supplementary Figure 21, the reactions were kept at a specific temperature for 12 h. The gas products were analyzed with an on-line micro-gas chromatograph (Agilent GC-490) equipped with a 5-Å molecular sieve column and a thermal conductivity detector (TCD). For the apparent activation energy measurements, CO oxidation reactions were performed under a kinetic-limiting region in which the CO conversion was below 25% using a much higher WHSV.

For the water stability tests (Supplementary Figure 24) which the catalysts were treated by 100 ^oC water, 30 mg catalysts were loaded at a fixed-bad micro-reactor. Before the activity test, the RuO_2@MOF-808-P catalysts were treated by reaction gas (1 vol% CO and 20 vol% O_2 balanced with He) at 150 ^oC for 10 min. After cooling down to room temperature in Ar gas, the gas flow was switched to reaction gas with WHSV = 400 L·g_{Ru}^{-1}·h^{-1} for activity test. Then the catalysts were treated by 10 vol% water which was injected by a syringe pump (LEAD FLUID, TYD03) at 100 ^oC for 1 h. The lines from the pump to the reactor were heated at a high temperature. After the water treated, the catalysts were exposed to Ar at 120 ^oC for 60 min. The activity test was carried out from 30 ^oC to 100 ^oC with a heating rate of 0.5 ^oC·min^{-1}. The gas products were analyzed by an on-line gas chromatography (Agilent GC 6890) equipped with a packed column PQ200 and a TCD. Before the products analysis, the moisture was condensed by ice.

4.3 Supporting Results

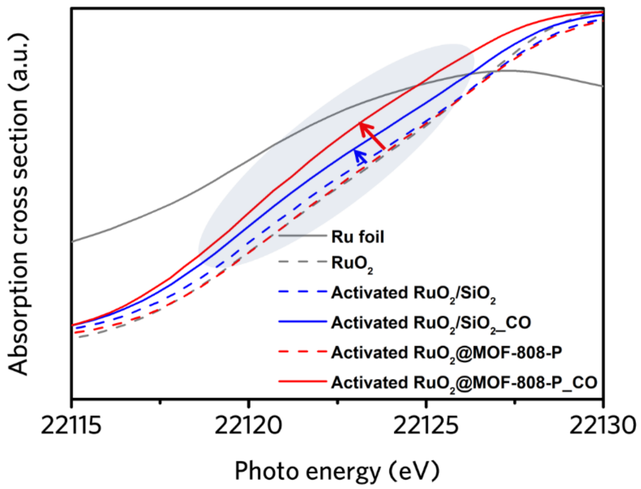

Supplementary Figure 17. In situ x-ray absorption spectroscopy results for RuO_2/SiO_2 (in blue) and RuO_2@MOF-808-P (in red) before and after the CO adsorption at 30 °C (Ru foil and RuO_2 as reference samples). XANES spectra show that both RuO_2/SiO_2 and RuO_2@MOF-808-P are partially reduced upon CO exposure, which is revealed by the change of slope in near-edge region (highlighted in grey). We speculate that surface oxygen atoms in RuO_2 are reacted and replaced by CO at 30 ^{o}C. RuO_2@MOF-808-P, however, is reduced more than RuO_2/SiO_2 upon the same CO exposure condition. The results implied that the Ru-O interaction is weakened by confining RuO_2 in MOF. Source data are provided as a Source Data file.

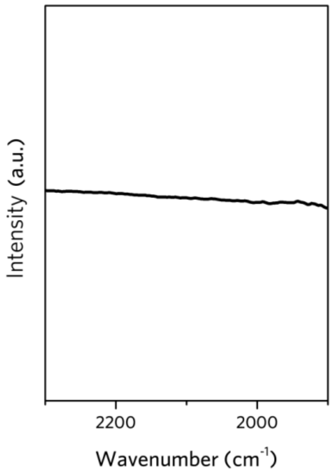

Supplementary Figure 18. In situ DRIFTS spectrum for MOF-808-P treated in the reaction gas and then in Ar at 30 °C. The treatment condition is the same as those mentioned in Fig. 3. There is no peak in the IR spectrum since the MOF-808-P does not adsorb CO under this condition. Source data are provided as a Source Data file.

Supplementary Table 1. Performance comparison for CO oxidation reaction with other guest@MOF systems or Ru-based systems.[38]

catalysts	CO/O_2 ratio	catalysts mass (mg)	WHSV (1×10^4 $ml\cdot g_{cat}^{-1}\cdot h^{-1}$)	particle size (nm)	T of 100 %CO conversion (°C)	T of 50 %CO conversion (°C)	Ea ($kJ\cdot mol^{-1}$)	TOF (s^{-1})	reference
**10 % RuO_2@MOF-808-P**	1/20	15	20	1-2	65	–	86	0.32	This work
**10 % RuO_2/SiO_2**	1/20	15	20	3-5	155	–	145	0.03	This work
**5 % Au@ZIF-8**	1/21	100	6	4.2	225	170	–		15
**15 % Co_3O_4@ZIF-8**	1/20	100	3	16.4	80	58	–		17
**m-5$RuO_2$10CuO/CeO_2**	1/21	100	3	–	95		–		39
**0.2 % RuO_2/Al_2O_3-ALD**	1/21	2000	0.03	10	110		–	0.01	40
**2 % Pt/ZIF-8 (encapsulation)**	1/5	100	2	3.3	200		–		41
**RuO_2**	1/5	100	3	6	90		–		42
**meso-RuO_2-O_2**	1/2.8	60	5.2	6.2	29				43
**3 % Ru/CeO_2**	1/5	100	3	nanochain	140	127	–		44
**5 % RuO_2/SnO_2-11**	1/1	25	7	–	150	125	–		45
**$Ru_{0.5}Cu_{0.5}/\gamma$-Al_2O_3**	1/1	150	2	9.2	–	122	–		46
**fcc-1% Ru/γ-Al_2O_3**	1/1	150	2	5.9	–	141	–		47
**2.92 Ru/SiO_2**	2/1	15	Plug-flow condition	1.8	150 (CO conversion 90%)	–	–	0.0132 (CO_2 formation rate)	48

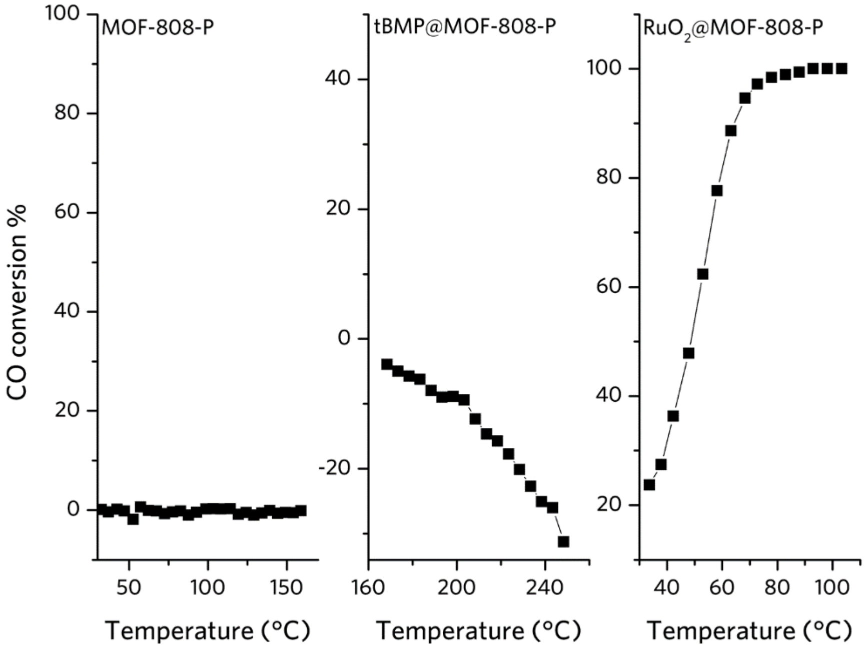

Supplementary Figure 19. CO oxidation tests for MOF-808-P, tBMP@MOF-808-P and RuO_2@MOF-808-P. Both MOF-808-P and tBMP@MOF-808-P are inactive for CO oxidation. The negative conversion observed for tBMP@MOF-808-P is likely due to tBMP desorption from the MOF. The catalysts were activated by Ar gas at 120 ^oC for 1h; catalysts mass:25.8 mg, WHSV= 120 $L{\cdot}g_{Ru}^{-1}{\cdot}h^{-1}$. Source data are provided as a Source Data file.

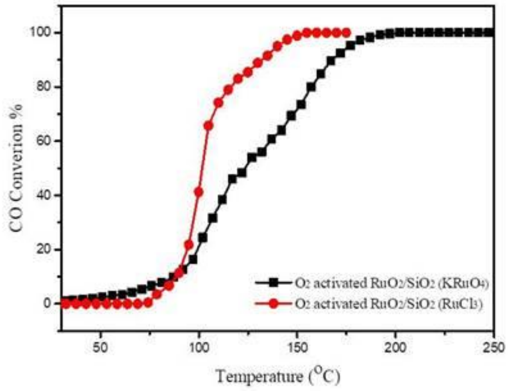

Supplementary Figure 20. CO oxidation tests for O_2 activated RuO_2/SiO_2 ($RuCl_3$) and RuO_2/SiO_2 ($KRuO_4$). The catalysts were activated by O_2 gas at 250 ^oC for 1h. The weight hourly space velocity (WHSV) is 2000 $L{\cdot}g_{Ru}^{-1}{\cdot}h^{-1}$. The catalytic results indicated that, for impregnation method, the CO oxidation performance for RuO_2/SiO_2 with $RuCl_3$ is better than that for RuO_2/SiO_2 with $KRuO_4$. Therefore, this excludes the precursor contribution (i.e. $KRuO_4$) to the superior performance of RuO_2@MOF-808-P. Source data are provided as a Source Data file.

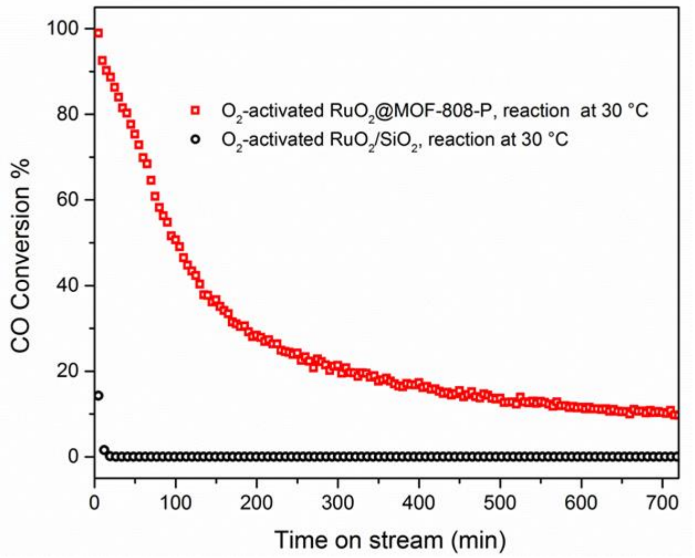

Supplementary Figure 21. Stability test using O_2-activated RuO_2/SiO_2 and RuO_2@MOF-808-P (tests condition: 400 $L \cdot g_{Ru}^{-1} \cdot h^{-1}$, 30 mg catalysts) at 30 °C. The results are consistent with those at 100 °C in Fig. 4d. The gradual deactivation of RuO_2@MOF-808-P at 30 °C is likely to be caused by formation of surface carbonates which can be seen from DRIFTS results below.[49] Source data are provided as a Source Data file.

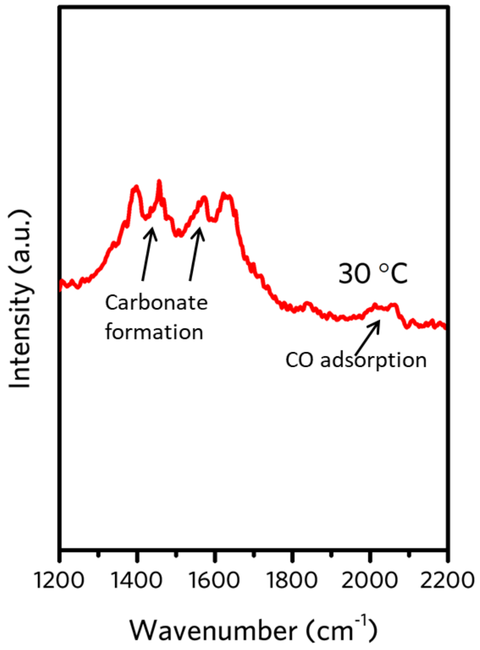

Supplementary Figure 22. Extended DRIFTS results of Fig. 3d. At 30 °C, the peak features indicating the formation of carbonates can be noticed.[43] Source data are provided as a Source Data file.

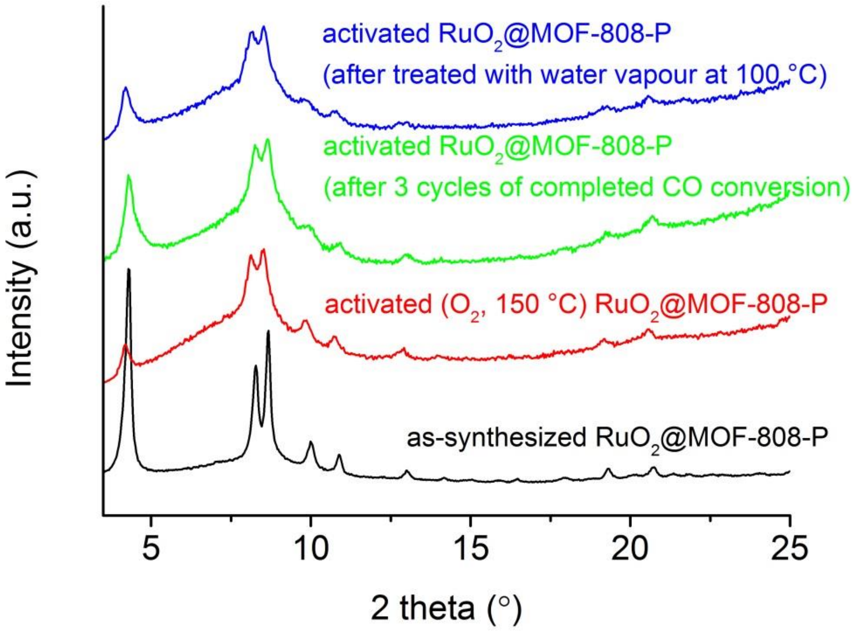

Supplementary Figure 23. PXRD patterns for RuO_2@MOF-808-P after treatments/tests labeled. The structure is mostly preserved after these treatments/tests. The PXRD experimental setup is the same as those mentioned in SI section 2.6. Source data are provided as a Source Data file.

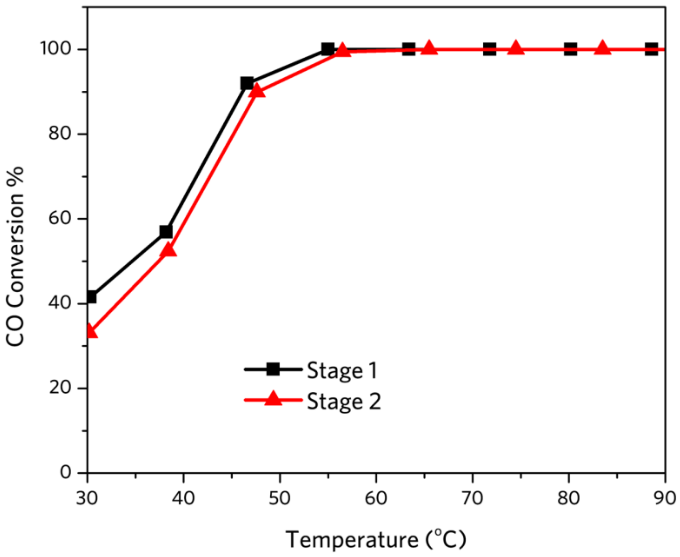

Supplementary Figure 24. CO oxidation tests for RuO_2@MOF-808-P which was tested after the standard O_2-activation mentioned in this work (stage 1) and tested again after being treated with 10 vol% water vapor at 100 °C for 60 minutes (stage 2). Catalysts mass: 30 mg, WHSV = 400 $L \cdot g_{Ru}^{-1} \cdot h^{-1}$. There is no decrease in the catalytic activity after the water treatment at high temperature. The results imply that the RuO_2@MOF-808-P catalysts have a high water tolerance. Source data are provided as a Source Data file.

5 Preliminary Results for Other Guest@Nanoporous-Host Systems Achieved Using Pourbaix Enabled Guest Synthesis (PEGS)

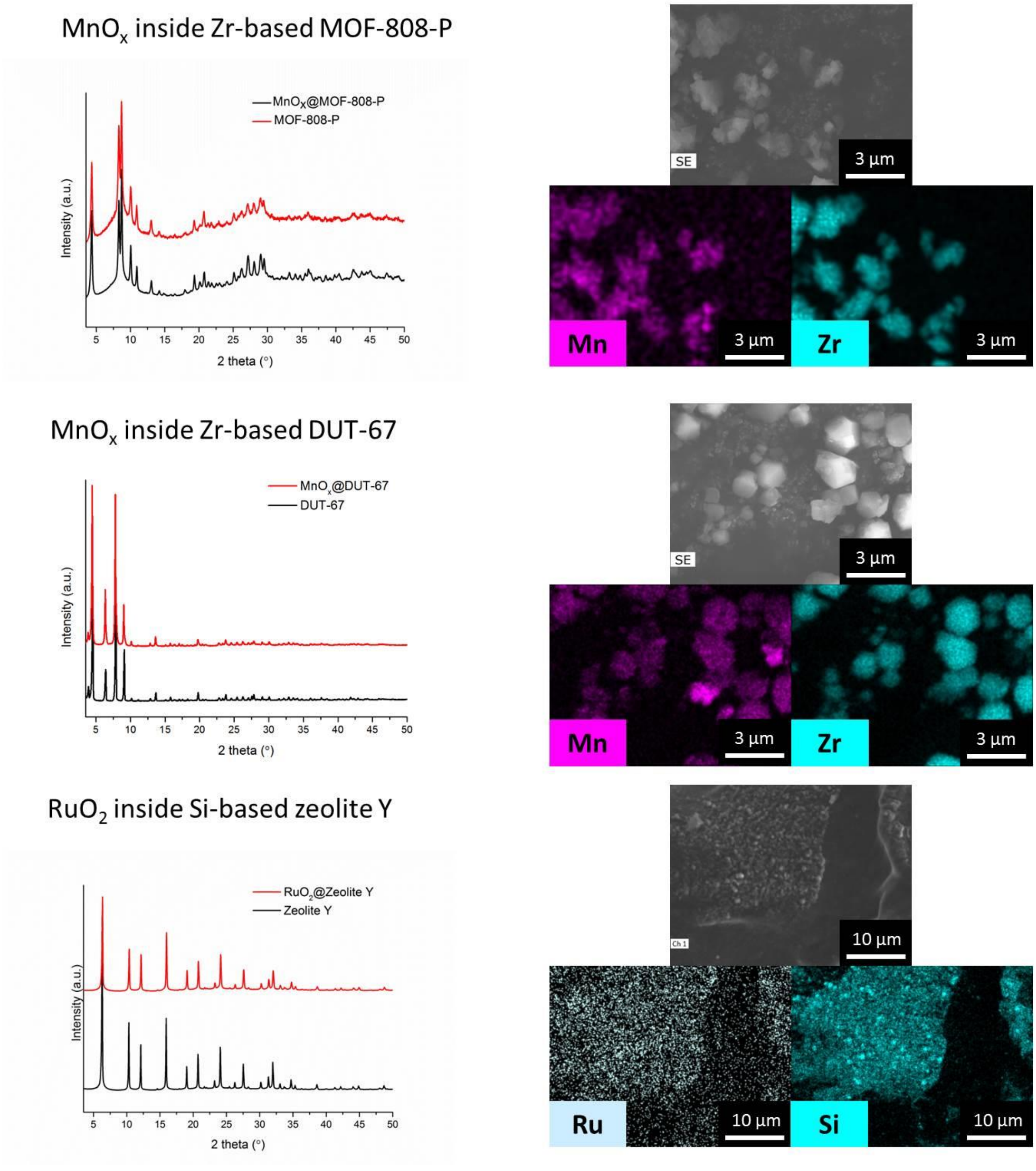

Supplementary Figure 25. PXRD and SEM-EDS results for manganese oxide (MnO_x) in MOF-808-P (top) and another Zr-based MOF (i.e. DUT-67[50]) (middle) and RuO_2 in a commercially available zeolite Y[51] from Alfa Aesar (bottom). Both of them are prepared using the methodology mentioned in this work (Supplementary Figure 1) to demonstrate the general applicability of the guest incorporation concept for a range of nanoporous materials. The precursor for MnO_x is 20 mM $KMnO_4$ (aq); tBMP is used as the reducing agent for all the samples. The relevant characterization methods can be referred back to the Supplementary Section 2.6. Source data and raw images are provided as a Source Data file.

As a further demonstration, we use the Pourbaix diagrams constructed with using Materials Project[52–54] to predict the potential PEGS conditions for Pt and Pd inside a MOF (Supplementary Figure 26). Briefly, since no stable Pt^{2+} is seen on the Pourbaix diagram for Pt (Supplementary Figure 26, left), it would be very difficult to use the Pt^{2+} for host (e.g. MOF) impregnation. Extra stabilization with ligands would be required in the Pt precursor. This explains why Pt precursors such as $[Pt(NH_3)_4]Cl_2$ rather than $PtCl_2$ is used for preparing Pt@MOF via solution-based synthesis, where extra NH_3 is involved to stabilize the Pt(II) salt.[2]

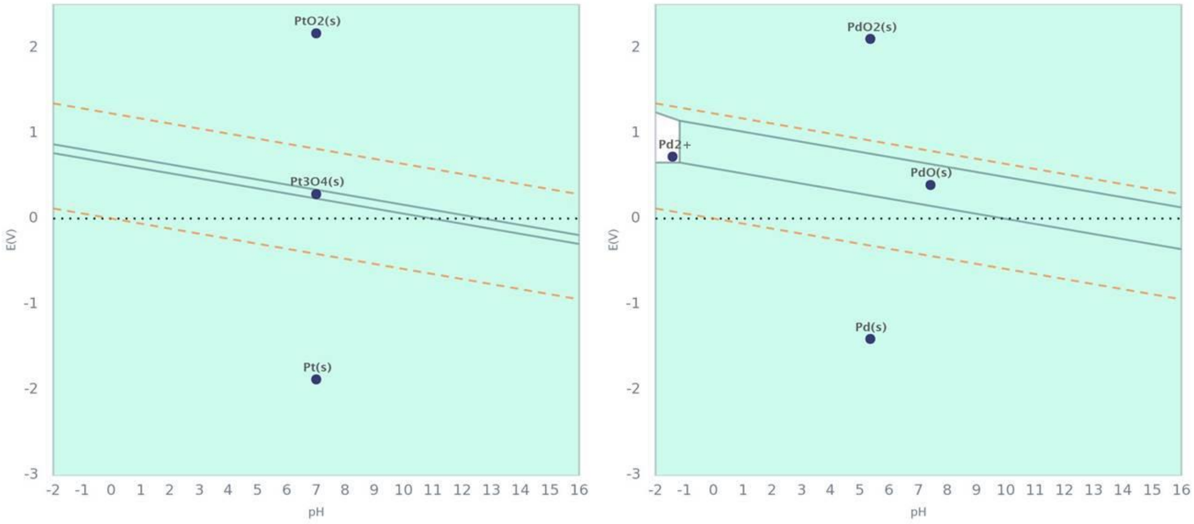

Supplementary Figure 26. Pourbaix diagrams for Pt [left, [Pt] = 10^{-2} mol·kg^{-1} (total aqueous mixture)] and Pd [right, [Pd] = 10^{-2} mol·kg^{-1} (total aqueous mixture)] are constructed using Materials Project.[52–54]

In contrast, according to the Pourbaix diagram for Pd (Supplementary Figure 26, right) Pd^{2+} is more ready to be used as the mobile precursors to impregnate MOF under low pH (stable Pd^{2+} phase shown at low pH even at fairly high Pd^{2+} concentration, 10^{-2} mol·kg^{-1} ≈ 10 mM). This Pourbaix diagram further rationalize the preparation of some published examples of Pd@MOF[1,2,55] with Pd(II) salts, such as $Pd(NO_3)_2$ and Pd(II) acetylacetonate. As an experimental verification, we first stabilized 0.106 g $Pd(NO_3)_2·H_2O$ in 20 ml HNO_3 (aq, 0.1 M). Although Pd^{2+} can be easily reduced to Pd^0 if the pH were unaltered, the $\Delta E_{reduction}$ can be more than 0.5 V if the pH becomes significantly higher. Since the pH after the reaction is very likely to be higher than the acidic Pd^{2+} solution (i.e. precursor solution), we used $NaBH_4$ as reducing agent (with standard reduction potential of -1.24 V versus SHE) which can sufficiently reduce the Pd^{2+} to Pd^0 regardless the pH change. To prepared the $NaBH_4$ (aq) solution, 0.15 g $NaBH_4$ (excess amount) were dissolved by 280 ml Milli-Q water which has the pH value of ca. 8. Note that unlike tBMP for RuO_2, $NaBH_4$ has no capability (*e.g.* hydrophobic-hydrophilic interaction and temperature-controlled elective desorption) to control the Pd loading position. Since the redox reaction would take place in aqueous condition with pH value slightly less than 8 (due to excess $NaBH_4$), we chose the MOF-808-P as the host.

To load Pd in MOF-808-P (i.e. to form Pd@MOF-808-P), we first impregnated $Pd(NO_3)_2$ solution in the dried MOF-808-P. The $Pd(NO_3)_2$(aq)@MOF-808-P was then reacted with the prepared $NaBH_4$ solution at room temperature for 10 min. Black suspension was observed upon the reaction indicating the formation of metallic Pd^0. The product was collected by centrifugation and washed with water and ethanol. It was then dried in vacuum oven at room temperature for 24 h.

Since there is no control about the Pd loading position for the as-prepared Pd@MOF-808-P, metallic Pd^0 forms both inside the MOF and on its outer surface as revealed in Supplementary Figure 27. Some Pd particles can agglomerate on the outer surface of the MOF (without MOF pore confinement). The presence large Pd particles are also confirmed by a peak at ca. 40° (for Pd^0) in PXRD pattern in Supplementary Figure 28 for Pd@MOF-808-P. Meanwhile, the PXRD patterns also verify the preserve of the MOF's structure throughout the synthesis. Hence, our PEGS strategy works for preparing Pd@MOF-808-P.

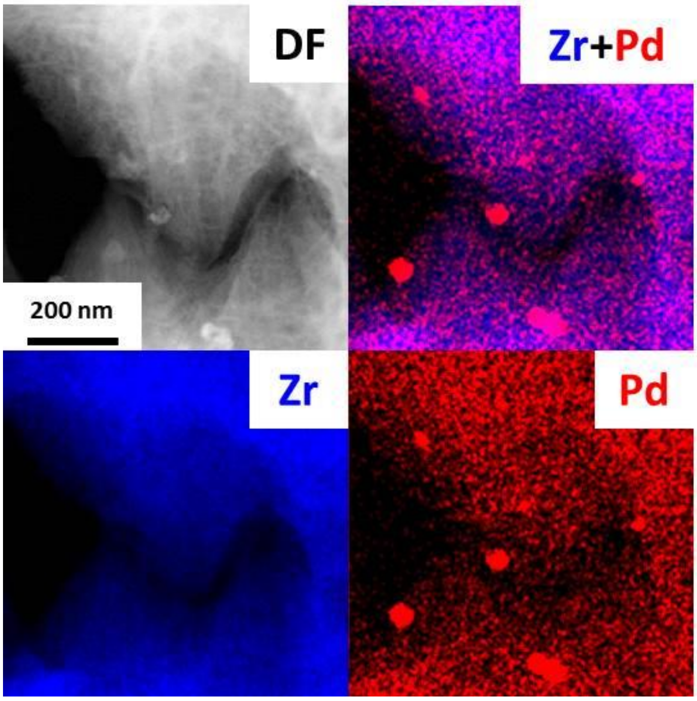

Supplementary Figure 27. A DF-STEM image for Pd@MOF-808-P and its corresponding STEM-EDS mappings for Zr and Pd. Raw images are provided as a Source Data file.

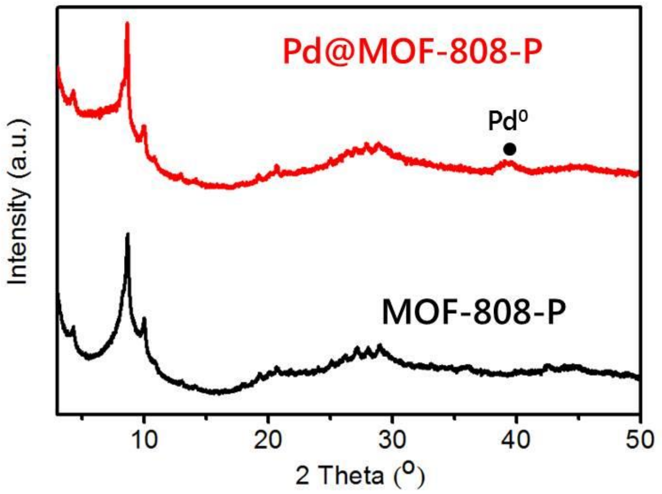

Supplementary Figure 28. PXRD patterns for MOF-808-P and Pd loaded MOF-808-P (i.e. Pd@MOF-808-P). The PXRD results were collected on a Rigaku D/Max 2500 diffractometer using a Cu Kα (λ = 1.5406 Å) radiation source and scanning rate of $1^{o}\cdot min^{-1}$. Source data are provided as a Source Data file.

References

1. Meilikhov, M. et al. Metals@MOFs - Loading MOFs with metal nanoparticles for hybrid functions. *Eur. J. Inorg. Chem.* **2010**, 3701–3714 (2010).

2. Juan-Alcañiz, J., Gascon, J. & Kapteijn, F. Metal–organic frameworks as scaffolds for the encapsulation of active species: State of the art and future perspectives. *J. Mater. Chem.* **22**, 10102-10118 (2012).

3. Dhakshinamoorthy, A. & Garcia, H. Catalysis by metal nanoparticles embedded on metal–organic frameworks. *Chem. Soc. Rev.* **41**, 5262–5284 (2012).

4. Gascon, J., Corma, A., Kapteijn, F. & Llabrés i Xamena, F. X. Metal organic framework catalysis: Quo vadis ? *ACS Catal.* **4**, 361–378 (2014).

5. Allendorf, M. D. et al. Guest-induced emergent properties in metal–organic frameworks. *J. Phys. Chem. Lett.* **6**, 1182–1195 (2015).

6. Chen, L., Luque, R. & Li, Y. Controllable design of tunable nanostructures inside metal–organic frameworks. *Chem. Soc. Rev.* **46**, 4614–4630 (2017).

7. Huang, Y.-B., Liang, J., Wang, X.-S. & Cao, R. Multifunctional metal–organic framework catalysts: synergistic catalysis and tandem reactions. *Chem. Soc. Rev.* **46**, 126–157 (2017).

8. Landmesser, H. & Miessner, H. Interaction of Co with ruthenium supported on dealuminated Y-zeolite - Evidence for the formation of a ruthenium tricarbonyl. *J. Phys. Chem.* **95**, 10544–10546 (1991).

9. Li, J. et al. Sub-nm ruthenium cluster as an efficient and robust catalyst for decomposition and synthesis of ammonia: Break the “size shackles”. *Nano Res.* **11**, 4774-4785 (2018).

10. Bleloch, A. et al. Modified mesoporous silicate MCM-41 materials: immobilised perruthenate—a new highly active heterogeneous oxidation catalyst for clean organic synthesis using molecular oxygen. *Chem. Commun.* **18**, 1907–1908 (1999).

11. Zhan, B.-Z. et al. Zeolite-confined nano-RuO_2 : A green, selective, and efficient catalyst for aerobic alcohol oxidation. *J. Am. Chem. Soc.* **125**, 2195–2199 (2003).

12. Schröder, F. et al. Ruthenium nanoparticles inside porous [$Zn_4O(bdc)_3$] by hydrogenolysis of adsorbed [Ru(cod)(cot)] : A solid-state reference system for surfactant-stabilized ruthenium colloids. *J. Am. Chem. Soc.* **130**, 6119–6130 (2008).

13. Maza, W. A., Padilla, R. & Morris, A. J. Concentration dependent dimensionality of resonance energy transfer in a postsynthetically doped morphologically homologous analogue of UiO-67 MOF with a ruthenium(II) polypyridyl complex. *J. Am. Chem. Soc.* **137**, 8161–8168 (2015).

14. Maza, W. A. et al. Ruthenium(II)-polypyridyl zirconium(IV) metal–organic frameworks as a new class of sensitized solar cells. *Chem. Sci.* **7**, 719–727 (2016).

15. Jiang, H. et al. Au@ZIF-8 : CO oxidation over gold nanoparticles deposited to metal-organic framework. *J. Am. Chem. Soc.* **2**, 11302–11303 (2009).

16. Zhuang, G. et al. The effect of N-containing supports on catalytic CO oxidation activity over highly dispersed Pt/UiO-67. *Eur. J. Inorg. Chem.* **2017**, 172–178 (2017).

17. Wang, W. et al. Metal-organic framework as a host for synthesis of nanoscale Co_3O_4 as an active catalyst for CO oxidation. *Catal. Commun.* **12**, 875–879 (2011).

18. Lin, A., Ibrahim, A. A., Arab, P., El-Kaderi, H. M. & El-Shall, M. S. Palladium nanoparticles supported on Ce-metal-organic framework for efficient CO oxidation and low-temperature CO_2 capture. *ACS Appl. Mater. Interfaces* **9**, 17961–17968 (2017).

19. Pourbaix, M. *Atlas of Electrochemical Equilibria in Aqueous Solutions* (Pergamon Press, New York, 1966).

20. Campbell, J. A. & Whiteker, R. A. A periodic table based on potential-pH diagrams. *J. Chem. Educ.* **46**, 90-92 (1969).

21. Povar, I. & Spinu, O. Ruthenium redox equilibria: 3. Pourbaix diagrams for the systems Ru-H_2O and Ru-Cl^--H_2O. *J. Electrochem. Sci. Eng.* **6**, 145-153 (2016).

22. Povar, I. & Spinu, O. Ruthenium redox equilibria: 1. Thermodynamic stability of Ru(III) and Ru(IV) hydroxides. *J. Electrochem. Sci. Eng.* **6**, 123-133 (2016).

23. Povar, I. & Spinu, O. Ruthenium redox equilibria: 2. Thermodynamic analysis of disproportionation and comproportionation conditions. *J. Electrochem. Sci. Eng.* **6**, 135-143 (2016).

24. Revie, R. W. & Uhlig, H. H. *Corrosion and Corrosion Control: An Introduction to Corrosion Science and Engineering.* (John Wiley & Sons, Inc., New Jersey, 2008).

25. Jiang, J. et al. Superacidity in sulfated metal–organic framework-808. *J. Am. Chem. Soc.* **136**, 12844–12847 (2014).

26. Yohe, G. R. et al. The oxidation of 2,6-di-tert-butyl-4-methylphenol. *J. Org. Chem.* **21**, 1289–1292 (1956).

27. Richards, J. A. & Evans, D. H. Electrochemical oxidation of 2,6-di-tert-butyl-4-isopropylphenol. *J. Electroanal. Chem. Interfacial Electrochem.* **81**, 171–187 (1977).

28. Wang, T. et al. Functional conductive nanomaterials via polymerisation in nano-channels: PEDOT in a MOF. *Mater. Horiz.* **4**, 64–71 (2017).

29. Le Ouay, B. et al. Nanostructuration of PEDOT in porous coordination polymers for tunable porosity and conductivity. *J. Am. Chem. Soc.* **138**, 10088–10091 (2016).

30. Furukawa, H. et al. Water adsorption in porous metal–organic frameworks and related materials. *J. Am. Chem. Soc.* **136**, 4369–4381 (2014).

31. Keattch, C. J. & Redfern, J. P. The preparation and properties of a hydrous ruthenium oxide. *J. Less Common Met.* **4**, 460–465 (1962).

32. Velázquez-Palenzuela, A. et al. Structural properties of unsupported Pt−Ru nanoparticles as anodic catalyst for proton exchange membrane fuel cells. *J. Phys. Chem. C* **114**, 4399–4407 (2010).

33. Zhang, X. & Chan, K. Y. Water-in-oil microemulsion synthesis of platinum-ruthenium nanoparticles, their characterization and electrocatalytic properties. *Chem. Mater.* **15**, 451–459 (2003).

34. Rolison, D. R., Hagans, P. L., Swider, K. E. & Long, J. W. Role of hydrous ruthenium oxide in Pt−Ru direct methanol fuel cell anode electrocatalysts: The importance of mixed electron/proton conductivity. *Langmuir* **15**, 774–779 (1999).

35. Wang, X. et al. Uncoordinated amine groups of metal–organic frameworks to anchor single Ru sites as chemoselective catalysts toward the hydrogenation of quinoline. *J. Am. Chem. Soc.* **139**, 9419–9422 (2017).

36. Wang, T. et al. Bottom-up formation of carbon-based structures with multilevel hierarchy from MOF–guest polyhedra. *J. Am. Chem. Soc.* **140**, 6130–6136 (2018).

37. Anovitz, L. M. & Cole, D. R. Characterization and analysis of porosity and pore structures. *Rev. Mineral. Geochem.* **80**, 61–164 (2015).

38. Joo, S. H. et al. Size Effect of ruthenium nanoparticles in catalytic carbon monoxide oxidation. *Nano Lett.* **10**, 2709–2713 (2010).

39. Cui, X., Wang, Y., Chen, L. & Shi, J. Synergetic catalytic effects in tri-component mesostructured Ru-Cu-Ce oxide nanocomposite in CO oxidation. *ChemCatChem* **6**, 2860–2871 (2014).

40. Kim, I. H. et al. CO oxidation catalyzed by RuO2nanoparticles supported on mesoporous Al_2O_3prepared via atomic layer deposition. *Curr. Appl. Phys.* **16**, 1407–1412 (2016).

41. Lu, G. et al. Imparting functionality to a metal-organic framework material by controlled nanoparticle encapsulation. *Nat. Chem.* **4**, 310–316 (2012).

42. Shen, W. et al. Synthesis and CO oxidation catalytic character of high surface area ruthenium dioxide replicated by cubic mesoporous silica. *Chem. Lett.* **34**, 390–391 (2005).

43. Park, J.-N. et al. Room-temperature CO oxidation over a highly ordered mesoporous RuO2 catalyst. *React. Kinet. Mech. Catal.* **103**, 87–99 (2011).

44. Sreedhala, S. & Vinod, C. P. Surfactant assisted formation of ruthenium nanochains under mild conditions and their catalytic CO oxidation activity. *Chem. Commun.* **51**, 10178–10181 (2015).

45. Yu, J., Zhao, D., Xu, X., Wang, X. & Zhang, N. Study on RuO_2/SnO_2 : Novel and active catalysts for CO and CH_4 oxidation. *ChemCatChem* **4**, 1122–1132 (2012)

46. Huang, B. et al. Solid-solution alloying of immiscible Ru and Cu with enhanced CO oxidation activity. *J. Am. Chem. Soc.* **139**, 4643–4646 (2017).

47. Kusada, K. et al. Discovery of face-centered-cubic ruthenium nanoparticles: Facile size-controlled synthesis using the chemical reduction method. *J. Am. Chem. Soc.* **135**, 5493–5496 (2013).

48. Liang, C., Narkhede, V., Aßmann, J. & Muhler, M. Controlled synthesis of supported ruthenium catalysts for CO oxidation by organometallic chemical vapor deposition. *Stud. Surf. Sci. Catal.* **162**, 473–480 (2006).

49. Over, H. Surface chemistry of ruthenium dioxide in heterogeneous catalysis and electrocatalysis: From fundamental to applied research. *Chem. Rev.* **112**, 3356–3426 (2012).

50. Bon, V., Senkovska, I., Baburin, I. a & Kaskel, S. Zr- and Hf-based metal–organic frameworks: Tracking down the polymorphism. *Cryst. Growth Des.* **13**, 1231–1237 (2013).

51. Herron, N. A cobalt oxygen carrier in zeolite Y. A molecular “ship in a bottle”. *Inorg. Chem.* **25**, 4714–4717 (1986).

52. Singh, A. K. et al. Electrochemical stability of metastable materials. *Chem. Mater.* **29**, 10159–10167 (2017).

53. Jain, A. et al. Commentary: The materials project: A materials genome approach to accelerating materials innovation. *APL Mater.* **1**, 011002 (2013).

54. Persson, K. A., Waldwick, B., Lazic, P. & Ceder, G. Prediction of solid-aqueous equilibria: Scheme to combine first-principles calculations of solids with experimental aqueous states. *Phys. Rev. B* **85**, 235438 (2012).

55. Pascanu, V. et al. Sustainable catalysis: Rational Pd loading on MIL-101Cr-NH_2 for more efficient and recyclable Suzuki-Miyaura reactions. *Chem. Eur. J.* **19**, 17483–17493 (2013).

56. Chakrapani, K. & Sampath, S. The dual role of borohydride depending on reaction temperature: synthesis of iridium and iridium oxide. *Chem. Commun.* **51,** 9690–9693 (2015).
