# Supplementary material for: Rational approach to guest confinement inside MOF cavities for low-temperature catalysis
Source: Nat Commun. 2019 Mar 22;10:1340. doi: 10.1038/s41467-019-08972-x (PMC6430784; doi:10.1038/s41467-019-08972-x)
Supplement: Supplementary file 3 — Description of Additional Supplementary Files [file 41467_2019_8972_MOESM3_ESM.docx]

**Description of Additional Supplementary Files**

File Name: Supplementary Data 1

Description: Source dataset for results in the main manuscript.

File Name: Supplementary Data 2

Description: Source dataset for results in Supplementary Information.
