# Supplementary material for: Rational approach to guest confinement inside MOF cavities for low-temperature catalysis
Source: Nat Commun. 2019 Mar 22;10:1340. doi: 10.1038/s41467-019-08972-x (PMC6430784; doi:10.1038/s41467-019-08972-x)
Supplement: Supplementary file 2 — Peer Review File [file 41467_2019_8972_MOESM2_ESM.pdf]

Reviewers' comments:

Reviewer #1 (Remarks to the Author):

Excellent work, worth publication almost as such in Nature Communications. The work has been carefully developed and provides an innovative and rational approach to guest formation inside MOF cavities. Certain references missing from the introduction should be cited (e.g. Science 2018, 359, 206; Dalton Trans. 2018, 4, 3663; Chem. Soc. Rev. 2017, 46, 4614) to provide a better overview on the stabilisation of specis within MOF structures. The manuscript may need some revisions to avoid typos and mistakes (see references) but aside of this, the manuscript will make an excellent contribution for the journal, being strongly recommended for publication in Nature Communications.

Reviewer #2 (Remarks to the Author):

Smoukov, Fu and colleagues report a very impressive work on the encapsulation of metal oxides in the cavities of porous hosts. By making use of Pourbaix diagrams, the authors are able to identify the most adequate reducing agent, place it in the pores of the host prior to impregnation of the metal precursor and selectively form metal nanoparticles inside of the porosity while avoiding the deposition of nanoparticles in the outer surface.

The article is well written, easy to follow and catalytic tests and characterisation are rigorous and sound. I strongly support publication of this nice work and I am looking forward to follow up works to expand on the potential of this smart way of looking at nanoparticle encapsulation.

Having said this, I find the authors could further improve the work by answering the comments below:

1) The reduction in adsorption capacity in figure 2 b is much larger than the amount of RuO_2 loaded in the MOF. Considering that this is a 3D MOF structure that should not be prone to cavity blocking this referee wonders what is the reason for this reduction in pore volume.

2) From IR spectroscopy it seems quite clear that the strength of interaction between CO and RuO_2 is weakened when the latter is confined into the MOF cavities. However, this seems to be in contradiction with the higher reducibility of the confined clusters. I suggest the authors should elaborate further on this.

Reviewer #3 (Remarks to the Author):

This work reports the synthesis of nano-sized RuO_2 particles inside the pores of MOF-808. The particles were synthesized through tBMP reduction of RuO_4^- under basic conditions. The catalytic performance of the RuO_2@MOF-808 for CO oxidation is much higher than RuO_2-SiO_2. The high catalytic CO oxidation of RuO_2@MOF-808 at low temperature was proposed to be a result of weak coordination of CO on the surface of RuO_2. Encapsulation of NPs in MOF channels has been well established in the past decade and catalytic CO oxidation is not of practical significance. As such, this manuscript falls short of the high standard of Nat. Commun. and should be submitted to a more specialized journal.

1. RuO_2/SiO_2 was used as a control to highlight the superior performance of RuO_2@MOF-808. But the RuO_2/SiO_2 catalysis was prepared through a very different method, impregnation of Aerosil with $RuCl_3 \cdot 1.5H_2O$ under neutral conditions (Ref 46, Catalysis Communications 4 (2003) 91–96); The RuO_2/SiO_2 is not even porous, thus any comparison over catalytic activity is not fair. A more relevant

control should be using mesoporous silica and prepare RuO_2 using the same RuO_4^- reduction method under basic condition. Because synthetic procedure to access RuO_2 nanomaterial could lead to dramatically different surface functionalities.

2. In Figure 3c-d, the authors compared the IR signature of CO absorption on RuO_2-SiO_2 and RuO_2-MOF-808 and concluded “Ru-CO interactions are also weaker on the MOF-confined RuO_2 surface”. However, DRIFTS IR signal generally arises from the reflection on material surface. In the case of nonporous RuO_2-SiO_2, the Ru active sites are located on the material surface, thus the IR signals are stronger. But in the case of RuO_2@MOF-808, the RuO_2 particles are wrapped inside the MOF channel. And this could be the real reason for the weaker DRIFTS IR signal. Did the authors do any transmissive IR? C-O stretching frequency shifts might be more indicative of such interaction strength but the authors did not observe any shift.

3. The authors determined the Ru content in RuO_2@MOF-808 by ICP-OES. Did they determine the amount of K? Because the authors used $KRuO_4$ as synthetic precursor. The amount of K can indicate the amount of residue $KRuO_4$ trapped in the channel through ionic interactions, and the surface charge properties of the RuO_2 particle. For example, high density of (RuO_4^-)(K^+) functionalities that might give rise to the improved catalytic performance.

4. How do the authors confirm the identity of RuO_2 particle inside the MOF channel? Because TEM images are not of high enough quality for determining accurate structure; did the authors take PXRD of the trapped RuO_2 particle?

5. Although the authors successfully incorporated the RuO_2 NPs into MOF-808-p, however, more examples are needed to show the generality of the synthetic approach. For example, can other NPs (such as Pt, Pd) be confined into the MOFs by such an approach?

6. To prove the generality of this synthetic method, the authors also need to try more MOFs as hosts. Why did the authors choose MOF-808-p but not MOF-808 as the host? Will other MOFs (such as MOF-5 and HKUST-1) also work as hosts?

Reviewers' comments:

Reviewer #1 (Remarks to the Author):

Excellent work, worth publication almost as such in Nature Communications. The work has been carefully developed and provides an innovative and rational approach to guest formation inside MOF cavities. Certain references missing from the introduction should be cited (e.g. Science 2018, 359, 206; Dalton Trans. 2018, 4, 3663; Chem. Soc. Rev. 2017, 46, 4614) to provide a better overview on the stabilisation of species within MOF structures.

Response: We thank the reviewer for the positive feedback. We have improved our work based on the reviewer's advice. Based on the reviewer's suggestion, we have cited the suggested papers (references 4, 19 and 26). We have also enhanced the overview on the stabilization of species within MOF on page 4 in the manuscript by adding: "*By immobilizing the guests inside MOFs, guest aggregation/fusion can be effectively prevented.*[4,19]"

The manuscript may need some revisions to avoid typos and mistakes (see references) but aside of this, the manuscript will make an excellent contribution for the journal, being strongly recommended for publication in Nature Communications.

Response: We thank the reviewer again for recognizing the value of the work. We have double-checked the misspellings particularly for the references.

Reviewer #2 (Remarks to the Author):

Smoukov, Fu and colleagues report a very impressive work on the encapsulation of metal oxides in the cavities of porous hosts. By making use of Pourbaix diagrams, the authors are able to identify the most adequate reducing agent, place it in the pores of the host prior to impregnation of the metal precursor and selectively form metal nanoparticles inside of the porosity while avoiding the deposition of nanoparticles in the outer surface.

The article is well written, easy to follow and catalytic tests and characterisation are rigorous and sound. I strongly support publication of this nice work and I am looking forward to follow up works to expand on the potential of this smart way of looking at nanoparticle encapsulation.

Response: We are very appreciative of the reviewer's recognition and suggestions. We are also very delighted to see that our "ship-in-a-bottle" of interest to other specialists in the field and that it can be useful for their research.

Having said this, I find the authors could further improve the work by answering the comments below:

1) The reduction in adsorption capacity in figure 2 b is much larger than the amount of RuO_2 loaded in the MOF. Considering that this is a 3D MOF structure that should not be prone to cavity blocking this referee wonders what is the reason for this reduction in pore volume.

Response: We would like to thank the reviewer for raising the query. The reduction in the adsorbed volume of N_2 can be due to two factors: (i) the formation of within-the-pore RuO_2 and (ii) partial pore collapse and amorphization during the post-synthetic modifications.[1–3] This is supported by the PXRD results comparing the dried MOF with the as-prepared RuO_2@MOF as shown below (also as Figure S13 added to the SI).

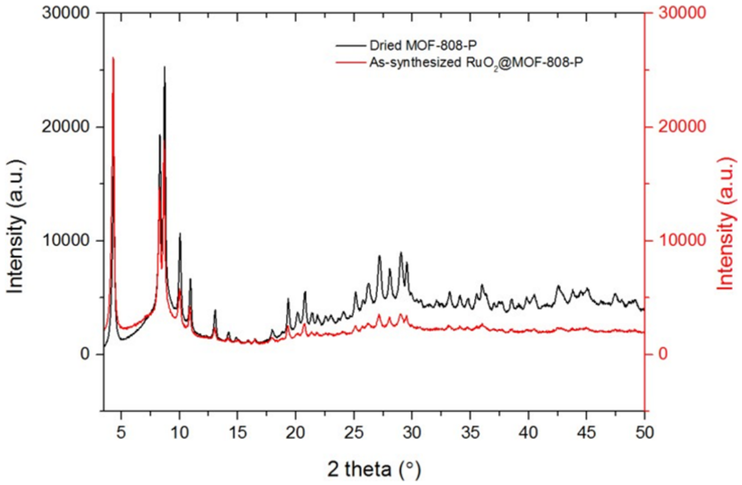

"Figure S13. More detailed PXRD patterns for dried MOF-808-P and as-synthesized RuO_2@MOF-808-P. *The MOF structure is more defective after RuO_2 inclusion (i.e. post-synthetic modification), as PXRD peaks (long-range ordering features) can be hardly observed above 40° (2 theta) for as-prepared RuO_2@MOF-808-P*

(in red) as compared to dried MOF (in black). Note that the variation of intensity in peak below 5° (2 theta) can be largely influenced by the presence of guest compounds.[36]"

In fact, similar reduction in pore volume has been found in other MOF-guest systems formed by post-synthetic modification,[4] where the MOF has three dimensional pore structure.

We improved our manuscript accordingly by adding (on page 9 in the manuscript) "*Note that further reduction in adsorbed volume of N_2 can be explained by partial pore collapse and/or amorphization.[24,48,49] This is supported by the disappearance of PXRD peaks (i.e. less ordered) above 40° for as-prepared RuO2@MOF-808-P as compared with dried MOF-808-P (Figure S13).*"

2) From IR spectroscopy it seems quite clear that the strength of interaction between CO and RuO_2 is weakened when the latter is confined into the MOF cavities. However, this seems to be in contradiction with the higher reducibility of the confined clusters. I suggest the authors should elaborate further on this.

Response: We thank the reviewer for the suggestion. The conclusions for "*interaction between CO and RuO_2 is weakened*" and "*higher reducibility of the confined clusters*" are drawn from different experiments to characterize two different interactions, *i.e.* interaction of CO with RuO_2 surface and Ru-O interactions within the RuO_2.

"*interaction between CO and RuO_2 is weakened*" is revealed by temperature-dependent desorption results (Figure 3b), which is obtained by pre-adsorbing CO at room temperature and increasing the temperature in Ar to desorb CO from the RuO_2 surface at the low temperatures (30 - 150 °C). The temperature-dependent desorption focuses on the interaction adsorbed CO with RuO_2 surface.

"*higher reducibility of the confined clusters*" is concluded from CO-TPR results (Figure 3a) at higher temperatures (160 – 300 °C), which probes the RuO_2 reduction by CO (in CO/He gas) not only at the surface, but when CO diffuses into the RuO_2 matrix. CO-TPR is used for determining how easy the O in RuO_2 can be taken by CO, *i.e.* demonstrating the relative Ru-O bond strength within the RuO_2 catalyst. This is likely the result of electronic confinement effects of RuO_2 in cavities,[5] which cause distortion of its bonding orbitals.

In MOF-confined RuO_2, both interaction of CO with RuO_2 surface and Ru-O interactions within the RuO_2 are weakened. Therefore, it is possible to have high reducibility (due to weaker Ru-O interactions within the RuO_2) when RuO_2-CO surface interaction becomes weaker.

To address the concern and to avoid any confusion, we have modified the paragraph starting from "*Ru-O interactions within the RuO_2 were tested …*" on page 10 in the manuscript and the paragraph starting from "*Weaker interaction of CO with the MOF-confined RuO_2 surface …*" on page 11 in the manuscript.

Reviewer #3 (Remarks to the Author):

This work reports the synthesis of nano-sized RuO_2 particles inside the pores of MOF-808. The particles were synthesized through tBMP reduction of RuO_4^- under basic conditions. The catalytic performance of the RuO_2@MOF-808 for CO oxidation is much higher than RuO_2-SiO_2. The high catalytic CO oxidation of RuO_2@MOF-808 at low temperature was proposed to be a result of weak coordination of CO on the surface of RuO_2. Encapsulation of NPs in MOF channels has been well established in the past decade and catalytic CO oxidation is not of practical significance. As such, this manuscript falls short of the high standard of Nat. Commun. and should be submitted to a more specialized journal.

Response: We thank the reviewer for reviewing our manuscript. We agree with the reviewer that many NPs (*i.e.* guest moieties) have been encapsulated in MOF cavities/channels since the 2000s. The syntheses for guest@MOF are very different, however, from those used for making bulk materials or free nanoparticles as they must be compatible with both the stability and small pore sizes of the MOFs. As a consequence, although there are a number of examples for metal NPs in MOF systems, there are no systematic guidelines for guest@MOF synthesis, especially when it comes to compounds such as oxides and sulphides. Here, we have shown a rational approach to guest@MOF systems, and to find reagents and conditions which would not destroy the MOF, which is the essence of our PEGS strategy (introduced in the manuscript). **More importantly, we use rational preparation of RuO_2@MOF-808-P as a tutorial to introduce the PEGS strategy, which allows the formation of guests in a more controlled (in terms of products), flexible (in terms of preparation conditions) and systematic (in terms of guests chemistries) way.** The strategy could be applied to a wider variety of guest@MOF systems with the approach based on the Pourbaix diagrams that are available from data base (*e.g.* Materials Project, https://materialsproject.org/). It could also be extended to other porous hosts such as zeolites with reasonable stability in aqueous solutions.[6] We have modified this significance in the manuscript on page 3 in the introduction starting from:" *Loading guests (e.g. molecules, clusters or particles) inside…*" as well as on page 16 in the conclusion starting from "*In summary, we use a preparation of…*"

The CO oxidation is used here as a probe reaction to investigate the nature of the catalysts and the catalytic performance. The prominent advantage of CO oxidation as a probe reaction is that it is a simple reaction with Langmuir-Hinshelwood mechanism which has been studied thoroughly from the last decades and is always an attractive reaction in the heterogeneous catalysis.[7,8] The other advantage is that the spectroscopic information about adsorption site and adsorption geometries of CO on many kinds of metal surface is well-documented, which is useful for further understanding the dynamic behavior during reaction process on the microscopic level.[9,10]

Furthermore, incomplete carbon combustion in both vehicles and industry produces CO that still threatens the environment and people's lives (*i.e.* CO poisoning), so CO oxidation is of undoubtable practical significance. It is a key reaction in respiratory protection and industrial air purification (particularly at low temperatures and in humid

air).[11–14] We have improved about this significance in the manuscript on page 10, which starts with the words: "*In heterogeneous catalysis …*"

1. RuO_2/SiO_2 was used as a control to highlight the superior performance of RuO_2@MOF-808. But the RuO_2/SiO_2 catalysis was prepared through a very different method, impregnation of Aerosil with $RuCl_3 \cdot 1.5H_2O$ under neutral conditions (Ref 46, Catalysis Communications 4 (2003) 91–96); The RuO_2/SiO_2 is not even porous, thus any comparison over catalytic activity is not fair. A more relevant control should be using mesoporous silica and prepare RuO_2 using the same RuO_4^- reduction method under basic condition. Because synthetic procedure to access RuO_2 nanomaterial could lead to dramatically different surface functionalities.

Response: We thank the reviewer for the comment and the opportunity to clarify a point that was vague. Though we cited the procedure from Ref. 46 (Ref. 58 in the revised version), we actually used mesoporous SiO_2 for this experiment. N_2 adsorption measurement display Type IV adsorption isotherm shape [15] showing the SiO_2 is mesoporous with the pore diameter ranging from 5 to 10 nm. We have added the N_2 physisorption isotherm to the SI as an additional Figure S16. We also corrected our schematics for RuO_2/SiO_2 shown in Figure 3 in the manuscript. We include both figures at the end of this response. We have added "*a commercially available mesoporous SiO_2*" on page 10. Note that our SiO_2-supported RuO_2 has CO oxidation performance comparable to SiO_2-supported RuO_2 prepared by Muhler *et al.*[16].

For reactivity, we show RuO_2@MOF-808-P is superior in CO oxidation performance to many other RuO_2-based catalysts (Table S1). But we agree with the reviewer that the method for reduction of Ru is not the perfect control – we only used it as it is the standard one for preparation of RuOx catalyst NPs. Therefore, we agree that it is not only the confinement but also the nature of the material that is likely to cause the difference in reactivity of the two samples. And we have modified the text in the manuscript on page 16 to reflect this by adding: "*These modulated interactions can be attributed to the confined microenvironment provided by MOF[50,51,59] and/or unique surface chemistry of RuO_2 introduced by the PEGS method.*" on page 16.

To address the concern of the reviewer about the effect of different Ru-based precursors we synthesized two parallel samples with $KRuO_4$ and $RuCl_3$ as precursors for RuO_2/SiO_2 respectively. Both RuO_2/SiO_2 samples were prepared under the same conditions. The CO conversion performance (Figure S20) for the RuO_2/SiO_2 made with $RuCl_3$ is slightly better than that for the RuO_2/SiO_2 made with $KRuO_4$. We include these results as Figure S20 and at the end of the response to this comment. We have also added "*We also exclude contribution of the precursor (i.e. $KRuO_4$) to this superior catalytic performance of RuO_2@MOF-808-P by showing that CO oxidation performance for RuO_2/SiO_2 with $RuCl_3$ is better than that for RuO_2/SiO_2 with $KRuO_4$ (Figure S20).*" on page 15 in the manuscript.

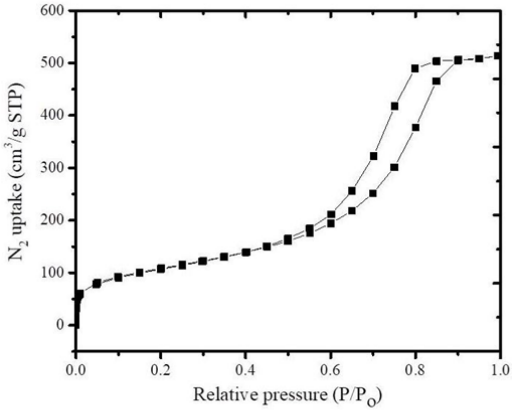

"Figure S16. Nitrogen physisorption isotherm of the SiO_2 support, *which has typical Type IV isotherm shape*[37] *showing the mesoporous SiO_2 with 356.1 m^2/g surface area. Further analysis shows the pore diameter is 5-10 nm."*

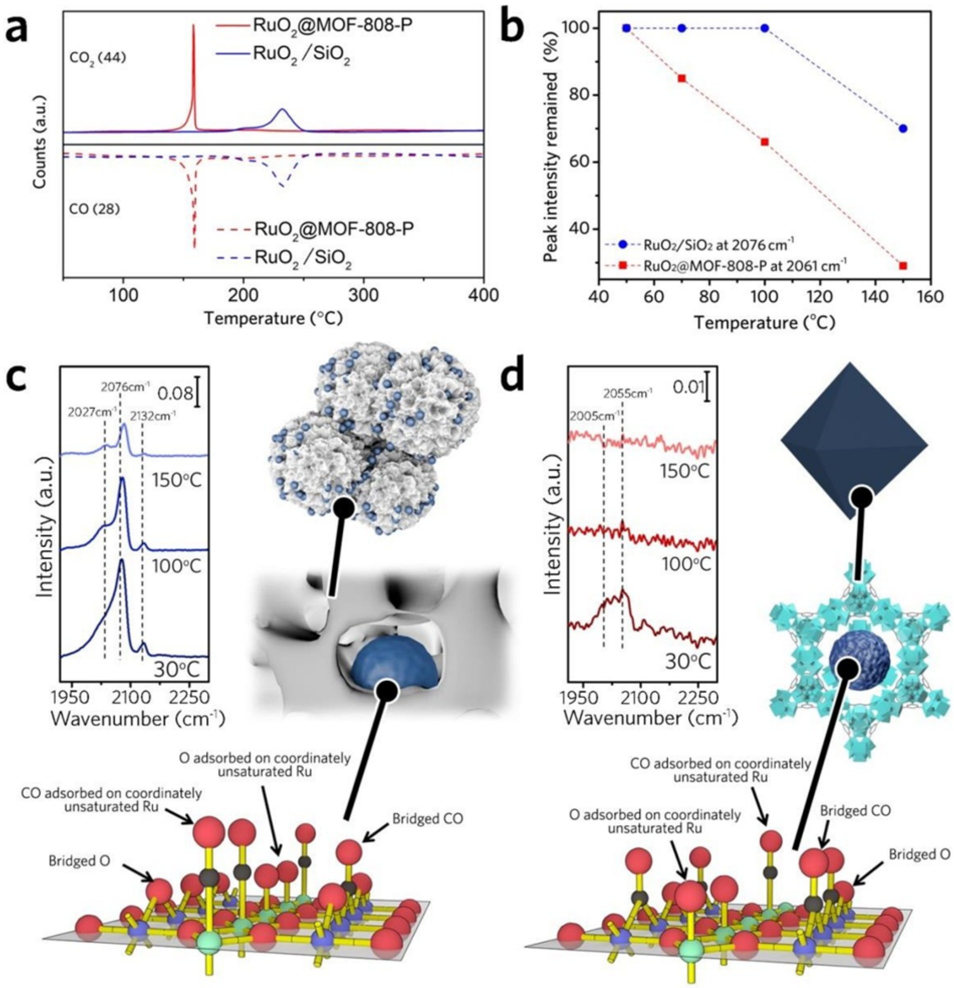

"Fig. 3 Adsorption modulation of CO and O on RuO_2 guest within MOF as compared to RuO_2 supported on mesoporous SiO_2 (Experimental methods are detailed in the Supplementary Information section 4.1): *(a) CO-TPR in flowing CO and (b) temperature-dependent desorption of surface-adsorbed CO as revealed by the DRIFTS peak intensity change with temperature in flowing Ar for RuO_2@MOF-808-P (in red) and RuO_2/SiO_2 (in blue). Temperature-dependent DRIFTS results in Ar for (c) RuO_2/SiO_2 and (d) RuO_2@MOF-808-P*

pretreated in the reaction gas (1 v/v% CO, 20 v/v% O_2, and 79 v/v% He) at room temperature. The RuO_2 (110) surface is taken as an example to assist our interpretation of the DRIFTS results in Table 1 (O in red, C in black, and green and blue for alternating rows of Ru with different {RuO_6} octahedral orientation)."

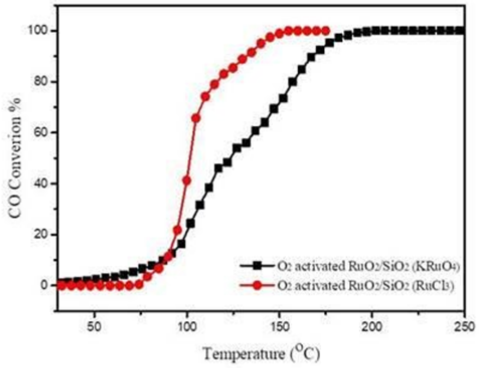

"**Figure S20. CO oxidation tests for O_2 activated RuO_2/SiO_2 ($RuCl_3$) and RuO_2/SiO_2 ($KRuO_4$).** *The catalysts were activated by O_2 gas at 250 °C for 1h. The weight hourly space velocity (WHSV) is 2000 $L\cdot g_{Ru}^{-1}\cdot h^{-1}$. The catalytic results indicated that, for impregnation method, the CO oxidation performance for RuO_2/SiO_2 with $RuCl_3$ is better than that for RuO_2/SiO_2 with $KRuO_4$. Therefore, this excludes the precursor contribution (i.e. $KRuO_4$) to the superior performance of RuO_2@MOF-808-P."*

2. In Figure 3c-d, the authors compared the IR signature of CO absorption on RuO_2-SiO_2 and RuO_2-MOF-808 and concluded "Ru-CO interactions are also weaker on the MOF-confined RuO_2 surface". However, DRIFTS IR signal generally arises from the reflection on material surface. In the case of nonporous RuO_2-SiO_2, the Ru active sites are located on the material surface, thus the IR signals are stronger. But in the case of RuO_2@MOF-808, the RuO_2 particles are wrapped inside the MOF channel. And this could be the real reason for the weaker DRIFTS IR signal. Did the authors do any transmissive IR? C-O stretching frequency shifts might be more indicative of such interaction strength but the authors did not observe any shift.

Response: We thank the reviewer for the suggestion. We agree with the reviewer that the DRIFTS IR signal intensity can be influenced by the support, especially when both supports are porous. That's why we only monitored the relative IR signal intensity change in situ for the same sample in each case (temperature-dependent desorption, Figure 3b). The intensities at different temperatures for the same sample can therefore be compared and normalized to the IR intensity at room temperature, as shown in Figure 3b. This reveals the weaker RuO_2-CO surface interaction in the MOF-confined sample.

We also agree with the reviewer that the relative C-O stretching frequency shifts are important. We had included this information previously only in Table 1, but to make it clearer, now we have also commented about it on page 13 in the manuscript: "*The shift of on-top CO stretching frequency (2076 cm^{-1} for RuO_2/SiO_2 versus 2055 cm^{-1} for RuO_2@MOF-808-P) is attributed to the disappearance of the densely-packed CO domains in RuO_2@MOF-808-P.[40,56] Weakened Ru-O interaction is supported by the change of bridging CO frequency (2027 cm^{-1} for RuO_2/SiO_2 versus 2005 cm^{-1} for RuO_2@MOF-808-P) showing fewer O surrounding the CO on the surface of the MOF-confined RuO_2.[40,56]*".

We did not obtain IR results in transmission mode, as the sample preparation process for transmissive IR (*i.e.* compressing powder samples into pellets) can destroy the MOF's structure closing the cavity/channel which accommodates the RuO_2. The structural

collapse and pore closing would inevitably influence the CO migration and catalytic reaction inside the MOF.

3. The authors determined the Ru content in RuO_2@MOF-808 by ICP-OES. Did they determine the amount of K? Because the authors used $KRuO_4$ as synthetic precursor. The amount of K can indicate the amount of residue $KRuO_4$ trapped in the channel through ionic interactions, and the surface charge properties of the RuO_2 particle. For example, high density of $(RuO_4^-)(K^+)$ functionalities that might give rise to the improved catalytic performance.

Response: We thank the reviewer for the questions. We performed ICP-OES for RuO_2@MOF-808-P as suggested by the reviewer. We confirm that the K content is insignificant (ca. 0.05 wt%) compared with Ru content (ca. 10 wt%) in the same sample. The result is further supported by the SEM-EDS spectrum (Figure S7, no peak at 3.314 keV for Kα) and the XPS survey scan (Figure S9, no peak between 290 eV and 300 eV for K 2p).

We add "*No significant potassium (K) residual can be found by ICP-OES in the RuO_2@MOF-808-P.*" on pages 9-10 in the manuscript with tracked changes accordingly.

4. How do the authors confirm the identity of RuO_2 particle inside the MOF channel? Because TEM images are not of high enough quality for determining accurate structure; did the authors take PXRD of the trapped RuO_2 particle?

Response: We agree that TEM images alone cannot confirm the identity of RuO_2 particles (guest) inside the MOF channels. We therefore characterized the RuO_2 guest through a combination of techniques, including PXRD (Figure S6), SEM-EDS (Figure S7), STEM-EDS (Figure S8), XPS (Figure S9 and S10), XAFS (Figure S11), high resolution STEM (Figure S12) and nitrogen adsorption measurements (Figure S5d). The relevant discussions were given in the manuscript on page 9 starting from "*We confirmed the preservation of the MOF host structure…*" and the captions of the above-mentioned figures in the Supplementary Information.

Briefly, PXRD patterns (Figure S6) indicate (i) the preservation of MOF's structure before and after the guest formation. Also (ii) if RuO_2 were incorporated in RuO_2@MOF-808-P, it would be very small (< 3 nm), as no significant peak for RuO_2 can be found due to very broad peak for very small particles/clusters and relatively small of amount of RuO_2 incorporated, which is found generally in guest@MOF systems.[4,17,18] Both EDS from SEM and STEM (Figure S7 and S8) as well as XPS (Figure S9) confirm the presence of Ru. XPS Ru $3p_{3/2}$ spectrum shows that the valence of Ru is IV (i.e. Ru^{+4}) with peak dominantly at 463.2 eV.[19,20] XAFS spectrum (Ru K-edge, Figure S11) reveals the domination of Ru-O. Although the quality of STEM images (Figure S12) is comparable with other systems (e.g. Ru@MOF-5[21]), the close atomic numbers of Zr (n = 40) and Ru (n = 44) make it difficult to image the RuO_2 from the Zr-based MOF by contrast. Nonetheless, we manage to spot out some diffraction fringes with inter-spacing of 2-2.5 Å, which matches well with $d_{(011),(101)}$ or/and $d_{(200),(020)}$ planes in RuO_2. Furthermore, reduction in pore volume shown in pore distributions (Figure S5, derived from the N_2 adsorption measurements) supports the guest occupation in the MOF.

5. Although the authors successfully incorporated the RuO_2 NPs into MOF-808-p, however, more examples are needed to show the generality of the synthetic approach. For example, can other NPs (such as Pt, Pd) be confined into the MOFs by such an approach?

Response: More examples are given in the Supplementary Information (Figure S22 in the previous version and Figure S25 in the current revised version). For the convenience to the reviewer, we also include Figure S25 at the end of the response. So far, we have demonstrated several guests formed in different hosts, *i.e.* RuO_2 and MnO_x in MOF-808-P, MnO_x in a different MOF (DUT-67) and RuO_2 in a zeolite (Zeolite Y).

We also provided a set of Pourbaix diagrams (Figure S26, prepared by Campbell and Whiteker[22]) of transition metals to illustrate the use of the PEGS method for other solid guests. Furthermore, the recently launched Materials Project (http://materialsproject.org/)[23–25] provides a wide range of Pourbaix diagrams in its database. Therefore, we would like to inspire more people to use and expand the PEGS method in the near future.

As a further demonstration, as suggested by the reviewer, we have added an example tutorial section in the Supplementary Material on the use of Pourbaix diagrams to predict the potential PEGS conditions for formation of Pt and Pd inside a MOF. The Pourbaix diagrams for Pt [left, [Pt] = 10^{-3} mol/kg (total aqueous mixture)] and Pd [right, [Pd] = 10^{-3} mol/kg (total aqueous mixture)] are constructed using the Materials Project[23–25] as shown in the Figure S27.

Briefly, since no stable bare Pt^{2+} is seen on the Pourbaix diagram for Pt, it would be very difficult to use the Pt^{2+} for host (*e.g.* MOF) impregnation. Extra stabilization with ligands would be required in the Pt precursor. This explains why Pt precursors such as $[Pt(NH_3)_4]Cl_2$ rather than $PtCl_2$ is used for preparing Pt@MOF via solution-based synthesis, where extra NH_3 is involved to stabilize the Pt(II) salt.[26]

In contrast, Pd^{2+} is more ready to be used as the mobile precursors for MOF impregnation under low pH (stable Pd^{2+} phase shown at low pH even at fairly high Pd^{2+} concentration, 10^{-3} mol/kg ≈ 1 mM). This Pourbaix diagram further rationalizes the preparation of some published examples of Pd@MOF[26–28] with Pd(II) salts, such as $Pd(NO_3)_2$ and Pd(II) acetylacetonate.

Note that the experimentally working concentrations of the precursors can be different from those predicted in Pourbaix diagrams, as the Pourbaix diagrams only consider the thermodynamically favourable states but the actual reaction will also take account of the actual precursor compounds used and the kinetics. Meanwhile, similar diagrams remain to be developed for the reactions in other solvents.

We include Figure S25-S27 at the end of this response (next page).

We have also modified the conclusion of the manuscript on page 16 starting from "*The PEGS method can be extended to other guests and nanoporous hosts…*"

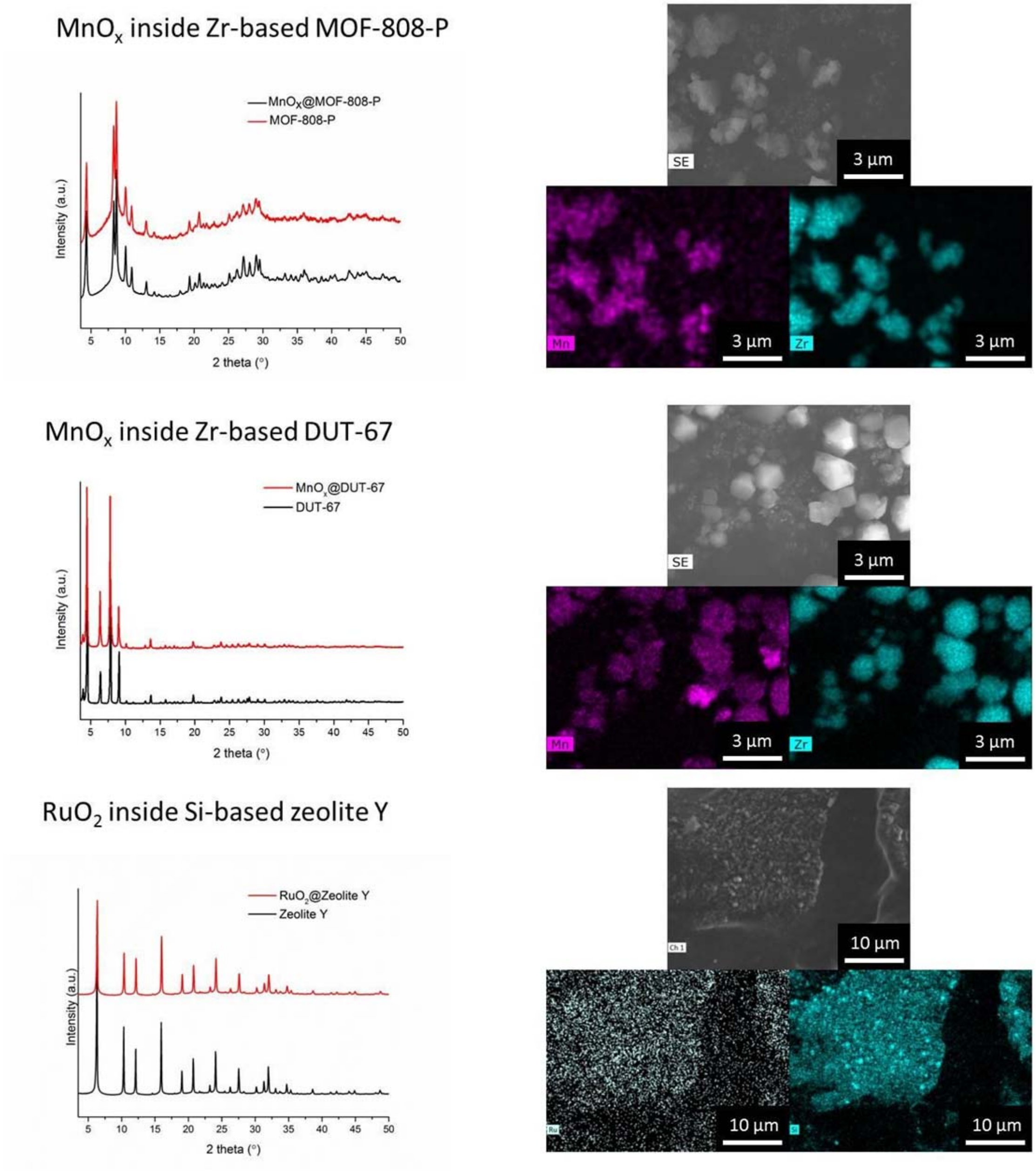

"***Figure S25. PXRD and SEM-EDS results for manganese oxide (MnO_x) in MOF-808-P (top) and another Zr-based MOF (i.e. DUT-67[50]) (middle) and RuO_2 in a commercially available zeolite Y[51] from Alfa Aesar (bottom).*** *Both of them are prepared using the methodology mentioned in this work (Figure S1) to demonstrate the general applicability of the guest incorporation concept for a range of nanoporous materials. Theprecursor for MnO_x is 20 mM $KMnO_4$ (aq); tBMP is used as the reducing agent for all the samples. The relevant characterization methods can be referred back to the Supplementary Information section 2.6.*"

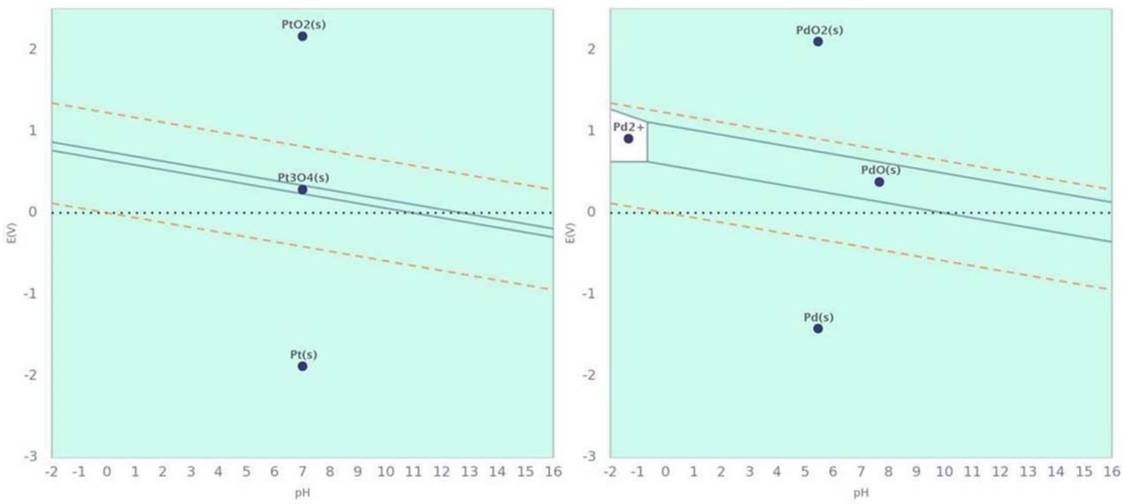

"Figure S27. Pourbaix diagrams for Pt [left, [Pt] = 10^{-3} mol/kg (total aqueous mixture)] and Pd [right, [Pd] = 10^{-3} mol/kg (total aqueous mixture)] are constructed using Materials Project.[52-54]"

6. To prove the generality of this synthetic method, the authors also need to try more MOFs as hosts. Why did the authors choose MOF-808-p but not MOF-808 as the host? Will other MOFs (such as MOF-5 and HKUST-1) also work as hosts?

Response: As mentioned above we have used other MOF hosts (*e.g.* DUT-67) and zeolite hosts (*e.g.* Zeolite Y) to demonstrate the PEGS method. These examples are shown in Figure S25 in the Supplementary Information. Figure S25 is also included in the response to the comment 5 (last comment).

The current PEGS synthesis needs aqueous solutions. MOF-808-like MOFs are stable over a wide pH range of 3 – 10 in aqueous solutions.[29] Meanwhile, they have relatively large aperture opening (ca. 14 Å) and cavity (ca. 18 Å) which makes reagents impregnation relatively easy.[30] According to Yaghi *et al.*, the synthesis of MOF-808-P[30] is modified from MOF-808[31] (i.e. an upgraded version). The major difference, in term of synthesis, between MOF-808-P and MOF-808 is the temperature and time used for growth. MOF-808-P grows at 140 °C for 2 days[30] whereas MOF-808 is achieved at 100 °C for 7 days[31]. Clearly, MOF-808-P requires much shorter time to be produce which significantly benefits the sample preparation.

We add "*The synthesis of MOF-808-P is modified from MOF-808 and requires shorter time.*[36,38]" on page 5 in the manuscript with tracked changes accordingly.

MOF-5 and HKUST-1, however, are quite vulnerable in aqueous solutions even in neutral pH.[32,33] Nonetheless, diagrams parallel to Pourbaix diagrams (Pourbaix-like diagrams) may be constructed for other solvents for water-free synthesis.

In general, other MOFs can also work as hosts as long as they are open-porous (with sufficiently large apertures) and stable in the post-synthetic modification conditions guided by the a relevant Pourbaix-like diagrams. A typical guidance provided by Howarth *et al.*[3] lists numerous candidates that are stable in difference ranges of pH as shown below. Although the guidance can provide a rough idea about the host candidates, experimental verification (*e.g.* PXRD in Figure S6) is necessary.

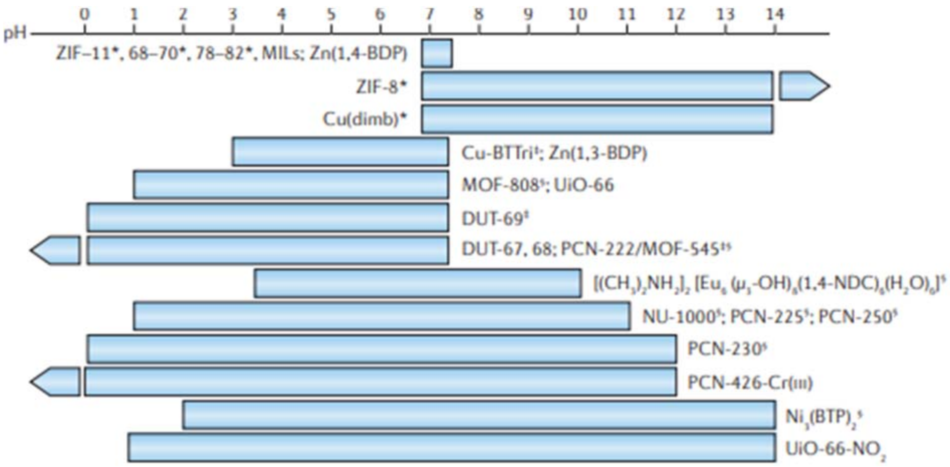

(MOF stability guidance provided by Howarth et al.[3] Copyright 2016 Nature Publishing Group.)

Reference:

1. Fang, Z., Bueken, B., De Vos, D. E. & Fischer, R. A. Defect-engineered metal-organic frameworks. *Angew. Chem. Int. Ed.* **54**, 7234–7254 (2015).

2. Cheetham, A. K., Bennett, T. D., Coudert, F. X. & Goodwin, A. L. Defects and disorder in metal organic frameworks. *Dalt. Trans.* **45**, 4113–4126 (2016).

3. Howarth, A. J. *et al.* Chemical, thermal and mechanical stabilities of metal–organic frameworks. *Nat. Rev. Mater.* **1**, 15018 (2016).

4. An, B. *et al.* Confinement of ultrasmall Cu/ZnO_x nanoparticles in metal–organic frameworks for selective methanol synthesis from catalytic hydrogenation of CO_2. *J. Am. Chem. Soc.* **139**, 3834–3840 (2017).

5. Corma, A., García, H. & Llabrés i Xamena, F. X. Engineering metal organic frameworks for heterogeneous catalysis. *Chem. Rev.* **110**, 4606–4655 (2010).

6. Prodinger, S. *et al.* Stability of zeolites in aqueous phase reactions. *Chem. Mater.* **29**, 7255–7262 (2017).

7. Freund, H. J., Meijer, G., Scheffler, M., Schlögl, R. & Wolf, M. CO oxidation as a prototypical reaction for heterogeneous processes. *Angew. Chem. Int. Ed.* **50**, 10064–10094 (2011).

8. Ertl, G. Reactions at surfaces: From atoms to complexity (Nobel lecture). *Angew. Chem. Int. Ed.* **47**, 3524–3535 (2008).

9. Lamberti, C., Zecchina, A., Groppo, E. & Bordiga, S. Probing the surfaces of heterogeneous catalysts by in situ IR spectroscopy. *Chem. Soc. Rev.* **39**, 4951–5001 (2010).

10. Kozachuk, O. *et al.* Multifunctional, defect-engineered metal-organic frameworks with ruthenium centers: Sorption and catalytic properties. *Angew. Chem. Int. Ed.* **53**, 7058–7062 (2014).

11. Mallat, T. & Baiker, A. Oxidation of alcohols with molecular oxygen on solid catalysts. *Chem. Rev.* **104**, 3037–3058 (2004).

12. Royer, S. & Duprez, D. Catalytic Oxidation of Carbon Monoxide over Transition Metal Oxides. *ChemCatChem* **3**, 24–65 (2011).

13. Min, B. K. & Friend, C. M. Heterogeneous gold-based catalysis for green chemistry: Low-temperature CO oxidation and propene oxidation. *Chem. Rev.* **107**, 2709–2724 (2007).

14. Over, H. Surface chemistry of ruthenium dioxide in heterogeneous catalysis and electrocatalysis: From fundamental to applied research. *Chem. Rev.* **112**, 3356–3426 (2012).

15. Anovitz, L. M. & Cole, D. R. Characterization and analysis of porosity and pore structures. *Rev. Mineral. Geochem.* **80**, 61–164 (2015).

16. Aßmann, J., Löffler, E., Birkner, A. & Muhler, M. Ruthenium as oxidation catalyst: bridging the pressure and material gaps between ideal and real systems in heterogeneous catalysis by applying DRIFT spectroscopy and the TAP reactor. *Catal. Today* **85**, 235–249 (2003).

17. Chen, L. *et al.* Selective catalytic performances of noble metal nanoparticle@MOF composites: The concomitant effect of aperture size and structural flexibility of MOF matrices. *Chem. Eur. J.* **23**, 11397–11403 (2017).

18. Jiang, H. *et al.* Au@ZIF-8 : CO oxidation over gold nanoparticles deposited to metal-organic framework. *J. Am. Chem. Soc.* **2**, 11302–11303 (2009).

19. Velázquez-Palenzuela, A. *et al.* Structural properties of unsupported Pt−Ru nanoparticles as anodic catalyst for proton exchange membrane fuel cells. *J. Phys. Chem. C* **114**, 4399–4407 (2010).

20. Zhang, X. & Chan, K. Y. Water-in-oil microemulsion synthesis of platinum-ruthenium nanoparticles, their characterization and electrocatalytic properties. *Chem. Mater.* **15**, 451–459 (2003).

21. Turner, S. *et al.* Direct imaging of loaded metal−organic framework materials (Metal@MOF-5). *Chem. Mater.* **20**, 5622–5627 (2008).

22. Campbell, J. A. & Whiteker, R. A. A periodic table based on potential-pH diagrams. *J. Chem. Educ.* **46**, 90 (1969).

23. Singh, A. K. *et al.* Electrochemical stability of metastable materials. *Chem. Mater.* **29**, 10159–10167 (2017).

24. Jain, A. *et al.* Commentary: The materials project: A materials genome approach to accelerating materials innovation. *APL Mater.* **1**, 011002 (2013).

25. Persson, K. A., Waldwick, B., Lazic, P. & Ceder, G. Prediction of solid-aqueous equilibria: Scheme to combine first-principles calculations of solids with experimental aqueous states. *Phys. Rev. B* **85**, 235438 (2012).

26. Juan-Alcañiz, J., Gascon, J. & Kapteijn, F. Metal–organic frameworks as scaffolds for the encapsulation of active species: State of the art and future perspectives. *J. Mater. Chem.* **22**, 10102-10118 (2012).

27. Meilikhov, M. *et al.* Metals@MOFs - Loading MOFs with metal nanoparticles for hybrid functions. *Eur. J. Inorg. Chem.* **2010**, 3701–3714 (2010).

28. Pascanu, V. *et al.* Sustainable catalysis: Rational Pd loading on MIL-101Cr-NH_2 for more efficient and recyclable Suzuki-Miyaura reactions. *Chem. Eur. J.* **19**, 17483–17493 (2013).

29. Zheng, H.-Q. et al. MOF-808: A metal–organic framework with intrinsic peroxidase-like catalytic activity at neutral pH for colorimetric biosensing. *Inorg. Chem.* **57**, 9096–9104 (2018).

30. Jiang, J. *et al.* Superacidity in sulfated metal–organic framework-808. *J. Am. Chem. Soc.* **136**, 12844–12847 (2014).

31. Furukawa, H. *et al.* Water adsorption in porous metal–organic frameworks and related materials. *J. Am. Chem. Soc.* **136**, 4369–4381 (2014).

32. Ming, Y. *et al.* Kinetic stability of MOF-5 in humid environments: Impact of powder densification, humidity level, and exposure time. *Langmuir* **31**, 4988–4995 (2015).

33. McHugh, L. N. *et al.* Hydrolytic stability in hemilabile metal–organic frameworks. *Nat. Chem.* (2018). doi:10.1038/s41557-018-0104-x

Reviewers' comments:

Reviewer #2 (Remarks to the Author):

The authors have properly addressed my concerns and I believe also those of the other referees. I remain enthusiastic about the quality and originality of the work and fully support publication in its current form.

Reviewer #3 (Remarks to the Author):

Please see attached file.

Editorial Note: Attached file follows on next page

Regarding the importance of this work and the generality of the synthetic approach, the authors did not address both issues. First, CO oxidation has been explored extensively (with too many publications) with no particular practical relevance. Second, the authors have not expanded the so called PEGS method to a broad range pf metal/metal oxide particles and other MOF hosts. Overall, the authors have not addressed key concerns raised in the previous round of review and this reviewer maintains his/her previous recommendation of rejecting this paper from *Nat. Commun.*

Response 1:

The authors responded to earlier critique of using a nonporous silica host by re-synthesizing mesoporous SiO_2 as the support. However, the authors cited a wrong reference for mesoporous silica. TEM images of RuO_2@SiO_2 in Figure S14 seem to show regular silica but not mesoporous silica. This contradictory statements/evidences raised more questions than addressing earlier concerns.

nanoparticle-loaded SiO_2

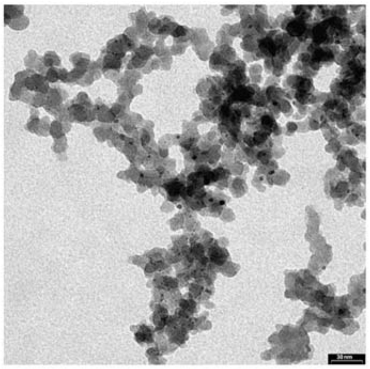

Fig. 1. Transmission electron micrograph of the Ru/SiO_2 catalyst.

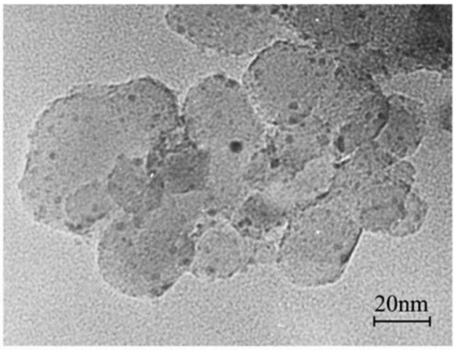

Ref 1: Catalysis Communications, 2003, 4, 91-96.

Ref 2: Catalysis Today, 2003, 85, 235-249

Mesoporous SiO_2

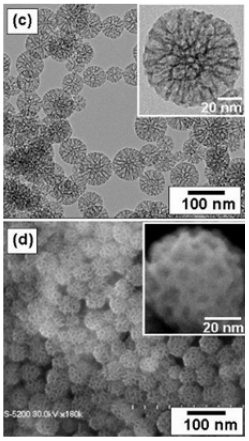

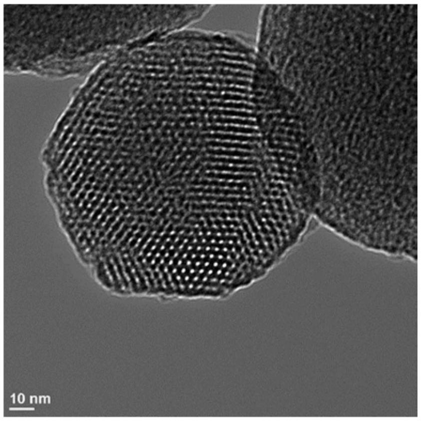

Ref 3: Microporous and Mesoporous Materials. 120 (3): 447–453.

doi:10.1016/j.micromeso.2008.12.019

Nanoparticle loaded Mesoporous SiO_2

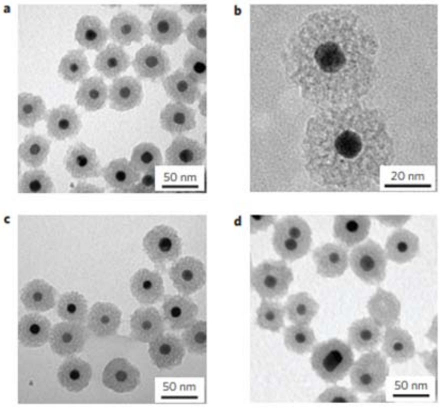

Ref 4: Nature materials, 2009, 8, 126-131.

Ref 5: Chem. Commun., 2010, 46, 6524–6526

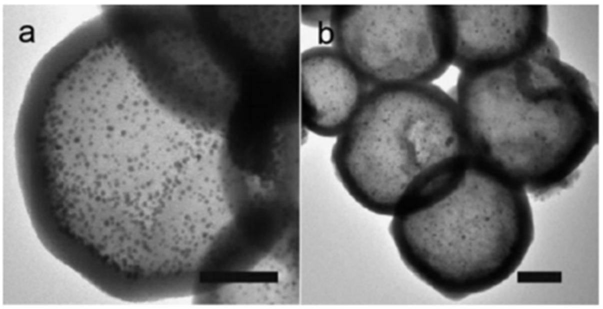

TEM in this work (Figure S14)

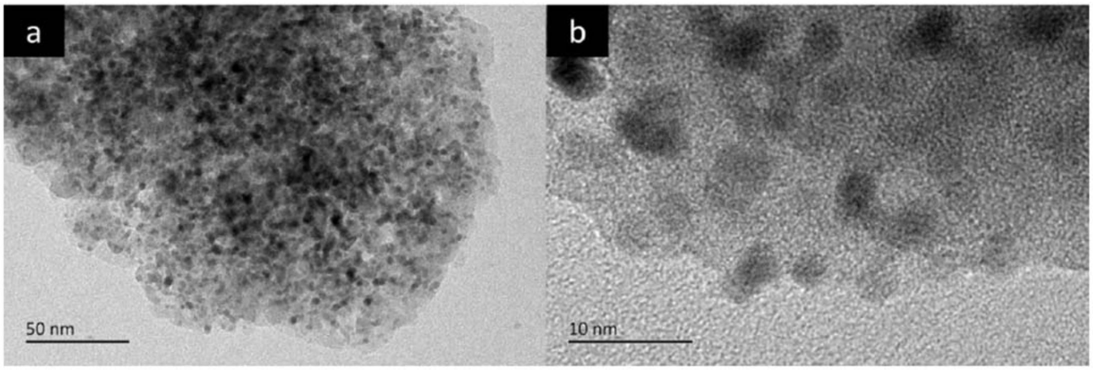

Figure S14. HRTEM images about the Ru/SiO_2. *The Ru nanoparticles distributed uniformly on the SiO_2 supports with an average diameter between ca. 3 and ca. 5 nm.*

TEM images in this work look like regular SiO_2 but not mesoporous silica. Since this is a key control experiment, the authors clearly have not addressed the concern of using a wrong control to support their claim.

Response 2:

The authors argued that no transmissive IR results could be obatineddue to structural collapse and pore closing during dry-powder sample preparation. This is not the case based on this reviewer's personal experience. The authors should use supercritical drying method to prevent structural collapse during MOF drying process. Again, the authors did not address this concern.

Response 4:

The authors did not completely address this issue. The RuO2 particles could be outside of MOF channels and they are not really confined in MOF channels (there is insufficient information on this issue as well as on how uniform the particles are).

Response 5:

Although the author provided two examples (Ru, Mn) for the PEGS method, the generality of this method still has not been fully established. Pd NPs, for example, need to be tested with this method. The performance of such particles in reactions much more interesting than CO oxidation needs to be demonstrated.

1. *Regarding the importance of this work and the generality of the synthetic approach, the authors did not address both issues. First, CO oxidation has been explored extensively (with too many publications) with no particular practical relevance. Second, the authors have not expanded the so called PEGS method to a broad range pf metal/metal oxide particles and other MOF hosts. Overall, the authors have not addressed key concerns raised in the previous round of review and this reviewer maintains his/her previous recommendation of rejecting this paper from Nat.Commun.*

Response: The authors are thankful for the reviewer's constructive comments. The relevant points have been further improved after the 2nd revision. We believe the conversation with the reviewer is a valuable one to publish together with the paper and we would support this as a resource for readers interested in the work. We believe that both the importance of this work and the generality of the synthetic approach are now sufficiently presented in both the revised manuscript and SI.

As for the practical relevance of CO oxidation catalysis, we have clearly stated both in the manuscript (p.12) and the previous response (p.4) that CO oxidation is practically important for automotive exhaust emission control. We have now added also - production of CO-free hydrogen for ammonia synthesis and fuel cells (see track changes, p.12). Still, the most important feature of the CO oxidation reaction is that it is a simple and well-understood reaction, so it is possible to use it to detect changes in the mechanism. We believe others will use the method to study other reactions that are outside the scope of this work.

We have demonstrated the PEGS method with oxide guests (RuO_2 and MnO_2) and with MOF hosts (MOF-808-P and DUT-67) and other hosts (zeolite Y). On the reviewer's suggestion, in this revision, we have also used the PEGS method to load metal guest (Pd) into/onto MOF-808-P (and present the results at the end of Supplementary Information Section 5 on pages 31-33). The number of examples should be sufficient for the work, particularly as an initial report. More importantly, we have provided a detailed tutorial guidance about the PEGS method in the Supplementary Information Section 1 with a flow chart in Supplementary Fig. 1 (right). We have also attached the Pourbaix diagrams for many transition metal elements in Supplementary Fig. 26 to support the feasibility of the PEGS method. We believe these diagrams cover the many important classes of compounds, so readers can use the PEGS methods to prepare new specific systems which are meaningful to them.

Additionally, note that the loading control is an additional bonus enabled by the PEGS method in the case of using tBMP for RuO_2. The PEGS method by itself refers to the rational synthesis based on Pourbaix diagrams. The authors have improved the introduction to avoid any related confusion.

2. ***Response 1**: The authors responded to earlier critique of using a nonporous silica host by re-synthesizing mesoporous SiO_2 as the support. However, the authors cited a wrong reference for mesoporous silica. TEM images of RuO_2@SiO_2 in Figure S14 seem to show regular silica but not mesoporous silica. This contradictory statements/evidences raised more questions than addressing earlier concerns.*

nanoparticle-loaded SiO2

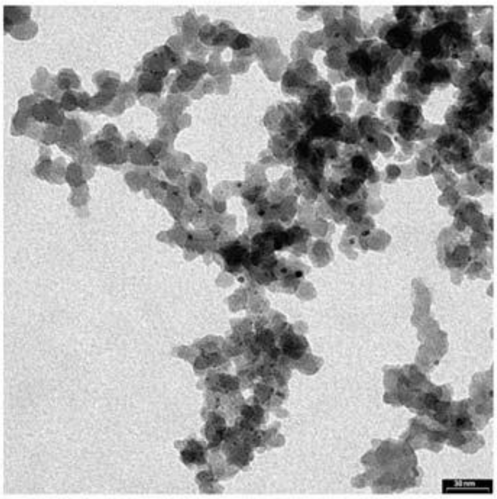

Fig. 1. Transmission electron micrograph of the Ru/SiO_2 catalyst.

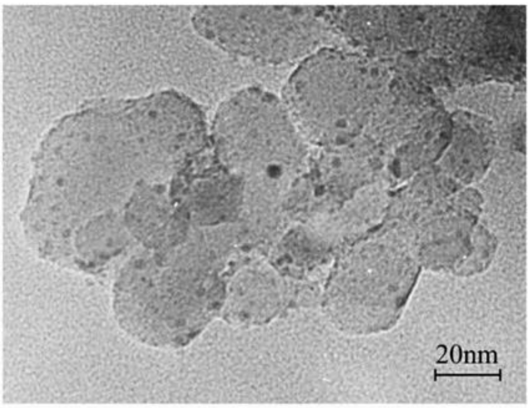

Ref 1: Catalysis Communications, 2003, 4, 91-96.

Ref 2: Catalysis Today, 2003, 85, 235-249

Mesoporous SiO₂

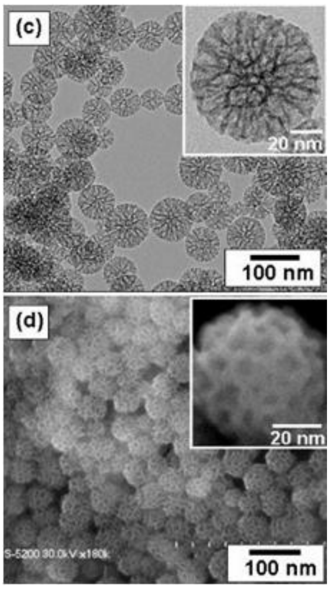

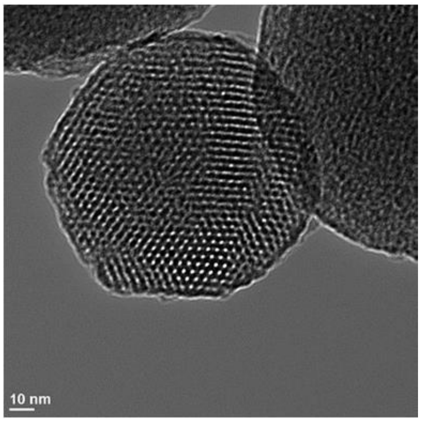

Ref 3: Microporous and Mesoporous Materials. 120 (3): 447–453.

doi:10.1016/j.micromeso.2008.12.019

Nanoparticle loaded Mesoporous SiO_2

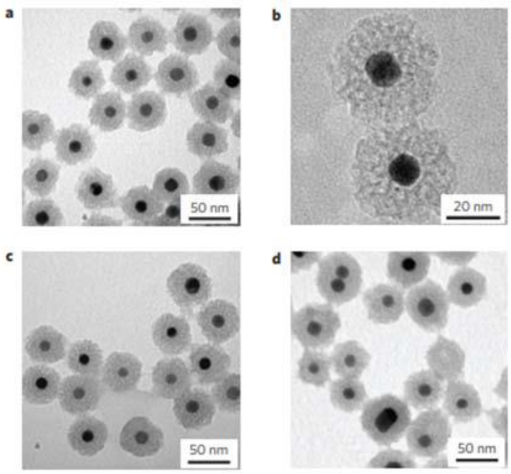

Ref 4: Nature materials, 2009, 8, 126-131.

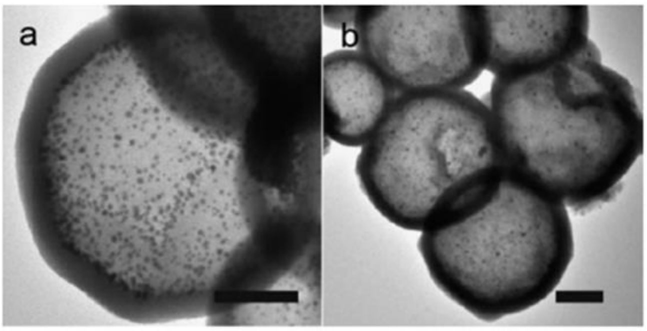

Ref 5: Chem. Commun.,2010, 46, 6524–6526

TEM in this work (Figure S14)

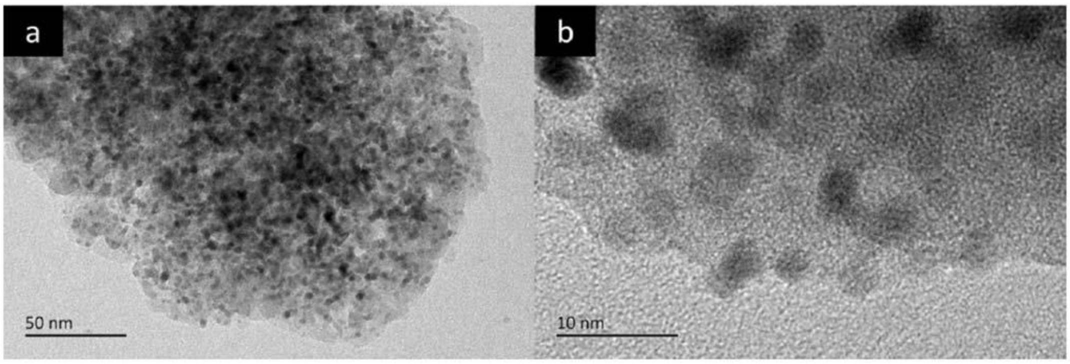

Figure S14. HRTEM images about the Ru/SiO_2. *The Ru nanoparticles distributed uniformly on the SiO_2 supports with an average diameter between ca. 3 and ca. 5 nm.*

TEM images in this work look like regular SiO_2 but not mesoporous silica. Since this is a key control experiment, the authors clearly have not addressed the concern of using a wrong control to support their claim.

Response: The authors would like to clarify that the references (Ref 1 & 2 in this comment (58 and 56 in the updated manuscript) are **NOT** referring tothe silica-based support used. Ref 1: Catalysis Communications, 2003, 4, 91-96 is the reference for the **conventional impregnation method**. Ref 2: Catalysis Today, 2003, 85, 235-249 is the reference for the **DRIFTS results interpretations**.

We also use the RuO_2/SiO_2 sample in the manuscript only to show RuO2@MOF-808-P has (i) higher catalytic CO oxidation performance compared to common catalysts, and (ii) results in weaker interactions between CO/O and RuO_2. Furthermore, we have shown that RuO2@MOF-808-P is superior to most of Ru-based or guest@MOF-based catalysts for CO oxidation (Supplementary Table 1). Though influence of different pores on the catalysis is NOT the primary concern of this work we have actually used mesoporous SiO_2 supportin this work.

Mesopores (for catalysis) are 2-50 nm pores, as defined by IUPAC (http://goldbook.iupac.org/html/M/M03853.html, accessed on 07 Nov 2018), "Pore of intermediate size. [Those with widths exceeding about 0.05 µm or 50 nm (500 Å) are called macropores; those with widths not exceeding about 2 nm (20 Å) are called micropores]." The reviewer may have a more strict definition about mesoporous silica, where pores should be visible from TEM images (Ref 3: Microporous and Mesoporous Materials. 120 (3): 447–453). Ordered silicas are only a subset of the mesoporous silicas.Mesoporosity, is best characterised by gas adsorption experiments.[1] The shape of N_2 isotherm and its hysteresis pattern is very useful to support the pore size in porous materials:

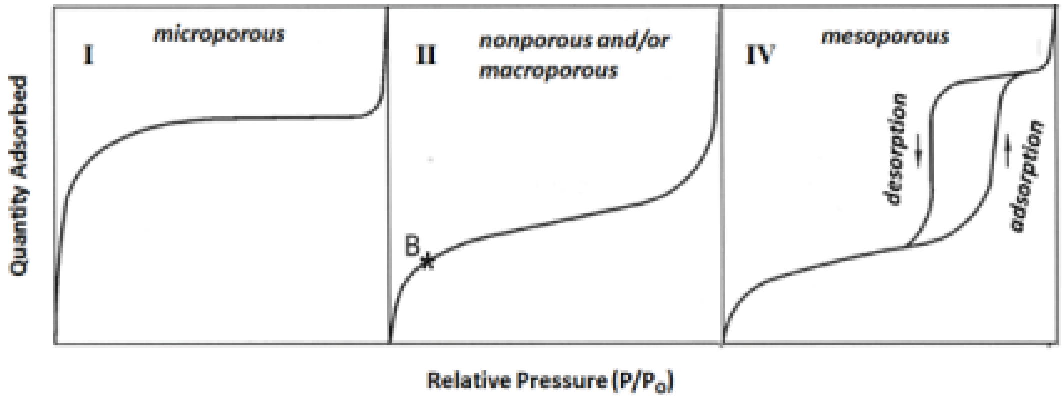

Response Figure 1. Typical N_2 isotherm shapes for porous materials. The figure is from Rev. Mineral. Geochem. 80, 61–164 (2015).

The N_2 isotherm shape for the silica material in this work is very similar to the typical shape for mesoporous materials:

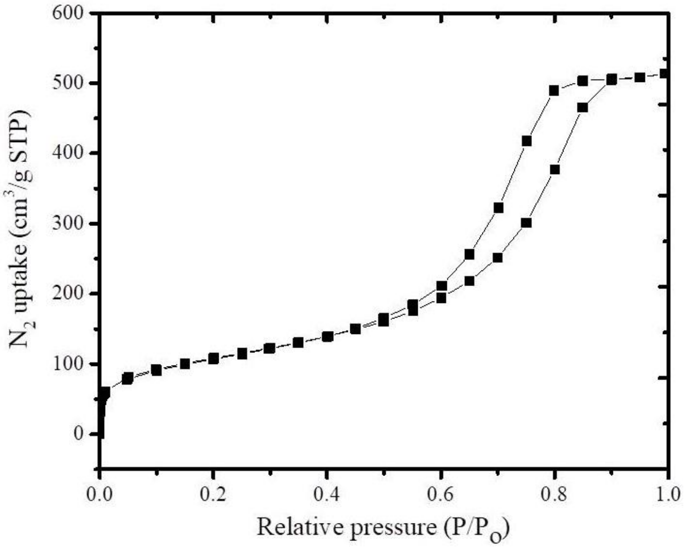

"**Supplementary Fig. 16. Nitrogen physisorption isotherm of the SiO_2 support,** which has typical Type IV isotherm shape[37] showing the mesoporous SiO_2 with 356.1 m^2/g surface area. Further analysis shows the pore diameter is 5-10 nm. Source data are provided as a Source Data file."

In fact, the N_2 isotherm shape matches well with that mentioned in the reviewer's reference (Ref 3: Microporous and Mesoporous Materials. 120 (3): 447–453):

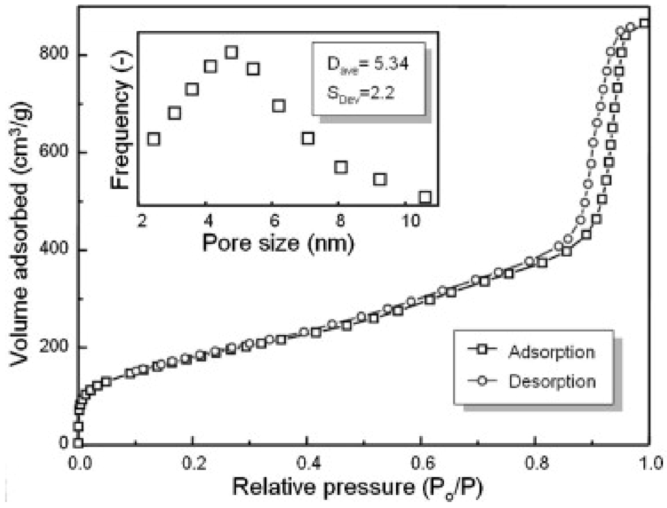

Response Figure 2. N_2 isotherm from Ref 3: Microporous and Mesoporous Materials. 120 (3): 447–453.

Since the silica we used is quite amorphous (as revealed by Supplementary Fig. 15) with no regular pores, the mesopores cannot be easily seen. **To improve the preciseness of the work, we change the term "mesoporous SiO_2" to "amorphous SiO_2 with mesoporosity" in the updated manuscript.**

We want to point out for the professional level of this discussion that the righ-hand side figure below is NOT from the reference cited by the reviewer.

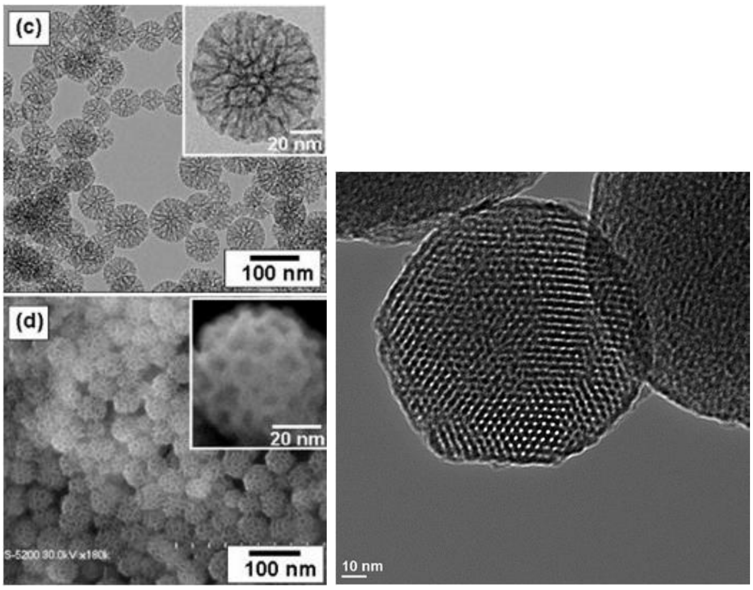

Ref 3: Microporous and Mesoporous Materials. 120 (3): 447–453. doi:10.1016/j.micromeso.2008.12.019

Perhaps the reviewer collected these images from Wikipedia and lumped them into a single citation (Ref 3: Microporous and Mesoporous Materials. 120 (3): 447–453) .

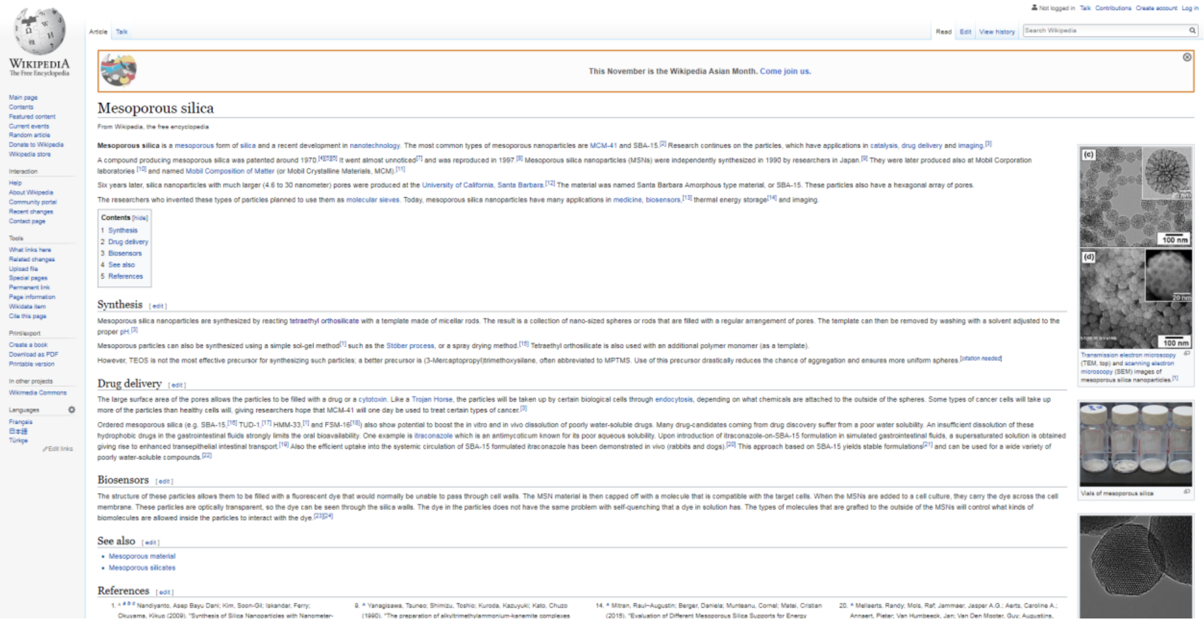

Article Talk Read Edit View history Search Wikipedia

This November is the Wikipedia Asian Month. Come join us.

Mesoporous silica

From Wikipedia, the free encyclopedia

Mesoporous silica is a mesoporous form of silica and a recent development in nanotechnology. The most common types of mesoporous nanoparticles are MCM-41 and SBA-15.[2] Research continues on the particles, which have applications in catalysis, drug delivery and imaging.[3]

A compound producing mesoporous silica was patented around 1970.[4][5][6] It went almost unnoticed[7] and was reproduced in 1997.[8] Mesoporous silica nanoparticles (MSNs) were independently synthesized in 1990 by researchers in Japan.[9] They were later produced also at Mobil Corporation laboratories [10] and named Mobil Composition of Matter (or Mobil Crystalline Materials, MCM).[11]

Six years later, silica nanoparticles with much larger (4.6 to 30 nanometer) pores were produced at the University of California, Santa Barbara.[12] The material was named Santa Barbara Amorphous type material, or SBA-15. These particles also have a hexagonal array of pores.

The researchers who invented these types of particles planned to use them as molecular sieves. Today, mesoporous silica nanoparticles have many applications in medicine, biosensors,[13] thermal energy storage[14] and imaging.

Contents [hide]
1 Synthesis
2 Drug delivery
3 Biosensors
4 See also
5 References

Synthesis [edit]

Mesoporous silica nanoparticles are synthesized by reacting tetraethyl orthosilicate with a template made of micellar rods. The result is a collection of nano-sized spheres or rods that are filled with a regular arrangement of pores. The template can then be removed by washing with a solvent adjusted to the proper pH.[3]

Mesoporous particles can also be synthesized using a simple sol-gel method[1] such as the Stöber process, or a spray drying method.[15] Tetraethyl orthosilicate is also used with an additional polymer monomer (as a template).

However, TEOS is not the most effective precursor for synthesizing such particles; a better precursor is (3-Mercaptopropyl)trimethoxysilane, often abbreviated to MPTMS. Use of this precursor drastically reduces the chance of aggregation and ensures more uniform spheres.[citation needed]

Drug delivery [edit]

The large surface area of the pores allows the particles to be filled with a drug or a cytotoxin. Like a Trojan Horse, the particles will be taken up by certain biological cells through endocytosis, depending on what chemicals are attached to the outside of the spheres. Some types of cancer cells will take up more of the particles than healthy cells will, giving researchers hope that MCM-41 will one day be used to treat certain types of cancer.[3]

Ordered mesoporous silica (e.g. SBA-15,[16] TUD-1,[17] HMM-33,[1] and FSM-16[18]) also show potential to boost the in vitro and in vivo dissolution of poorly water-soluble drugs. Many drug-candidates coming from drug discovery suffer from a poor water solubility. An insufficient dissolution of these hydrophobic drugs in the gastrointestinal fluids strongly limits the oral bioavailability. One example is itraconazole which is an antimycoticum known for its poor aqueous solubility. Upon introduction of itraconazole-on-SBA-15 formulation in simulated gastrointestinal fluids, a supersaturated solution is obtained giving rise to enhanced transepithelial intestinal transport.[19] Also the efficient uptake into the systemic circulation of SBA-15 formulated itraconazole has been demonstrated in vivo (rabbits and dogs).[20] This approach based on SBA-15 yields stable formulations[21] and can be used for a wide variety of poorly water-soluble compounds.[22]

Biosensors [edit]

The structure of these particles allows them to be filled with a fluorescent dye that would normally be unable to pass through cell walls. The MSN material is then capped off with a molecule that is compatible with the target cells. When the MSNs are added to a cell culture, they carry the dye across the cell membrane. These particles are optically transparent, so the dye can be seen through the silica walls. The dye in the particles does not have the same problem with self-quenching that a dye in solution has. The types of molecules that are grafted to the outside of the MSNs will control what kinds of biomolecules are allowed inside the particles to interact with the dye.[23][24]

See also [edit]

- Mesoporous material
- Mesoporous silicates

References [edit]

Transmission electron microscopy (TEM, top) and scanning electron microscopy (SEM) images of mesoporous silica nanoparticles.[1]

Vials of mesoporous silica

Response Figure 3. Screenshot of the Wikipedia page for “Mesoporous silica” (http://en.wikipedia.org/wiki/Mesoporous_silica, accessed on 07 Nov 2018).

We believe the IUPAC definition of mesopores is more useful and the standard to abide to rather than quotations from Wikipedia.

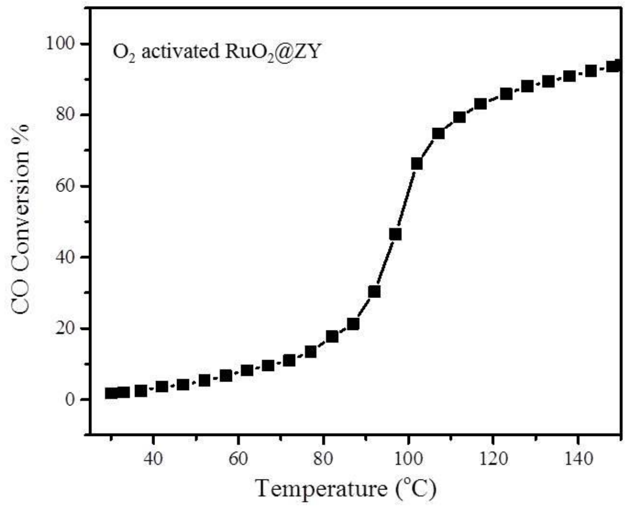

Response Figure 4. **CO oxidation test for O_2 activated RuO_2@ZY.** The catalysts were activated by O_2 gas at 150 ^oC for 1h. The reaction gas is composed by 1% CO, 20 % O_2 and 1% N_2 balanced with He. The WHSV is 2000 $L{\cdot}g_{Ru}^{-1}{\cdot}h^{-1}$.

As a further verification, we tested RuO_2@ZY as the catalyst for CO oxidation. RuO_2@ZY was prepared using a similar methodology ($KRuO_4$ as the precursor; tBMP as the reducing agent) as to RuO_2@MOF-808-P. Its PXRD and SEM-EDS results are provided in Supplementary Fig. 25. Its CO oxidation performance (Response Fig. 4) is similar to that for RuO_2/SiO_2. This further supports that MOF is a superior support for RuO_2 to catalyse CO oxidation.

3. ***Response 2:*** *The authors argued that no transmissive IR results could be obtained due to structural collapse and pore closing during dry-powder sample preparation. This is not the case based on this reviewer’s personal experience. The authors should use supercritical drying method to prevent structural collapse during MOF drying process. Again, the authors did not address this concern.*

Response: The authors have clearly explained the reasons for not having the transmissive measurements in the previous response on page 6 by saying “We did not obtain IR results in transmission mode, as the sample preparation process for transmissive IR (*i.e.* compressing

powder samples into pellets) can destroy the MOF's structure closing the cavity/channel which accommodates the RuO2." This has nothing to do with MOF drying process.

4. ***Response 4:*** *The authors did not completely address this issue. The RuO_2 particles could be outside of MOF channels and they are not really confined in MOF channels (there is insufficient information on this issue as well as on how uniform the particles are).*

Response: In the updated version, the authors discussed the clear difference between RuO_2 deposited both inside and outside the MOF, and RuO_2 loaded mostly inside the MOF. See p.9 in the manuscript, starting from "To demonstrate the loading position control,…". The relevant results are given in the updated Fig. 2:

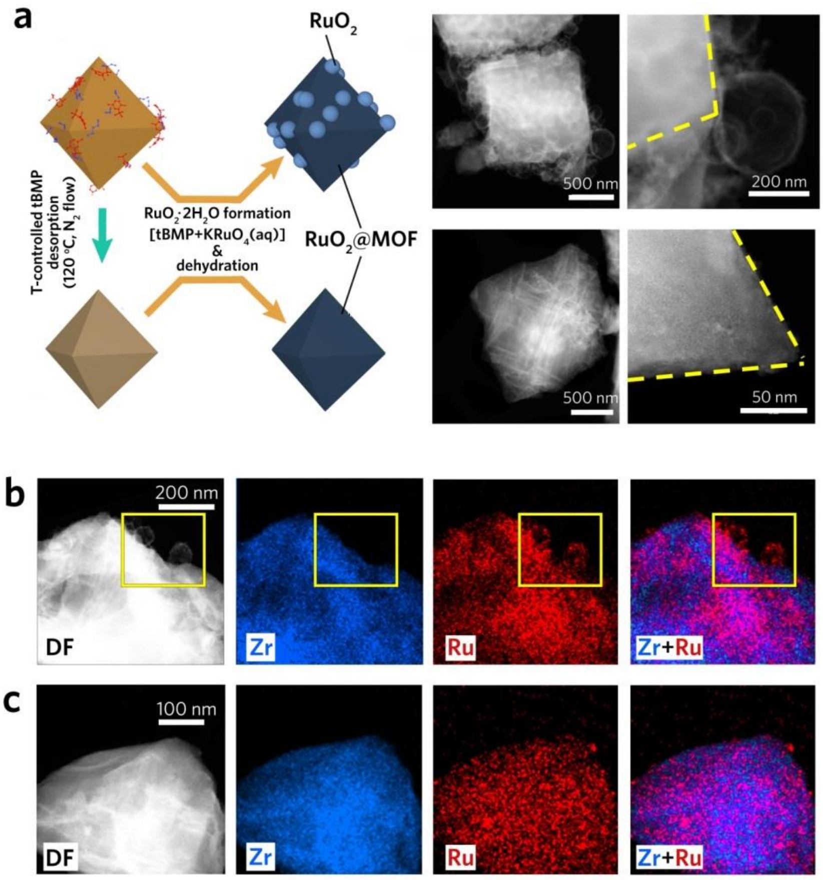

"**Fig. 2 Controllable RuO_2 guest formation inside (or both inside and outside) MOF-808-P:** (a) RuO_2 can be formed both inside and outside the MOF, or only inside the MOF (i.e. RuO_2@MOF-808-P) via temperature-controlled selective desorption of the tBMP molecules outside the MOF. DF-STEM images to the right show core-shell structures on the outer surface of the MOF crystals (top, for RuO_2 formed inside and outside the MOF) vs. clean MOF crystal edges (bottom, for RuO_2 loaded mostly inside the MOF). The deposition is further verified by STEM-EDS Zr and Ru mappings for (b) RuO_2 formed inside and outside the MOF and (c) RuO_2 loaded mostly inside the MOF. The yellow frames in (b) highlight the Ru-based core-shell structures. Raw images are provided as a Source Data file."

The outer surface deposition of tBMP can be controlled by temperature-controlled selective desorption. This almost completely eliminates theproduction of RuO_2 nano-entities on the outside the RuO_2@MOF-808-P see panel b (with) vs. panel c (without) in Fig. 2 above.

The updated Fig. 2 results are put in the context of significant results already reported in the manuscript. The characterizations/confirmation of RuO_2 inside the MOF have been discussed both in the manuscript and the previous response. Manuscript - (on pages 8 and 9 starting from “We confirmed the preservation of the MOF host structure throughout the synthesis of RuO_2@MOF-808-P...”), previous response (page 7, starting from “We therefore characterized the RuO_2 guest through a combination of techniques…”). A significant amount of the results in the SI were already dedicated to this (Supplementary Figs. 5-12)

The authors had also summarised the key findings for characterizing/confirming RuO_2 inside the MOF of the numerous experimental results in the following logical structureas below:

(1) **PXRD (Supplementary Fig. 6)**: MOF is still there;
(2) **Nitrogen adsorption measurements (Supplementary Fig. 5)**: Pores in the MOF are occupied by guest;
(3) **SEM-EDS and STEM-EDS (Supplementary Figs. 7 and 8)**: the guest is Ru-based compound;
(4) **XPS (Supplementary Figs. 9 and 10)**: the Ru for the guest is Ru^{4+};
(5) **XAFS (Supplementary Fig. 11)**: Ru-O is dominating, and the guest should be RuO_2;
(6) **DF-STEM (Supplementary Fig. 12)**: electron diffraction fringes match that for tetragonal RuO_2 (space group: $P4_2/mnm$).

The RuO_2 guest loading distributions are shown on a number of MOF particles (Supplementary Fig. 7) and on a single MOF particle (Supplementary Fig. 8). Almost all the MOF particles are loaded with RuO_2 (Supplementary Fig. 7); the RuO_2 loading on a single MOF particle (Supplementary Fig. 8) is not extremely uniform but acceptable for the application.

5. ***Response 4:*** *Although the author provided two examples (Ru, Mn) for the PEGS method, the generality of this method still has not been fully established. Pd NPs, for example, need to be tested with this method. The performance of such particles in reactions much more interesting than CO oxidation needs to be demonstrated.*

Response: We appreciate the reviewer’s suggestion which has significantly improved the paper. To show the generality of the method and include the wide variety of metal catalysts, we have used the PEGS method to guide the synthesis of a Pd-loaded MOF-808-P without destroying the MOF. (See pp 31-33 in SI, starting from “In contrast, according to the Pourbaix diagram for Pd …” We characterized the resulting Pd@MOF-808-P – Suppl. Fig. 28 and 29 in SI. Note that in this case the loading position is not controlled, it is not part of the PEGS method as stated in the introduction of the manuscript. We found metallic Pd is loaded both inside and outside the MOF from the STEM-EDS mappings (Supplementary Fig. 28). Some Pd particles can agglomerate on the outer surface of the MOF (without MOF pore confinement). The large metallic Pd particles can also be characterised by PXRD (Supplementary Fig. 29).As this is a paper for a synthetic design methodology, and we have shown changes in mechanism of Ru-based catalysts, we have not characterized the

reactivity and the selectivity of this new compound. This would be the subject of future work.

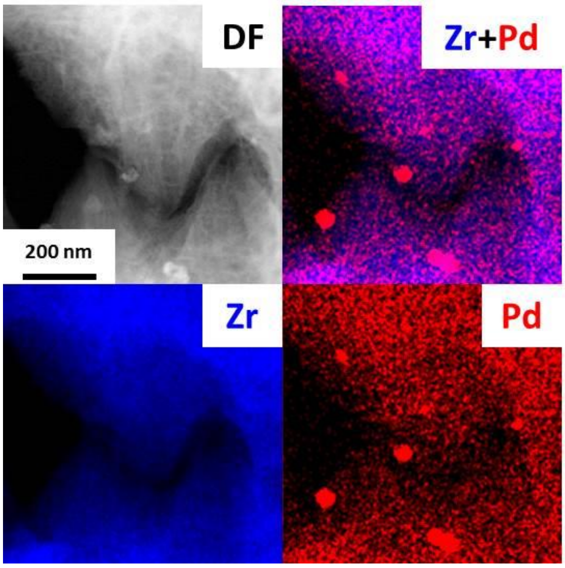

"**Supplementary Fig. 28. A DF-STEM image for Pd@MOF-808-P and its corresponding STEM-EDS mappings for Zr and Pd.** Raw images are provided as a Source Data file."

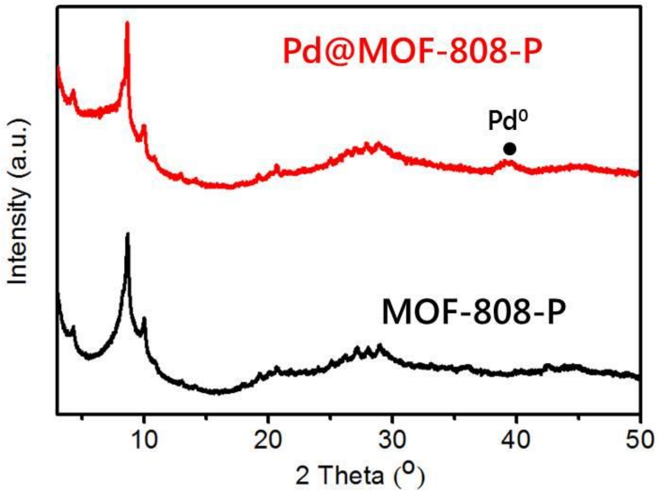

"**Supplementary Fig. 29. PXRD patterns for MOF-808-P and Pd loaded MOF-808-P (i.e. Pd@MOF-808-P).** The PXRD results were collected on a Rigaku D/Max 2500 diffractometer using a Cu Kα (λ=1.5406 Å) radiation source and scanning rate of 1°/min. Source data are provided as a Source Data file."

Reference

1. Anovitz, L. M. & Cole, D. R. Characterization and analysis of porosity and pore structures. *Rev. Mineral. Geochem.* **80**, 61–164 (2015).
